# Suites of Terpene Synthases Explain Differential Terpenoid Production in Ginger and Turmeric Tissues

**DOI:** 10.1371/journal.pone.0051481

**Published:** 2012-12-18

**Authors:** Hyun Jo Koo, David R. Gang

**Affiliations:** 1 Department of Plant Sciences and Bio5 Institute, The University of Arizona, Tucson, Arizona, United States of America; University of South Florida College of Medicine, United States of America

## Abstract

The essential oils of ginger (*Zingiber officinale*) and turmeric (*Curcuma longa*) contain a large variety of terpenoids, some of which possess anticancer, antiulcer, and antioxidant properties. Despite their importance, only four terpene synthases have been identified from the Zingiberaceae family: (+)-germacrene D synthase and (*S*)-β-bisabolene synthase from ginger rhizome, and α-humulene synthase and β-eudesmol synthase from shampoo ginger (*Zingiber zerumbet*) rhizome. We report the identification of 25 mono- and 18 sesquiterpene synthases from ginger and turmeric, with 13 and 11, respectively, being functionally characterized. Novel terpene synthases, (−)-caryolan-1-ol synthase and α-zingiberene/β-sesquiphellandrene synthase, which is responsible for formation of the major sesquiterpenoids in ginger and turmeric rhizomes, were also discovered. These suites of enzymes are responsible for formation of the majority of the terpenoids present in these two plants. Structures of several were modeled, and a comparison of sets of paralogs suggests how the terpene synthases in ginger and turmeric evolved. The most abundant and most important sesquiterpenoids in turmeric rhizomes, (+)-α-turmerone and (+)-β-turmerone, are produced from (−)-α-zingiberene and (−)-β-sesquiphellandrene, respectively, via α-zingiberene/β-sesquiphellandrene oxidase and a still unidentified dehydrogenase.

## Introduction

Ginger (*Zingiber officinale* Rosc.) and turmeric (*Curcuma longa* L.) have been used for centuries to treat human ailments. Ginger is effective against symptoms of the common cold, fever, rheumatic disorders, gastrointestinal complications, motion sickness, diabetes, and cancer [1]. It has anti-bacterial [2] and anti-fungal [3] activities. Many of these medicinal activities including anti-cancer and anti-inflammatory [4] are believed to be due to the presence of active phenolic compounds such as the gingerols, paradols and shogaols [5], [6], [7]. However, terpenoids from ginger have also been reported to have important human health roles. For example, β-elemene arrests the cell cycle and induces apoptotic cell death in lung cancer cells [8] and elemene aids patients with chylothorax [9]. Zingiberene as well as [6]-gingerol significantly inhibited gastric lesions [10] and further research revealed (−)-β-sesquiphellandrene, β -bisabolene, *ar*-curcumene and 6-shogaol as anti-ulcer active principles in ginger [11]. Turmeric has anti-inflammatory [12] and anti-cancer [13] properties, which have been reported to be mainly due to the presence of curcumin, a diarylheptanoid. On the other hand, turmeric oil, reported to contain *ar*-turmerone, turmerone and curlone, showed antioxidant effects and anti-mutagenic action [14]. Turmeric oil, consisting largely of terpenoids, also has anti-bacterial activity [15]. Both curcuminoids and sesquiterpenoids in turmeric exhibit hypoglycemic effects via peroxisome proliferator-activated receptor-γ (PPAR-γ) activation and suppress an increase in blood glucose levels in type 2 diabetic KK-Ay mice. The effect was synergistic when both curcuminoids and sesquiterpenoids in turmeric were applied together [16]. *ar*-Turmerone from turmeric oil displays anti-tumorigenesis activity, inhibiting cell proliferation and activating *ar*-turmerone-mediated apoptotic protein in human lymphoma U937 cells [17]. It was also found that apoptosis was selectively induced by *ar*-turmerone in human leukemia Molt 4B and HL-60 cells, but not in human stomach cancer KATO III cells. *ar*-Turmerone also has antiplatelet activities that can prevent and treat arteriol thrombosis [18].

**Figure 1:**
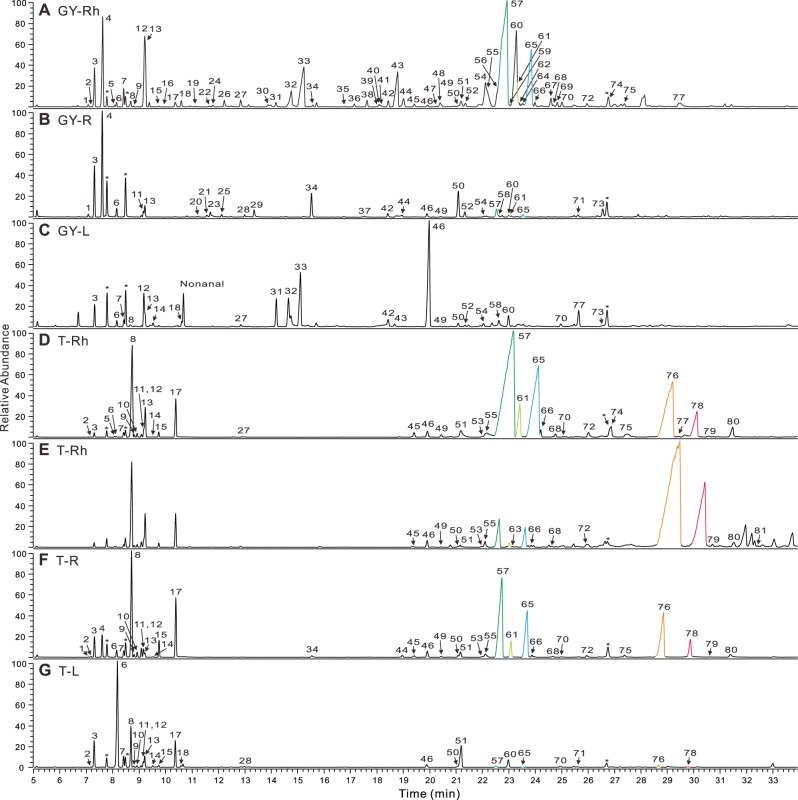
Major volatile compounds from ginger and turmeric rhizomes as analyzed by GC/MS. (**A–F**) Total ion chromatograms of 7 month old yellow ginger (GY) and turmeric (T) tissues are shown; (**A**) yellow ginger rhizome, (**B**) root, (**C**) leaf, (**D, E**) turmeric rhizome, (**F**) root and (**G**) leaf. Both ginger and turmeric produce (−)-α-zingiberene (green) and (−)-β-sesquiphellandrene (blue). However, only turmeric produces (+)-α-turmerone (orange) and (+)-β-turmerone (red). The total amounts of (+)-α-turmerone and (+)-β-turmerone are variable according to sample (**D**, **E**), but their ratios remain relatively constant. β-Bisabolene is shown as yellowish green. *: standard compounds (from early retention time to later retention time, *p*-chloro toluene, 1,2,4-trimethyl benzene and 2,4,5,6-tetrachloro-*m-*xylen). Compounds identified: 1, tricyclene; 2, α-thujene (3-thujene); 3, α-pinene; 4, camphene; 5, sabinene (4(10)-thujene); 6, β-pinene; 7, myrcene; 8, α-phellandrene; 9, 3-carene; 10, α-terpinene; 11, limonene; 12, β-phellandrene; 13, 1,8-cineole (eucalyptol); 14, (*E*)-β-ocimene; 15, γ-terpinene; 16, (*Z*)-sabinene hydrate; 17, *p*-mentha-1,4(8)-diene (terpinolene); 18, linalool; 19, (*E*)-pinene hydrate ((*E*)-pinan-2-ol); 20, α-campholenal; 21, (*E*)-pinocarveol (2(10)-pinen-3-ol); 22, camphor; 23, (*E*)-verbenol ((*E*)-2-pinen-4-ol); 24, β-citronellal; 25, pinocarvone (2(10)-pinen-3-one); 26, borneol (endo-borneol); 27, *p*-menth-1-en-8-ol (α-terpineol); 28, myrtenal; 29, verbenone (2-pinen-4-ol); 30, β-citronellol (3,7-dimethyl-6-octen-1-ol); 31, neral (β-citral); 32, (*E*)-geraniol; 33, geranial (α-citral); 34, bornyl acetate; 35, myrtenyl acetate (2-pinen-10-ol acetae); 36, δ-elemene; 37, α-terpinyl acetate; 38, citronellyl acetate; 39, nerol acetate; 40, unknown ((+)-cyclosativene-like); 41, (+)-cyclosativene; 42, α-copaene; 43, geraniol acetate; 44, β-elemene; 45, unknown (7-epi-sesquithujene-like); 46, (*E*)-caryophyllene (β-caryophyllene); 47, β-copaene; 48, γ-elemene; 49, (*E*)-α-bergamotene; 50, α-humulene (α-caryophyllene); 51, (*E*)-β-farnesene; 52, allo-aromadendrene; 53, γ-curcumene; 54, germacrene D; 55, *ar*-curcumene; 56, β-selinene (eudesma-4(14),11-diene); 57, (−)-α-zingiberene; 58, γ-amorphene; 59, α-muurolene; 60, (*E,E*)-α-farnesene; 61, β-bisabolene; 62, γ-cadinene; 63, β-curcumene; 64, 7-epi-α-selinene; 65, (−)-β-sesquiphellandrene; 66, (*E*)-γ-bisabolene; 67, α-elemol; 68, unknown (cis-sesquisabinene hydrate-like); 69, germacrene B; 70, (*E*)-nerolidol; 71, caryophyllene oxide; 72, unknown (trans-sesquisabinene hydrate-like1); 73, humulene oxide II; 74, unknown (trans-sesquisabinene hydrate-like2); 75, unknown (trans-sesquisabinene hydrate-like3); 76, (+)-α-turmerone; 77, epi-α-bisabolol/α-bisabolol; 78, (+)-β-turmerone; 79, (*Z*)-α-atlantone; 80, α-oxobisabolene; 81, (*E*)-α-atlantone.

Despite the importance of the sesquiterpenoids from these two plants to human health, the enzymes involved in their formation have not been previously identified. Terpene synthases (TPSs) are the entry point and perhaps most important type of enzymes leading to the various subclasses of terpenoids in these plants. However, only four sesquiterpene synthases (STPSs) have been characterized from the Zingiberaceae: (+)-germacrene D synthase [19] and (*S*)-β-bisabolene synthase [20] from culinary ginger (*Zingiber officinale* Rosc.) rhizome, and α-humulene synthase [21] and β-eudesmol synthase [22] from shampoo ginger (*Z. zerumbet* Smith) rhizome. These enzymes do not account for the major compounds produced in these species. For example, both ginger and turmeric produce large amounts of (−)-α-zingiberene and (−)-β-sesquiphellandrene. Turmeric also synthesizes appreciable quantities of α-turmerone and β-turmerone (Figure 1), which are also sometimes called tumerone and curlone, respectively [14], [23]. These compounds are not the direct or downstream products of the four reported TPSs. In this report, we describe the identification and characterization of a large suite of TPS enzymes involved in the formation of the large array of terpenoids found in these plants, and elucidate the means by which the sesquiterpenoids α-turmerone and β-turmerone are formed in turmeric.

## Materials and Methods

### Plant Material

Ginger (*Zingiber officinale* Rosc.) and turmeric (*Curcuma longa* L.) were grown in a greenhouse for 5 to 7 months. Two varieties of culinary ginger (white ginger and yellow ginger) were used, which are different from the white and yellow “gingers” (not *Zingiber* species at all) that have white and yellow flowers, respectively. The white and yellow gingers used in this study are culinary and medicinal varieties of ginger that have green inflorescences and morphologically are very similar to each other except that they have slightly different rhizome colors. Hawaiian red turmeric (HRT) was used for cloning genes and “fat mild orange” (FMO) turmeric was used to for GC/MS analysis, as in Figure 1. These two “varieties” are really the same clonal line that was obtained from two different organic ginger growers in Hawaii, respectively, Dean Pinner from Pinner Creek Organics and Hugh Johnson, from Puna Organics. The GC/MS total ion chromatograms of FMO and HRT are essentially identical.

### Cloning of Full Length cDNAs

Some unitrans identified in the ginger and turmeric EST databases were homologous to TPSs and appeared to be full length, although others were incomplete. For those genes missing either/both the 5′ and/or the 3′ end sequences, the SMART RACE (Rapid Amplification of cDNA End) method (Clontech) was used to find the missing 5′ or/and 3′ end(s) except for the unitrans ST00. 5′ RACE ready cDNAs and 3′ RACE ready cDNAs were synthesized from 8 different total RNAs (GW-Rh, GW-R, GW-L, GY-Rh, GY-R, GY-L, T-Rh and T-L, where GW: white ginger, GY: yellow ginger, T: turmeric, Rh: rhizome, R: root, L: leaf) extracted with the RNeasy Plant Mini kit (Qiagen) using superscript III reverse transcriptase (Invitrogen) for 3′ RACE ready cDNAs and superscript II reverse transcriptase (Invitrogen) for 5′ RACE ready cDNAs respectively. 3′ RACE CDS (AAGCAGTGGTATCAACGCAGAGTAC(T)_30_VN), 5′ RACE CDS ((T)_25_VN), and SMART II™ A Oligonucleotide (AAGCAGTGGTATCAACGCAGAGTACGCGGG) were used for RACE ready cDNA synthesis according to the manufacturer’s protocol. Amplifications of either the 3′ or 5′ end were carried out using the Advantage 2 PCR kit (Clontech) with Universal Primer A Mix (UPM, Long: CTAATACGACTCACTATAGGGCAAGCAGTGGTATCAACGCAGAGT, Short: CTAATACGACTCACTATAGGGC) and gene specific primers for RACE (Table S1). Using RACE products, a second round of PCR was done with gene specific nested primers for RACE (Table S1) and either N-UP (5′-AAGCAGTGGTATCAACGCAGAGT-3′), UP-M (5′-CACTATAGGGCAAGCAGTGGT-3′), or UP-S (5′-CTAATACGACTCACTATAGGGC-3′). PCR products of the expected estimated size were eluted using the MinElute PCR purification kit (Qiagen) and inserted into the pCR2.1-TOPO vector (Invitrogen). For ST00, we found the 5′ end by a different method, using the ZO__Ed (T-Rh) cDNA library stock. Using Zd01L13RR, a ST00 specific primer (Table S2), and UPM-T7 (5′-CTAATACGACTCACTATAGGGCGTAATACGACTCACTATAGGGCGAATTG-3′), a cloning vector specific primer, we amplified a ST00 specific fragment, which was sub-cloned as described above.

After sequencing and confirmation of 5′ and/or 3′ sequences, full length cDNAs were amplified from either 5′ RACE ready cDNA or 3′ RACE ready cDNA using Pfu thermostable polymerase with two gene-specific primer sets (Table S2) and were inserted into the pCR2.1-TOPO vector. In this manner, full length mono- and sesquiterpene synthases were cloned into the pCR2.1-TOPO vector. Truncated monoterpene synthases (with plastidial peptides removed) and full length sesquiterpene synthases were sub-cloned into expression vectors such as pCRT7/CT-TOPO vector (Invitrogen), pEXP5/CT-TOPO vector (Invitrogen), pET101/D-TOPO vector (Invitrogen) and/or pH9GW vector for expression in *E. coli*. For pH9GW-MT00, the PCR product produced using the 10N21GtwyAttF and 10N21G-AttR primers (Table S2) was first cloned using the Gateway BP reaction by Gateway BP clonase II enzyme mix (Invitrogen) with pDONR207 vector (Invitrogen) to yield pENTR207-10N21, and then the pENTR207-10N21-based Gateway LR reaction was performed using Gateway LR clonase II enzyme mix (Invitrogen) with pH9GW. For pH9GW-Zc05I02tt, the PCR product with Zc05I02tFt and Zc05I02tR primers (Table S2) was first inserted into pENTR/D-TOPO vector (Invitrogen) to produce pENTR-Zc05I02tt vector, which was used to produce pH9GW-Zc05I02tt using the Gateway LR reaction with Gateway LR clonase II enzyme mix and pH9GW. There are two versions of pCRT7CT-MT11: with His-tag or without His-tag at the 3′ end due to absence or presence of a stop codon in gene specific reverse primers, Zc07C01CT-R and Zc07C01tR, respectively (Table S2). Several genes were also cloned into the pESC-URA vector (Stratagene). PCR products amplified by appropriate primer pairs (Table S2) were sub-cloned into pCR8/GW-TOPO (Invitrogen) and fragments produced by digestion of the resulting constructs with *BamHI* (NEB) and *XmaI* (NEB) were sub-cloned into pESC-URA vector digested with *BamHI* and *XmaI*.

### Expression of Terpene Synthases in *E. coli* and Yeast

We used several *E. coli* strains for expression of terpene synthases; BL21 (DE3) pLysS (Invitrogen), BL21 CodonPlus (DE3) RIL (Stratagene), BL21 CodonPlus (DE3) RP (Stratagene), BL21 CodonPlus (DE3) RILP (Stratagene), Rosetta (Novagen), Rosetta2 (DE3) pLysS (Novagen), ArcticExpress (DE3) RIL (Stratagene), BL21-AI (Invitrogen), BL21-AI RIL, BL21 Star (DE3) (Invitrogen), BL21 Star (DE3) RIL and BL21 Star (DE3) pMevT pMBI RIL. The *E. coli* strain, BL21-AI RIL is BL21-AI plus the RIL plasmid from BL21 CodonPlus (DE3) RIL cells. The *E. coli* strain, BL21 Star (DE3) RIL is BL21 Star (DE3) plus the RIL plasmid. The *E. coli* strain, BL21 Star (DE3) pMevT pMBI RIL is BL21 Star (DE3) (Invitrogen) plus three additional plasmids, pMevT, pMBI and RIL, where pMevT and pMBI are from the Keasling lab [24] and RIL is from BL21 CodonPlus (DE3) RILP. The plasmids, pMevT and pMBI enrich IPP and DMAPP pools inside the *E. coli* cells.

*E. coli* was grown at 37°C to OD 0.6 at 600 nm and induced for 18 h at 18°C with IPTG (0.05 mM ∼ 1 mM) and 0.2% arabinose for BL21-AI derived strains.

We used several yeast strains for terpene synthase expression; INVSc1 (Invitrogen), EPY219 and EPY224, which are from the Keasling lab [25]. EPY219 and EPY224 are engineered to produce more GPP and FPP, respectively. Although EPY219 and EPY224 contain the pRS425ADS plasmid, growth in YPD media led to loss of this plasmid and EPY219 and EPY224 in this investigation did not contain the pRS425ADS plasmid. Yeast cells grown for 2 d at 30°C in SD-URA media were transferred to NB-URA media and grown at 18°C for 2–8 d.

### Enzyme Assays of Terpene Synthases Expressed in *E. coli*

Overnight grown *E. coli* cultures that had been induced to express recombinant proteins were centrifuged to collect cell pellets. The pellets were vortexed with Washing Buffer (20 mM Tris-HCl, pH 7.0, 50 mM KCl) and then centrifuged. Protein Extraction Buffer (50 mM 3-(N-morpholino)-2-hydroxypropanesulfonic acid, pH 7.0, 10% [v/v] glycerol, 5 mM MgCl_2_, 5 mM DTT, 5 mM sodium ascorbate, 0.5 mM phenylmethylsulfonyl fluoride) was added to washed *E. coli* pellets, which were then vortexed, sonicated and centrifuged. Supernatant was recovered and the buffer was changed to Enzyme Assay Buffer (10 mM 3-(N-morpholino)-2-hydroxypropanesulfonic acid, pH 7.0, 10% [v/v] glycerol, 1 mM DTT) using PD-10 columns (GE Healthcare Life Sciences). Divalent cations (20 mM MgCl_2_ and/or 0.5 mM MnCl_2_ at final concentration), phosphatase inhibitors (0.2 mM NaWO_4_, 0.1 mM NaF at final concentration) and either geranyl diphosphate (GPP, 10 µg) or farnesyl diphosphate (FPP, 10 µg) were added to total 500 µl of Enzyme Assay Buffer containing soluble proteins and incubated for 3 h at 30°C with 200 µl of top layered pentane. The top pentane phase was removed directly at the end of the assay time (or vortexed with the aqueous phase and then centrifuged prior to removal) and was used for metabolite analysis.

### Terpenoid Analysis

A Thermo Finnigan Trace GC 2000 with a Rtx-5MS w/5 m Integra-Guard Column (Restek, 0.25 mm ID, 0.25 µm df, 30 m) coupled to a DSQ mass spectrometer was used for gas chromatography/mass spectrometry (GC/MS) analysis, using methods previously described [26]. A chiral column, Rt-βDEXse (Restek, 0.25 mm ID, 0.25 µm df, 30 m), was used for determining enantiomers of linalool and caryolan-1-ol. Eluted compounds were identified by comparison of resulting mass spectra to the NIST/EPA/NIH Mass Spectral Library (NIST 02) and the essential oil GC/MS mass spectra library from Dr. Robert P. Adams [27]. For peak identification, we used both mass spectra similarity and peak retention time indices unless we specify the use of authentic standards. Adams’ essential oil library has a retention time index. An example of how the retention time index was used with mass spectra similarity for peak identification is shown in Figure S1. Unless an authentic standard was used, all identifications should be viewed as tentative, although we believe that for the important compounds discussed in this manuscript, they are indeed correct.

### Western Blot Analysis for Terpene Synthases Expressed in Yeast

Proteins from yeast expressing terpene synthases were extracted using acid-washed glass beads (425–600 µm, 30–40 U.S. sieve) (Sigma). Yeast cell pellets from 10 to 15 ml of media were vortexed 15 times for 30 seconds on ice with 0.5 g of acid-washed glass beads and Protein Extraction Buffer (see above). Protein concentrations were determined by the Bradford Protein Assay (Bio-Rad). After 10 µg of total and soluble proteins were run on SDS-PAGE, the gel was blotted onto PVDF Transfer Membrane (0.45 µm, Thermo Pierce) with transfer buffer (12 mM Tris, 96 mM Glycine, 20% (v/v) MtOH, pH 8.3) in a western blot transfer apparatus according to the protocol of the PVDF Transfer Membrane manual. After treatment with blocking solution (TBS containing 5% [w/v] non-fat milk powder), membranes were incubated with blocking solution containing Mouse Anti-c-Myc-tag Monoclonal Antibody (Genscript) for 2 h, washed twice with TBS-T (TBS containing 0.1% (v/v) Tween-20) for 30 min, incubated with blocking solution containing Goat Anti-Mouse IgG (H&L) [HRP] Polyclonal Antibody (Genscript) for 2 h and washed twice with TBS-T for 30 min. The ECL system (SuperSignal West Pico Chemiluminescent Substrate, Thermo Pierce) was used to detect expressed terpene synthases in the yeast. For positive control we used the Multiple Tag (Purified) (Genscript).

### Protein Structural Modeling

SWISS-MODEL [28] was used to model the putative protein structures and UCSF Chimera [28] was used to visualize the models.

## Results

### Cloning and Expression of Ginger and Turmeric TPSs

In efforts to identify how the large array of mono- and sesquiterpenoids (Figure 1) in ginger and turmeric are produced, we first searched a database of 50,139 expressed sequence tags (ESTs) from ginger and turmeric tissues (http://www.agcol.arizona.edu/cgi-bin/pave/GT/index.cgi), and identified many putative terpene synthases (TPSs) that would be expected to catalyze the formation of monoterpenoids (20 unique transcripts [unitrans]), sesquiterpenoids (10 unitrans), diterpenoids (2 unitrans), triterpenoids (3 unitrans), and tetraterpenoids (10 unitrans). Two monoterpene synthases (MTPSs) and no sesquiterpene synthase (STPS) were represented by full length sequences in the database. The rest of the identified TPS genes either required RACE or Genome Walking followed by RT-PCR to obtain full length cDNAs or did not yield full length clones after these efforts (Table S3). During the cloning process, we sequenced several independent clones for each unitrans and found that some exist as multiple paralogs and/or alleles in these species. In some cases, only the paralog(s) could be cloned, whereas the gene represented by the sequence in the original database could not. Because we often found more than two sequences during the RT-PCR-based cloning, we are confident that most of these are indeed paralogs, and not merely allelic pairs. Turmeric is sterile and likely a nonaploid (*x* = 7, 2*n* = 9*x* = 63) [29], thus providing an explanation for why so many related sequences could be found in one species.

**Figure 2:**
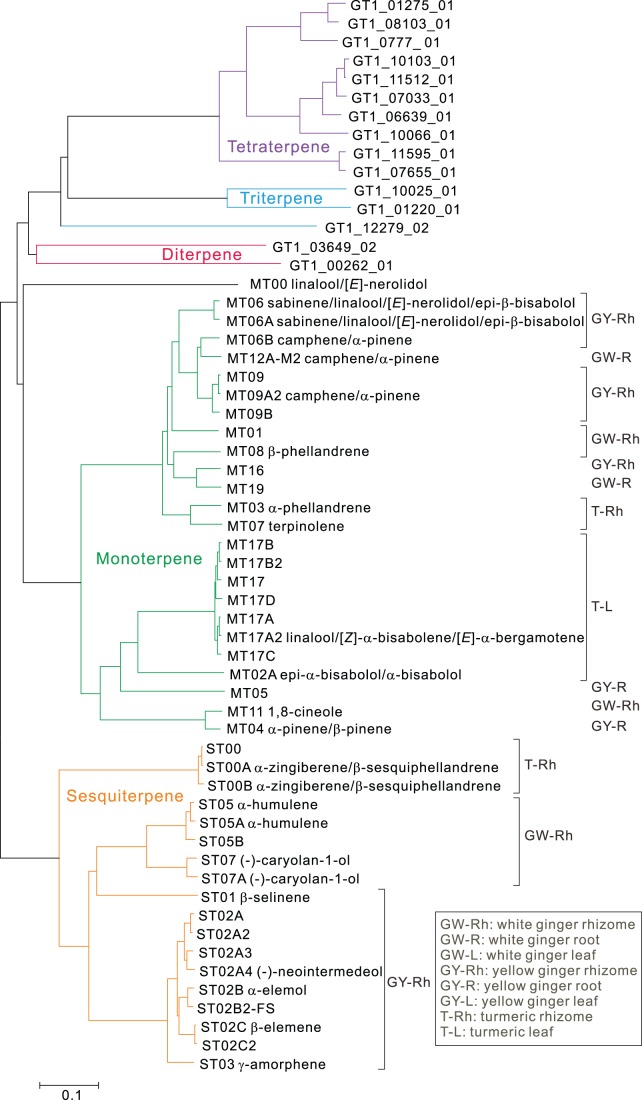
Similarity tree of cloned full length mono- and sesquiterpene synthases and unitrans sequences of di-, tri- and tetra-terpene synthases from ginger and turmeric. The neighbor-joining tree was generated by ClustalX with ginger and turmeric sequences and 181 additional TPSs from GenBank. The non-ginger/turmeric TPSs were removed from the tree for clarity. The STPS cluster is separate from other terpene synthases. Linalool/nerolidol synthase (MT00) is located outside of the MTPS cluster. Major product(s) of each corresponding recombinant protein is/are shown next to each gene name, as is the tissue used for cloning.

A similarity tree generated from a large set of plant TPSs and ginger and turmeric TPS genes including full length genes has one MTPS cluster and one STPS cluster (Figure 2). One MTPS, called MT00, is a linalool/nerolidol synthase and the outgroup relative to other MTSs. All of the ginger and turmeric TPS proteins possess the conserved DDXXD motif required for interaction with the diphosphate group of the substrate (Figure S2). All MTPSs, except for MT00, have a transit peptide and the conserved RRX_8_W motif in the 5′ region. Most STPSs have the RX_9_W motif except for ST01, β-selinene synthase. Although MT00 does not possess the conserved tryptophan of the RRX_8_W motif, ST01 does.

The major products produced by ginger and turmeric TPSs are summarized in Table 1 and 2. When provided the alternative substrate in vitro, some MTPSs produce sesquiterpenes whereas some STPSs synthesize monoterpenes. However, based on the results outlined below, it is likely that such functions have little or no relevance to production of these compounds in vivo in most cases. Although several of the proteins expressed well in *E. coli*, many required expression in yeast in order to produce soluble and functional enzymes. More detailed descriptions of expression and analysis of many specific TPS genes is included in Results S1.

**Table 1:** Monoterpenes produced by ginger and turmeric terpene synthases.

	MT00	MT03	MT04	MT06/ MT06A	MT06B	MT07	MT08	MT09A2	MT11	MT12A-M2	MT17A2	ST00A/ ST00B	ST02B	ST02C	ST03
tricyclene					0.7			1.5		1.2					
α-thujene (3-thujene)				3.4											
α-pinene			**60.1**		**40.4**		11.7	20.4	4.4	28.4		33.7			
camphene					**45.4**			**60.1**		**55.8**					
sabinene (4(10)-thujene)			2.7	29.0								6.2			
β-pinene			30.7		1.0			0.6	3.7	0.7		5.9			
myrcene													**25.0**	**30.4**	**27.3**
α-phellandrene		**92.2**				2.7						5.0			
3-carene						1.2									
α-terpinene		1.0		8.2		4.3									
limonene			5.6		6.6	1.5		7.7		7.9			15.5	17.9	2.7
β-phellandrene		3.8					**88.**3					**49.2**			
1,8-cineole (eucalyptol)			0.9						**75.4**						
(*Z*)-β-ocimene													14.3	13.5	17.6
(*E*)-β-ocimene													9.8	9.3	3.5
γ-terpinene		2.5		9.0		0.4		0.1							
cis-sabinene hydrate				6.2	0.4			0.4		0.1					
*p*-mentha-1,4(8)-diene (terpinolene)		0.5		5.2	1.5	**89.9**		1.6		1.9			7.7	9.9	5.2
linalool	**100**			24.8							**100**		**25.1**	15.6	**36.8**
cis-*p*-menth-2-en-1-ol															3.8
trans-pinene hydrate (trans-pinan-2-ol)					0.3			0.3		0.3					
β-citronellal								0.9							
borneol (endo-borneol)					3.3			6.1		3.3					
*p*-menth-1-en-4-ol (terpinen-4-ol)				8.0											
*p*-menth-1-en-8-ol (α-terpineol)				6.2	0.3			0.4	16.5	0.4			2.7	3.3	3.3

Numbers represent percentage of total products for each terpene synthase. Note: Bold and underlined numbers indicate the most abundant compound(s) produced by specific TPS proteins. Limonene from MT04 also contains a (*R*)-(+)-*m*-mentha-6,8-diene (sylvestrene)-like compound (see text and Figure S9).

**Table 2:** Sesquiterpenes produced by ginger and turmeric terpene synthases.

	MT00	MT02A	MT06/ MT06A	MT08	MT17A2	ST00A/ ST00B	ST01	ST02A4	ST02B	ST02C	ST03	ST05	ST05A	ST07	ST07A
δ-elemene								1.2		2.9					
unknown ((+)-cyclosativene-like)									7.8	2.1					
(+)-cyclosativene										3.2					
α-copaene									11.2	1.7					
unknown (β-elemene-like)										2.1					
β-elemene							9.7	12.6	18.3	**49.3**		1.5	1		
unknown (7-epi-sesquithujene-like)			8.1		0.8	0.3									
cis-α-bergamotene					7.6										
(*E*)-caryophyllene (β-caryophyllene)								0.5		2.4		14.2	10.5	1.3	0.2
trans-α-bergamotene		0.1			**20.6**	0.2									
γ-elemene								1.1	6.1	2.7					
unknown (guaia-1(5),7(11)-diene-like)							3.4								
(*Z*)-β-farnesene				**100**											
α-humulene (α-caryophyllene)												**83.4**	**88.4**		0.4
allo-aromadendrene											11.8				
unknown (ererrophila-1(10),11-diene-like)							11.3								
γ-muurolene										4.7					
unknown (β-chamigrene-like)							7.4								
*ar*-curcumene						0.9									
γ-curcumene			4.4		1.6	0.3									
unknown (β-sesquiphellandrene-like)					1.5										
germacrene D								6.1	1.6	12.4	4.4				
β-selinene (eudesma-4(14),11-diene)							**51.9**								
(−)-α-zingiberene						**67**									
γ-amorphene											**65.4**				
α-muurolene								1.6	3.1	9.5					
β-bisabolene		1.1			13.6	6.2									
(*Z*)-α-bisabolene		1.7			**22.7**										
γ-cadinene											8.7				
β-curcumene			14.5												
7-epi-α-selinene							14.2								
(−)-β-sesquiphellandrene			1.9		3.7	**22.7**									
δ-cadinene (cadina-1(10),4-diene)								1.2	2.8	4					
(*E*)-γ-bisabolene			0.6			0.4									
unknown (cis-α-bisabolene-like)					2.1										
1,5,9-trimethyl-1,5,9-cyclododecatriene												0.8			
unknown (cis-sesquisabinene hydrate-like)			4.5												
α-elemol									**44.3**						
germacrene B									4.9	2.9					
(*E*)-nerolidol	**100**		41		5.8										
(−)-caryolan-1-ol (β-caryophyllene alcohol)														**98.7**	**99.5**
germacrene D-4-ol											9.6				
unknown (trans-sesquisabinene hydrate-like1)			1.2												
unknown (trans-sesquisabinene hydrate-like2)						0.6									
unknown (selina-6-en-4-ol-like)								2.4							
unknown (cubenol-like)								6.5							
unknown (spathulenol-like)								0.4							
unknown (α-eudesmol-like)						0.3									
γ-eudesmol						0.4		0.8							
unknown (trans-sesquisabinene hydrate-like3)						0.4									
α-acorenol						0.2									
epi-α-muurolol (τ-muurolol)								6							
α-muurolol (δ-cadinol)								2.5							
α-cadinol								7.5							
(−)-neointermedeol							2.1	**48.7**							
(+)-intermedeol								0.8							
epi-β-bisabolol			**22.3**												
epi-α-bisabolol		**58.3**	1.6		12.6										
α-bisabolol		38.7			7.3										

Numbers represent percentage of total products for each terpene synthase. Note: Bold and underlined numbers indicate the most abundant compound(s) produced by specific TPSs.

### Function of TPSs Explain Formation of Major Terpenoids in Ginger and Turmeric

**Figure 3:**
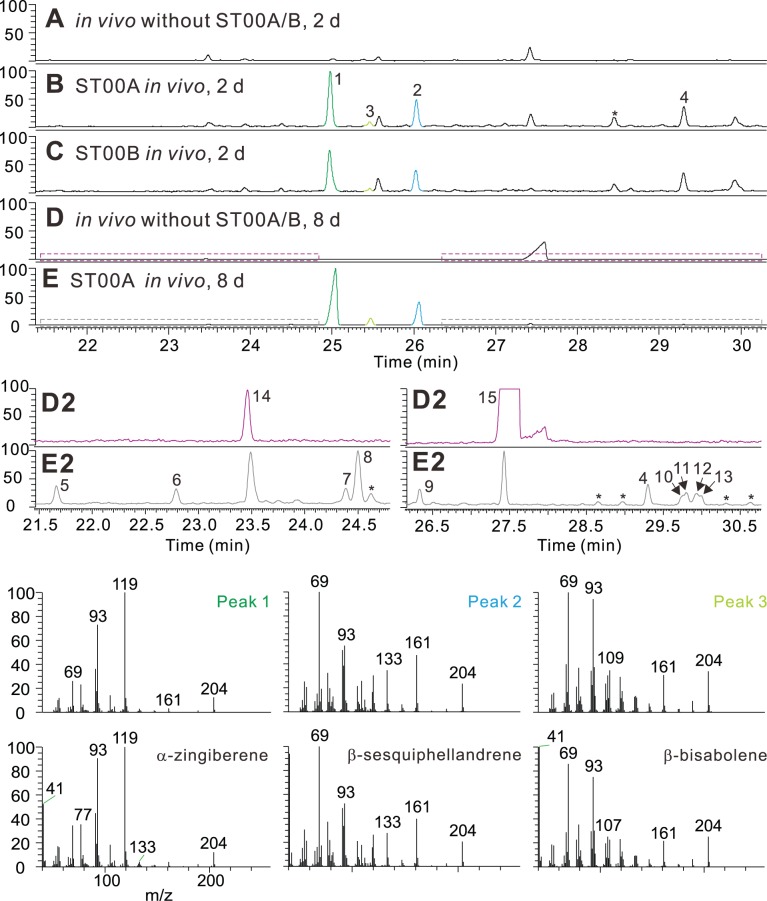
Analysis of ST00A and ST00B functions when proteins were expressed in the yeast strain, EPY219. (**A–E**) Total ion chromatograms are displayed: (**A**) EPY219 without pESC-URA-ST00A or pESC-URA-ST00B, 18°C, 2 days; (**B**) EPY219 expressing ST00A, 18°C, 2 days; (**C**) EPY219 expressing ST00B, 18°C, 2 days; (**D**) EPY219 without pESC-URA-ST00A or pESC-URA-ST00B, 18°C, 8 days; and (**E**) EPY219 expressing ST00A, 18°C, 4 days. **D2** and **E2** are boxed regions of **D** and **E** panels to show very small peaks (expanded y-axes). Additional mass spectra comparisons are in Figure S4. Products/compounds identified: 1, (−)-α-zingiberene; 2, (−)-β-sesquiphellandrene; 3, β-bisabolene; 4, unknown (trans-sesquisabinene hydrate-like2); 5, unknown (7-epi-sesquithujene-like); 6, trans-α-bergamotene; 7, γ-curcumene; 8, *ar*-curcumene; 9, (*E*)-γ-bisabolene; 10, unknown (α-eudesmol-like); 11, γ-eudesmol; 12, unknown (trans-sesquisabinene hydrate-like3); 13, α-acorenol; 14, (*E*)-β-farnesene; 15, (*E*)-nerolidol.

As seen in Figure 1, the most abundant terpenes in ginger and turmeric are (−)-α-zingiberene and (−)-β-sesquiphellandrene, based on GC/MS peak areas in total ion chromatograms of extracts from these plants. Two very similar TPS proteins, ST00A and ST00B with 98.4% similarity to each other and high similarity to known STPSs, and lacking a chloroplastic transit peptide, were cloned from turmeric rhizome. These expressed well in *E. coli* and synthesized the same products in vitro when supplied FPP as substrate: (−)-α-zingiberene (49.3%), (−)-β-sesquiphellandrene (40.7%) and β-bisabolene (6.3%) as major products (Figure S2). When expressed in the yeast strain EPY219, which supplies endogenous prenyl diphosphate precursors, GPP and FPP for production of terpenoids in vivo, these proteins produced the same major products, although the ratios of the products differed according to expression temperature and induction time, e.g., (−)-α-zingiberene (67%), (−)-β-sesquiphellandrene (22.7%), and β-bisabolene (6.2%) after 4 days of induction at 18°C (Figure 3; Figure S3). When expressed in other yeast strains or under different expression temperatures and induction times, the ratios of these products varied, as did the amounts of other minor products of these enzymes. The other STPS genes identified in our database did not produce (−)-α-zingiberene or (−)-β-sesquiphellandrene when expressed in *E. coli* or in various yeast strains. Thus, it appears that these two enzymes and their paralogs are responsible for formation of the most abundant terpenes in the rhizomes of these plants. As outlined in a later section, these compounds appear to be the precursors for the major oxygenated terpenoids in turmeric as well, indicating a major role for these enzymes in determining that plant’s terpenoid profile. A recently reported terpene synthase from sorghum, SbTPS1 also produces (−)-α-zingiberene and (−)-β-sesquiphellandrene as major products in very similar ratios to ST00A/B, although SbTPS1 is only 43% identical with ST00A [30], [31].

β-Phellandrene, the major product of the protein designated MT08, is the second most abundant monoterpene in ginger rhizomes (Figure 1). This protein, a MTPS with a chloroplastic targeting peptide, was very difficult to express in soluble form. However, when finally expressed and assayed, it produced both β-phellandrene (88.3%) and α-pinene (11.7%) (Table 1, Figure S5). No other MTPS produced β-phellandrene as a major product. (−)-β-Phellandrene and (−)-β-sesquiphellandrene are structurally identical, except that the latter has a longer tail. β-Phellandrene can also be produced by the two α-zingiberene/β-sesquiphellandrene synthases (ST00A and ST00B) described above, which make β-phellandrene as 49.2% of their monoterpene production, when provided GPP as substrate. However, GPP is a very poor substrate for these enzymes in general. When considering the fact that monoterpenes are mainly produced in plastids, and ST00A and ST00B are not targeted to that organelle, whereas MT08 is, the latter is likely to be the main enzyme responsible for formation of β-phellandrene in ginger rhizome. Microarray data also shows high expression of MT13 ( = MT08) in ginger rhizome over time (Table S4).

Camphene, the most abundant monoterpene in ginger, was produced by three different enzymes, MT06B, MT09A2 and MT12A-M2, which all function as camphene/α-pinene synthases and produce camphene as the major product when provided with GPP as substrate (Table 1, Figures S6, S7 and S8) but were not able to use FPP as a substrate. In contrast, the enzyme MT04 produces no camphene, but produces α-pinene (60.1%) and β-pinene (30.7%) as major products (Table 1, Figure S9). It is likely that most of the α-pinene accumulated by ginger rhizomes is produced by the camphene/α-pinene synthases (MT06B, MT09A2 and MT12A-M2). The β-pinene peak from ginger rhizome samples is very small (Figure 1). Moreover, the amount of α-pinene produced by MT04 is only about twice that of β-pinene, which suggests that MT04 contributes at most ∼21% of the α-pinene produced in ginger rhizomes. In contrast, ginger roots accumulate both α-pinene and β-pinene at significantly higher levels compared to camphene (Figure 1). Differences between extracts from plants of different ages supported this observation, where MT04 products (α-pinene and β-pinene) are more abundant in roots of 2 month old yellow ginger plants when compared to 7 month old plants; 5 times more for β-pinene. These results were supported by microarray results for tissues from these plants (with 7-fold higher expression in the younger plants, for MT04; Table S4). It thus appears that MT04 synthesizes most of the β-pinene found in ginger rhizomes and roots, whereas α-pinene accumulation is the result of the action of several enzymes. β-pinene is also the main monoterpene in turmeric leaves, but MT04 was not expressed at all in turmeric, suggesting that turmeric possesses an as yet unidentified leaf-specific β-pinene synthase.

Both ginger and turmeric rhizomes produce 1,8-cineole in large amount (Figure 1) and recombinant MT11 makes 1,8-cineole (75.4%), *p*-menth-1-en-8-ol (α-terpineol) (16.5%), α-pinene (4.4%) and β-pinene (3.7%) with GPP as substrate (Table 1, Figure S10). This enzyme could not use FPP. The relative amounts of 1,8-cineole and *p*-menth-1-en-8-ol (α-terpineol) in yellow ginger rhizome (Figure 1) are 77.57% and 22.42% respectively, which is similar to but not identical with the MT11 product ratio. *p*-Menth-1-en-8-ol (α-terpineol) is also produced by MT06/MT06A (6.2%, described below), which may contribute to its production in vivo. According to the microarray data, the expression level of MT11 is high in ginger rhizome, but 29 and 32 times less abundant in ginger root and leaf, respectively, and not expressed at all in turmeric (Table S4). These results confirm MT11 as the likely major contributor to 1,8-cineole synthesis in ginger rhizome, but indicate that there must be a separate turmeric-specific 1,8-cineole synthase that we did not identify.

α-Phellandrene is the most abundant monoterpene in turmeric rhizome (Figure 1). Although ginger contains α-phellandrene, it is not a major product. The enzyme designated MT03 synthesizes α-phellandrene (92.2%) as a major product, with β-phellandrene (3.8%) and several other compounds as minor products (Table 1, Figure S11). FPP was not a substrate for MT03. Microarray data suggest high expression of MT10 ( = MT03, see Table S3) at early stages of turmeric rhizome development, with a reduction in expression over time (Table S4). The levels of α-phellandrene also decrease over development in turmeric rhizome, although this decrease was not as great in magnitude as that observed for the transcript level. It is possible that turmeric rhizome stores α-phellandrene in the rhizome, leading to accumulation in older rhizomes. The expression levels of MT03 in turmeric root and leaf are lower than that observed for turmeric rhizome, and α-phellandrene amounts in root and leaf of 7 month old turmeric plants are 28.7% and 14.5% of that observed for the rhizome of the same plants. Thus, it appears that MT03 is likely the major producer of α-phellandrene in turmeric.

Turmeric rhizome produces *p*-mentha-1,4(8)-diene (terpinolene) as its second most abundant monoterpene (Figure 1). With GPP as a substrate, the enzyme designated MT07 synthesized *p*-mentha-1,4(8)-diene (terpinolene) (89.9%) as a major (Table 1, Figure S12). MT07 did not utilize FPP as substrate. MT07 is not expressed in ginger. Based on microarray data, it is expressed in turmeric rhizome (Table S4). In ginger, only the rhizomes possessed detectable amounts of *p*-mentha-1,4(8)-diene (Figure 1), which is probably produced by MT06/MT06A. The production of *p*-mentha-1,4(8)-diene (terpinolene) in turmeric root and leaf (Figure 1) and the lack of MT07 expression in turmeric root and leaf suggests there could be other *p*-mentha-1,4(8)-diene (terpinolene) synthases in those tissues.

**Figure 4:**
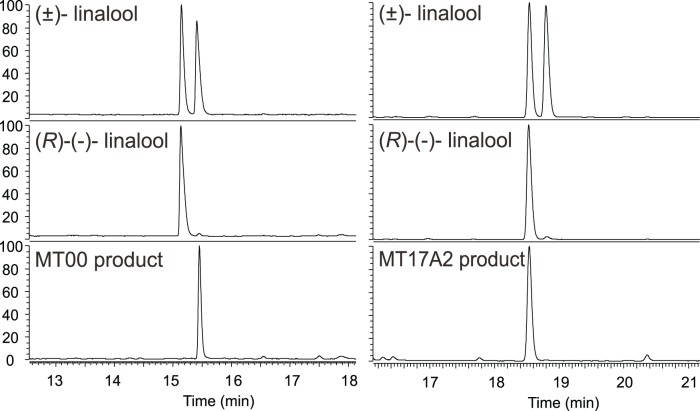
Analysis of linalools produced by MT00 and MT17A2 in chiral column. MT00 produces (*S*)-(+)-linalool and MT17A2 synthesizes (*R*)-(−)-linalool.

Ginger rhizome and leaf and turmeric leaf produce small amounts of linalool (Figure 1). Two MTPSs, MT00 and MT17 from turmeric leaf, produce linalool from GPP. The best hit from a BLAST search of MT00 against public sequence databases is to another linalool synthase ((3S)-linalool/(*E*)-nerolidol synthase [*Vitis vinifera*] [32]). Enzyme assays with recombinant MT00 produced linalool (100%) with GPP as a substrate and (*E*)-nerolidol (100%) with FPP as a substrate (Table 1 and 2, Figure S13). MT17, in contrast, is more similar to other monoterpene synthases than to other known linalool synthases. Efforts to clone a full length cDNA for MT17 yielded diverse paralogs: MT17A, MT17A2, MT17B, MT17B2, MT17C and MT17D (Figure 2). Enzyme assays with MT17A2 synthesized linalool (100%) with GPP and a variety of sesquiterpenes with FPP as substrate (Table 1 and 2, Figure S14), with cis-α-bisabolene (22.7%), trans-α-bergamotene (20.6%), β-bisabolene (13.6%), epi-α-bisabolol (12.6%) as the major products. When a chiral column was used to analyze the products of these assays, we found that MT00 produces (*S*)-(+)-linalool while MT17A2 synthesizes (*R*)-(−)-linalool (Figure 4). Thus, these are indeed quite different enzymes, yielding different products, which are both unfortunately trivially called “linalool”. The evolution of MT00 is likely to have followed the same course as the *Vitis vinifera S*-linalool synthase, whereas MT17 is like other known *R*-linalool synthases, which have evolved very recently in their respective plant species from other MTPSs.

MT00 and MT17 are not expressed in ginger tissues. Instead, MT06/MT06A, which produces 24.8% linalool, is likely to be the enzyme responsible for formation of *-*linalool in ginger. MT06 and MT06A are 98.0% identical, the only differences are found in the transit peptide sequences (Figure S2), and thus appear to be paralogs. MT06 and MT06A produce a variety of monoterpenoids and sesquiterpenoids (Table 1 and 2, Figures S15 and S1). Several MT06/MT06A products, such as α-thujene (3-thujene), *p*-menth-1-en-4-ol (terpinen-4-ol), β-curcumene, epi-β-bisabolol and some unknown compounds, were only produced by MT06/MT06A among the recombinant proteins that we characterized. Although it is possible that there are other yet unidentified enzymes that specifically synthesize these products, these are minor products in ginger and turmeric tissues and it is likely that they are produced by the paralogs MT06/MT06A.

There are small amounts of epi-α-bisabolol and α-bisabolol in ginger and turmeric rhizomes. MT02A was difficult to express in soluble form, but produced the sesquiterpenoids epi-α-bisabolol (58.3%) and α-bisabolol (38.7%) as major products when expressed in yeast strain EPY224. MT02A also synthesized trace amounts of (*Z*)-α-bisabolene (1.7%), β-bisabolene (1.1%), and *trans*-α -bergamotene (0.1%) (Figure S16). Based on sequence similarity, MT02A is classified as a monoterpene synthase, however it did not produce monoterpenes in yeast. This may be due to the properties of the enzyme or because yeast may have limited ability to produce monoterpenes [33], [34].

The sesquiterpene β-selinene (eudesma-4(14),11-diene) is produced at detectable levels in ginger rhizome, but is found only at trace amounts in ginger root and is not detectable in ginger leaf or turmeric tissues. Recombinant ST01 synthesized β-selinene (eudesma-4(14),11-diene) (51.9%) as the major product when FPP was used as substrate (Table 2, Figure S17). With GPP as a substrate, ST01 did not produce any detectable product. ST01 is expressed at higher levels in ginger rhizome than ginger root and leaf and is not expressed in turmeric, according to microarray data (Table S4), supporting the role of this enzyme in production of β-selinene in vivo.

**Figure 5:**
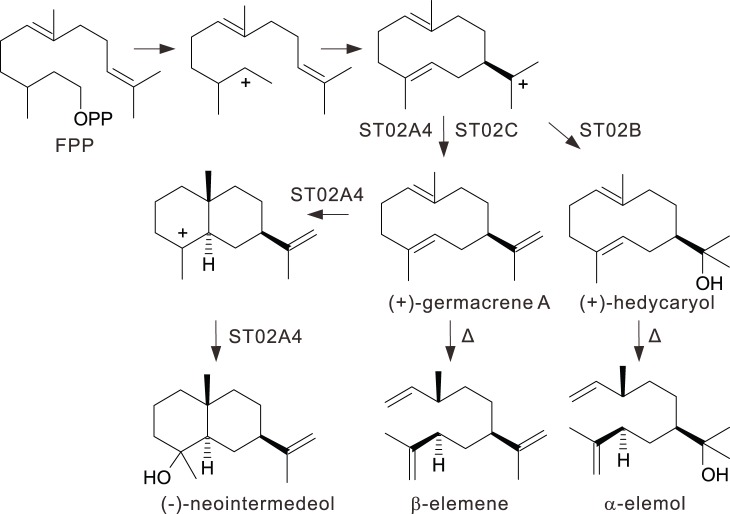
Proposed synthesis of (−)-neointermedeol, α-elemol and β-elemene from FPP. These are major products of ST02A4, ST02B and ST02C respectively and share the same mechanistic pathway. Δ: heat during GC run.

None of the STPS genes were represented by complete sequences in the EST database, requiring additional efforts such as RACE or genome walking to yield full length cDNA sequences. During the cloning of one such gene, designated ST02, many paralogs were found, ST02A, ST02A2, ST02A3, ST02A4, ST02B, ST02B2-FS, ST02C and ST02C2, which had similar yet distinct product profiles. Although many of the corresponding recombinant proteins for these genes were insoluble, some were soluble or partially soluble in *E. coli* or yeast. Yeast strain EPY224 expressing ST02A4 produced (−)-neointermedeol (48.7%) as a major product and a long list of minor products (Table 2, Figure S18). Enzyme assays using *E. coli* crude extract expressing ST02B with GPP as a substrate produced several monoterpenes, with linalool (25.1%), myrcene (25.0%), limonene (15.5%) as major products (Table 1, Figure S19). With FPP as a substrate, ST02B also produced several sesquiterpenes, with α-elemol (44.3%) as the major product (Figure S20). Enzyme assays using *E. coli* crude extracts expressing ST02C with GPP as a substrate produced several monoterpenes, with myrcene (30.4%), limonene (17.9%), linalool (15.6%) as major products (Table 1, Figure S21). Similar assays for ST02C with FPP as a substrate produced β-elemene (49.3%) and germacrene D (12.4%) as major products and a long list of minor products (Table 2, Figure S22). It is likely that the β-elemene detected in these assays is a thermal degradation product of germacrene A [35]–[36] (Figure 5), because our GC/MS inlet was initially set to a high temperature (220°C). Reducing the GC inlet temperature (to 150°C), as has been suggested [37], led to a decrease in the amount of β-elemene detected with an increase in germacrene A detected in our system. Although the amount of germacrene A was still small and the reduction of β-elemene not so dramatic, it is likely that the β-elemene that we detected may indeed result from thermal degradation of germacrene A. Similarly, α-elemol is likely the thermal breakdown product of (+)-hedycaryol (Figure 5). However the low inlet temperature led to greatly reduced sensitivity, so the relative product amounts shown in Table 2 are based on injection at 220°C.

Based on the microarray data, ST02 was expressed 8-, 120- and 36-fold higher in ginger rhizome compared to ginger root, ginger leaf and turmeric leaf, respectively. The microarray data indicated lack of expression of ST02 in turmeric rhizome and root (Table S4). Based on ginger and turmeric terpene synthase product profiles, several terpenes were exclusively produced by ST02A4, ST02B or ST02C, including δ-elemene, (+)-cyclosativene, α-copaene, γ-elemene, γ-muurolene, α-muurolene, δ-cadinene (cadina-1(10),4-diene), α-elemol, germacrene B although some are produced at very low levels. Among these compounds, γ-muurolene and δ-cadinene (cadina-1(10),4-diene) were not detected from ginger or turmeric samples, α-copaene was detected in both ginger rhizome and root samples, and all others are only detected in ginger rhizome samples (Figure 1). When these results are considered together, it appears that diverse ST02 paralogs play an important role in enriching sesquiterpene diversity in ginger rhizome.

The sesquiterpene synthase designated ST03 is very similar (89%–95% identical) to ST02 derived genes. However, recombinantly expressed ST03 synthesizes different products compared to the ST02 enzymes. Enzyme assays with FPP as substrate and using crude *E. coli* extract containing expressed ST03 yielded γ-amorphene (65.4%) as the major product, with allo-aromadendrene (11.8%), germacrene D-4-ol (9.6%), γ-cadinene (8.7%) and germacrene D (4.4%) also produced at appreciable levels (Table 2, Figure S23). Comparable assays with GPP as substrate yielded a number of monoterpenoids (Table 1, Figure S24), although this enzyme is not likely transported to the plastid. The major sesquiterpene product of this enzyme, γ-amorphene, is present at low levels in ginger, and is barely detectable in turmeric (Figure 1). It is difficult to detect γ-amorphene in extracts from ginger rhizome (the tissue where the ST03 gene is most highly expressed) because it comes off of the GC column immediately after the very large (−)-α-zingiberene peak. Two products of ST03, allo-aromadendrene and γ-cadinene, are more readily detected, with allo-aromadendrene present in ginger rhizome, root and leaf and γ-cadinene in ginger rhizome. ST03, which is expressed in ginger rhizome, root and leaf based on microarray data (Table S4), is the likely source of these compounds. And as expected based on the chemical profile, ST03 was not expressed in turmeric according to microarray data.

α-Humulene (also called α-caryophyllene) is present in all turmeric tissues and in ginger rhizome at very low levels. However, it is the most abundant sesquiterpene in ginger roots. ST05 (and close paralog ST05A), which produces α-humulene in vitro, was expressed at much higher levels in ginger root than in other tissues (Table S4). ST05 and ST05A are very similar paralogs: 99.1% and 98.1% identity at the DNA and amino acid sequence levels, respectively. Although they are very similar, their solubilities are different. ST05A is barely soluble and ST05 is quite soluble (Table S5). Therefore, we tried to purify ST05A using a HIS-tag. However, the amount of soluble ST05A was small, and some *E. coli* proteins co-eluted, leading only to partial purification. Enzyme assays with crude *E. coli* extracts containing expressed recombinant ST05 or with partially purified ST05A produced very similar results. These assays did not produce detectable monoterpenes with GPP as a substrate. With FPP as a substrate, however, ST05 synthesized α-humulene (α-caryophyllene) (83.4%) as the major product and (*E*)-caryophyllene (β-caryophyllene) (14.2%), β-elemene (1.5%) and 1,5,9-trimethyl-1,5,9-cyclododecatriene (0.8%) as minor products (Table 2, Figure S25). ST05A showed a similar product profile, except for the last compound, which was not detected. An α-humulene synthase from shampoo ginger (*Zingiber zerumbet* Smith) has 91% similarity to ST05 and ST05A. That enzyme was reported to produce α-humulene (α-caryophyllene) as major product and small amounts of (*E*)-caryophyllene (β-caryophyllene) [21]. However, shampoo ginger α-humulene synthase did not synthesize β-elemene or 1,5,9-trimethyl-1,5,9-cyclododecatriene. It is possible that these compounds were not detected due to very low abundance, just as we could not detect 1,5,9-trimethyl-1,5,9-cyclododecatriene in our ST05A assays. Interestingly, (*E*)-caryophyllene is the most abundant sesquiterpene in ginger leaf but there are only small amounts of α-humulene in ginger leaves. This suggests that either α-humulene is converted in ginger leaves into another compound (which we could not detect in our metabolite profiling experiments) or ginger leaves have a different terpene synthase that produces (*E*)-caryophyllene as a major product.

**Figure 6:**
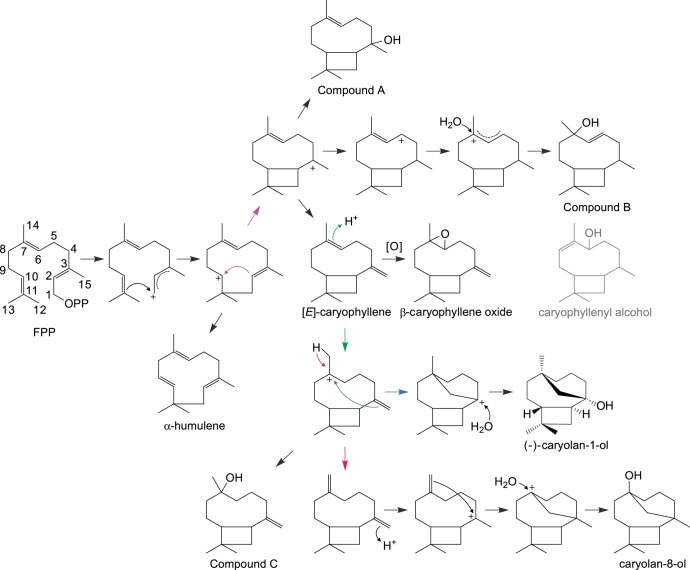
Proposed mechanism for the formation of caryophyllene related compounds, the proposed major products of ST07/ST07A. The mass spectrum of the major product of ST07A is very similar to caryophyllenyl alcohol, caryolan-1-ol and caryolan-8-ol. However, it seems unlikely that caryophyllenyl alcohol would be produced by a single terpene synthase enzyme.

A different pair of parologous STPSs, ST07 and ST07A (97% identical to each other), is not expressed in turmeric, but is expressed at high levels in ginger root and leaf tissues (Table S4). These expression patterns are similar to (*E*)-caryophyllene production patterns in ginger and turmeric tissues, suggesting that ST07 and ST07A might be good candidates for involvement in production of (*E*)-caryophyllene. These proteins were essentially insoluble in *E. coli* expression systems (several were tested, see Table S5). Despite their high sequence similarity, ST07A expressed in yeast is highly soluble, whereas ST07 expressed in yeast was much less soluble when checked by Western blotting. Based on GC/MS library searches, the best hits for the major product of yeast-expressed ST07 and ST07A (see Figures S26 and S27) are caryophyllenyl alcohol, caryolan-1-ol or caryolan-8-ol, all of which are oxygenated forms of (*E*)-caryophyllene. It seems unlikely that caryophyllenyl alcohol could be produced directly by a terpene synthase, whereas caryolan-1-ol, caryolan-8-ol or other oxygenated forms of (*E*)-caryophyllene (Compound A, Compound B and Compound C shown in Figure 6) can be made by a terpene synthase. We could not find oxygenated (*E*)-caryophyllene derivatives in ginger and turmeric except for caryophyllene oxide. Therefore, it seemed possible that ST07 and ST07A may produce (*E*)-caryophyllene, which is observed in all tissues of both ginger and turmeric, and that (*E*)-caryophyllene produced in yeast would then be oxygenated by yeast proteins in the expression system used. However, (*E*)-caryophyllene was produced in yeast without signs of being oxygenated [36]. Therefore, ST07 and ST07A do indeed appear to produce oxygenated forms of (*E*)-caryophyllene. In ginger, the product of ST07 and ST07A, an oxygenated (*E*)-caryophyllene, could be further processed by other enzymes and would therefore not be detected in our GC/MS analysis shown in Figure 1.

**Figure 7:**
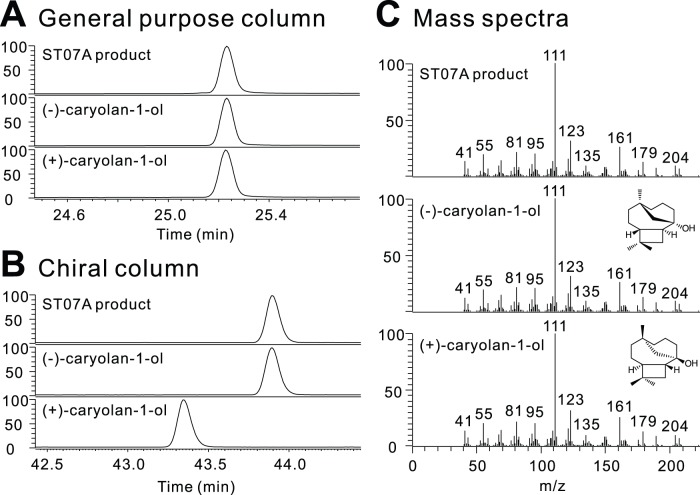
Comparison of ST07A major product to (−)-caryolan-1-ol and (+)-caryolan-1-ol. (**A**) The major product when ST07A is expressed in yeast has the same retention time as both (−)- and (+)-caryolan-1-ol in a general purpose GC column. (**B**) However, use of a chiral column confirmed that the major product of ST07A is the (−)-caryolan-1-ol enantiomer. (**C**) Mass spectra of the major product of ST07A and (−)- and (+)-caryolan-1-ol are the same.

Recently, a (+)-caryolan-1-ol synthase was reported from bacteria, *Streptomyces griseus* [38], and the mass spectrum of that compound was very similar to the major product of ST07 and ST07A. In contrast, plants have been reported to produce the opposite enantiomer of (*E*)-caryophyllene, (−)-(*E*)-caryophyllene. Comparison with authentic standards of (−)-caryolan-1-ol and (+)-caryolan-1-ol in a general purpose GC column and a chiral column confirmed that the major product of ST07 and ST07A is (−)-caryolan-1-ol (β-caryophyllene alcohol) (Figures 7, S28 and S29). To our knowledge, this is the first example of a (−)-caryolan-1-ol synthase.

### Identification of α-zingiberene/β-sesquiphellandrene Oxidase

**Figure 8:**
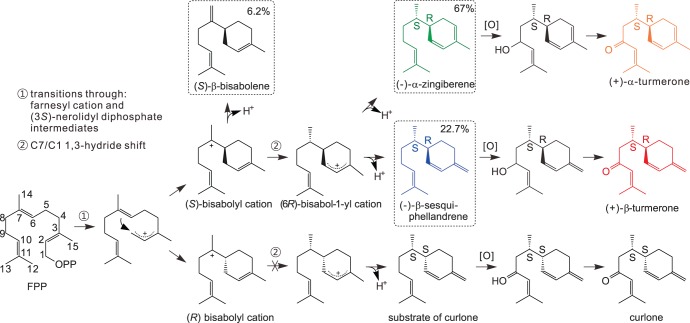
Proposed reaction of α-zingiberene/β-sesquiphellandrene hydroxylase. ST00A/ST00B produces (−)-α-zingiberene and (−)-β-sesquiphellandrene, which are hydroxylated by α-zingiberene/β-sesquiphellandrene hydroxylase, leading to the requirement of a dehydrogenase to make the ketone forms, (+)-α- and (+)-β-turmerones. The percentages shown inside the box represent ST00A/ST00B product percentage. The color used in this figure is compatible with the color used in Figure 1. Substrate of curlone: Cyclohexene, 3-[(1S)-1,5-dimethyl-4-hexen-1-yl]-6-methylene-, (3S)-.

As discussed above, (+)-α-turmerone and (+)-β-turmerone appear to be produced by the oxidation of (−)-α-zingiberene and (−)-β-sesquiphellandrene, respectively. By comparing EST data, microarray data and metabolite data, four P450 monooxygenases were selected from 170 ginger and turmeric P450s as the most likely candidates to be involved in formation of these two sesquiterpenoids. These four monooxygenases were named P1, P2, P3 and P4. P2 and P3 were partial clones in the EST database, missing 5′ ends, and genome walking revealed the complete sequences. P1, P2 and P4 are very similar to each other and belong to the clade (CYP71D) that contains limonene hydroxylase, which forms a secondary hydroxyl group. P3 is different from the other three P450s and it belongs to the clade (CYP71AV1) that contains amorphadiene oxidase. Amorphadiene oxidase catalyzes a three step of oxidation (primary alcohol → aldehyde → acid) at the end of a hydrocarbon chain [25] (although the latter two oxidations are much less favorable than the first) and limonene hydroxylase forms a secondary alcohol on a six carbon ring [39]. Oxygenation of (−)-α-zingiberene and (−)-β-sesquiphellandrene at position 9 (Figure 8) is a more similar reaction to that catalyzed by limonene hydroxylase than to that by amorphadiene oxidase. Therefore, P1, P2 and P4 seemed to be the best candidates for the first step in the conversion of (−)-α-zingiberene and (−)-β-sesquiphellandrene into (+)-α-turmerone and (+)-β-turmerone.

**Figure 9:**
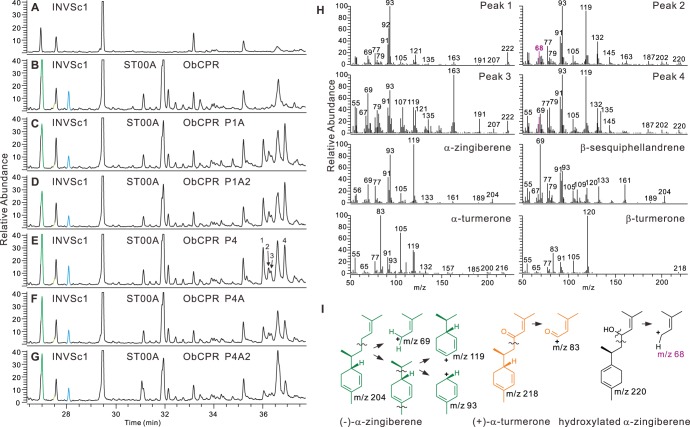
Analysis of the products of the putative α-zingiberene/β-sesquiphellandrene hydroxylase. P1A, P1A2, P4, P4A and P4A2 are P450 monooxygenase candidates for α-zingiberene/β-sesquiphellandrene hydroxylase. (**A**–**G**) Total ion chromatograms are shown for yeast cell line INVSc1 with or without plasmids: (**A**) INVSc1 with no plasmid; (**B**) INVSc1 with pESC-TRP-Ob_CPR and pESC-URA-ST00A; (**C**) INVSc1 with pESC-URA-ST00A-P1A; (**D**) INVSc1 with pESC-URA-ST00A-P1A2; (**E**) INVSc1 with pESC-URA-ST00A-P4; (**F**) INVSc1 with pESC-URA-ST00A-P4A; and (**G**) INVSc1 with pESC-URA-ST00A-P4A2. (**H**) Peaks 1, 2, 3 and 4 are new peaks resulting from the action of the P450 that yield mass spectra and are similar to (−)-α-zingiberene and (−)-β-sesquiphellandrene. (**I**) Proposed fragmentation pathway for (−)-α-zingiberene, (+)-α-turmerone, and the proposed hydroxylated intermediate.

Because all three enzymes were very similar, P1 and P4 were selected for cloning from turmeric rhizome cDNA, yielding five paralogs, P1A, P1A2, P4, P4A and P4A2, which were expressed in yeast. Co-expression of these P450s with ST00A in EPY224 that was not expressing a cytochrome P450 reductase gene did not produce hydroxylated forms of (−)-α-zingiberene or (−)-β-sesquiphellandrene. Co-expression of these P450s with ST00A and sweet basil P450 reductase (Ob_CPR) yielded some products that appear to be hydroxylated forms of (−)-α-zingiberene and (−)-β-sesquiphellandrene, according to comparison of mass spectra of these peaks with (−)-α-zingiberene, (−)-β-bisabolene and (−)-β-sesquiphellandrene (Figure 9). However, these hydroxylated products are not registered in the NIST GC/MS library, and unambiguous identification will require significant work in the future. Nevertheless, we can draw conclusions using our current data that suggest that these are indeed the intermediates of interest for the biosynthesis of the turmerones.

In Figure 9H, peak 2 and 4 have m/z 220, which is the mass of hydroxylated forms of (−)-α-zingiberene, (−)-β-bisabolene or (−)-β-sesquiphellandrene, and peak 1 and 3 have m/z 222. Although m/z 222 is not what would be expected for hydroxylated forms of (−)-α-zingiberene, α-bisabolol (MW 220) also contains m/z 222 in its mass spectrum. Other ions such as m/z 93, 119, 68, etc., also support the proposal that these peaks are likely to be hydroxylated forms of (−)-α-zingiberene, (−)-β-bisabolene or (−)-β-sesquiphellandrene. Because these peaks have different mass spectra than those of α-bisabolol, epi- α-bisabolol or β-bisabolol, it is likely that they are derived instead from (−)-α-zingiberene or (−)-β-sesquiphellandrene. Thus, the biosyntheses of (+)-α-turmerone and (+)-β-turmerone likely originates by the action of α-zingiberene/β-sesquiphellandrene synthase, followed by the action of α-zingiberene/β-sesquiphellandrene hydroxylase (P1, P2, or P4) and is completed by the action of a dehydrogenase (still uncharacterized) that converts the secondary alcohols to the ketone forms.

## Discussion

### Evolution of Terpene Synthases in Ginger and Turmeric Investigated through Protein Structural Modeling

We identified 25 mono- and 16 sesquiterpene synthases from ginger and turmeric and revealed the function of 13 mono- and 11 sesquiterpene synthases. The products of these TPSs and terpenoids in tissues correspond well except for a few instances, where for example high levels of citral (geranial+neral) were observed in ginger rhizomes and leaves, but no enzyme with geraniol synthase activity was observed. Geraniol is the precursor of citral. It is likely that one of the insoluble enzymes is a geraniol synthase.

**Figure 10:**
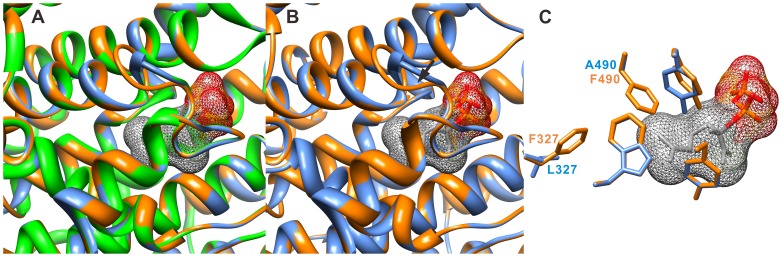
Comparison of MT06 and MT06B modeled structures. (**A**–**C**) MT06 and MT06B structures were modeled based on the structure of (4*S*)-limonene synthase from *Mentha spicata* as a template. (**A**) Alignment of all three structures in ribbon, (**B**) alignment of MT06 and MT06B in ribbon, and (**C**) side chains of MT06 and MT06B near bound ligand are shown. An additional amino acid (E573) in MT06B lies in the loop indicated with an arrow in **B** and does not appear to affect the structure. Y576 in MT06 and Y577 in MT06B are right after the loop and are aligned very well in **C**. Green: (4*S*)-limonene synthase from *M. spicata*, Blue: MT06, Orange: MT06B, Ligand with hashed surface: 2-fluorogeranyl diphosphate.

Many of the identified TPSs are very similar to each other and are considered to be paralogs, of which some have conserved functions whereas others have very divergent functions. For example, MT06 and MT06A were 98.0% identical and only had differences in the transit peptide sequences with one gap. Also, ST00A and ST00B, with 98.4% similarity, synthesized the same products. However, although MT06B is 92.9% identical to MT06 and 90.8% to MT06A, the products of these enzymes are quite different. MT06 and MT06A can produce sesquiterpenes whereas MT06B cannot. When MT06 and MT06B protein structures were modeled using (4S)-limonene synthase from *Mentha spicata* [40] as a template, their backbone structures were found to be very similar but the side chains in the substrate binding pocket were different (Figure 10). F327 of MT06B appears to prevent FPP binding whereas MT06 has a leucine at that position, which can allow FPP binding, leading to production of sesquiterpenes. Although MT06B has one extra amino acid in the loop (arrow in Figure 10B) when compared to MT06 and (4S)-limonene synthase, the tyrosines after the loop (Y576 in MT06, Y577 in MT06B) are aligned very well in the modeled structures and do not seem to affect protein function. These results suggest that replacement of F327 with a Leu may allow expanded substrate versatility and product production.

**Figure 11:**
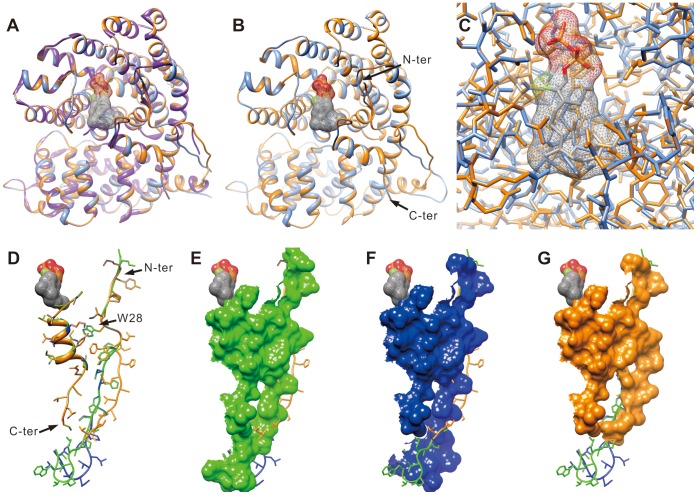
Comparison of ST02A4, ST02B and ST02C modeled structures. ST02A4, ST02B and ST02C structures were modeled with the structure of (+)-δ-cadinene synthase from *Gossypium arboreum* as a template. (**A**) Alignment of all three structures in ribbon, (**B**) alignment of ST02B and ST02C in ribbon, and (**C**) atoms/bonds of ST02B and ST02C are shown. C-terminal region and N-terminal regions of ST02B and ST02C are indicated with arrows in **B**. (**D**–**G**) Only the C-terminal region and N-terminal regions are shown. (**D**) The loop and helix structures with side chains are shown with W28 from the conserved RRX_8_W motif labeled. (**E**–**G**) Surfaces of (**E**) ST02A4, (**F**) ST02B and (**G**) ST02C are shown. Purple: (+)-δ-cadinene synthase from *G. arboreum*, Green: ST02A4, Blue: ST02B, Orange: ST02C, Ligand with hashed surface: 2-fluorofarnesyl diphosphate from aligned (+)-δ-cadinene synthase from *G. arboreum*.

ST02B and ST02C are 95.7% identical. However, ST02C produces β-elemene (germacrene A) as a major product and ST02B produces α-elemol as a major product and β-elemene (germacrene A) as a minor product. When their protein structures were modeled based on (+)-δ-cadinene synthase from *Gossypium arboreum* [41], we could not see side chain differences around the active sites in the ST02B and ST02C modeled structures (Figure 11). According to the modeled structures, the main differences lie in the N-terminal loop. The N-terminal end loop of ST02B is closer to the C-terminal end loop than was predicted for ST02C. The difference in contact of the N- and C-terminal end loops may affect protein breathing and cause easy access for water molecules to quench the reaction. ST02A4 is also very similar to ST02B and ST02C: 95.5% and 96.6% similarity, respectively. ST02A4 produces (−)-neointermedeol as a major product and β-elemene (germacrene A) as a minor product. When the ST02A4 structure was modeled against the (+)-δ-cadinene synthase structure and compared with the modeled ST02B and ST02C structures, there was no difference in side chains near the active site. Again, ST02A4 has a different loop structure at the N-terminal end. The expected structure for ST02A4 is more similar to ST02B than to ST02C because ST02C is expected to have an extended α-helix and shorter loop (Figure 11). The loop around the tryptophan at the N-terminal end of the RRX_8_W motif is in contact with the C-terminal end of the helix (Figure 11D) in all three modeled structures. This interaction may stabilize the C-terminal end structure near the active site. Although ST02A4 and ST02B synthesize different major products, both compounds are quenched by a water molecule, which can be explained by their similar loop structures at the N-terminal ends. ST02C and the template, (+)-δ-cadinene synthase, have similar loop structures at the N-terminal ends, prolonged α-helices and shorter loops, and both produce the terpenes not quenched by water molecules.

ST02A4, ST02B and ST02C appear to have diverged recently and their modeled structures are very similar. They also produce similar products: ST02A4 produces (−)-neointermedeol, ST02B produces α-elemol and ST02C produces β-elemene (germacrene A) as major products. These compounds are synthesized via the same mechanistic pathway through (+)-germacrene A (Figure 5).

### (+)-α-Turmerone and (+)-β-turmerone are Derived from (−)-α-zingiberene and (−)-β-sesquiphellandrene

During efforts to identify ginger and turmeric compounds eluted in our GC/MS analyses, four major sesquiterpenoids stood out as the most abundant in turmeric rhizomes. Two of these compounds were clearly (−)-α-zingiberene and (−)-β-sesquiphellandrene, also the major compounds in ginger rhizomes. The other two compounds were oxygenated, and had best hits to “tumerone” and “curlone” when searched against the NIST database. A search for tumerone (CAS# 180315-67-7) in SciFinder Scholar gave only two references: one from 1934 and the other in 1996 [23], which used analysis of fragmented masses in GC/MS but not NMR for structural characterization. No absolute configuration data were reported for this compound; thus the actual structure of tumerone was clearly in question. Our data suggest that the compound detected in our samples was instead (+)-α-turmerone, with absolute configuration of (6*R*, 7*S*). The absolute configuration of (−)-curlone (CAS# 87440-60-6), on the other hand, was proposed to be (6*S*, 7*S*) by dehydrogenation to form (+)-*ar*-turmerone (7*S*) and NOE correlations that were not shown in the original NMR-based characterization of that compound [42] that were said to indicate possible 6*S* configuration, based on some assumptions regarding molecule conformation that may not be valid. These results suggested that either (−)-curlone exists as a (6*S*, 7*S*) diastereomer of (+)-β-turmerone (6*R*, 7*S*), or it was incorrectly assigned the 6*S* configuration and (−)-curlone and (+)-β-turmerone are one and the same compound. The latter is very likely the case [43].

When we compared the terpenoid profiles of ginger and turmeric, we observed that both ginger and turmeric produce (−)-α-zingiberene and (−)-β-sesquiphellandrene, but only turmeric synthesizes the two oxygenated compounds. Ginger and turmeric produce more (−)-α-zingiberene than (−)-β-sesquiphellandrene and one oxygenated compound (α-turmerone) is also more abundant than the other oxygenated compound (β-turmerone, see Figure 1 and Figure 8). The ratios of these compounds are fairly consistent in all analyses. Indeed, when we view (−)-α-zingiberene (green) and (+)-α-turmerone (orange) as one group and (−)-β-sesquiphellandrene (blue) and (+)-β-turmerone (red) as a second group, the ratios of the first group to the second group are 2.7 and 2.9, respectively, in Figure 1D and 1E (in turmeric rhizomes), which is very similar to the ratio (3.2) of (−)-α-zingiberene to (−)-β-sesquiphellandrene in ginger rhizome (Figure 1A). This suggested that the two oxygenated compounds ((+)-α-turmerone and (+)-β-turmerone) could indeed be derived from (−)-α-zingiberene and (−)-β-sesquiphellandrene, respectively, and, based on absolute configuration of the compounds in question (all four share the 6*R*, 7*S* configuration), and the mechanism whereby the ketones are formed from the sesquiterpenes, which would not be expected to lead to inversion of configuration at C6, the oxygenated compounds present in the turmeric lines that we tested must indeed be (+)-α-turmerone and (+)-β-turmerone, even though they were known as tumerone and curlone in the NIST database. These results again point to the care that must be taken in such metabolite profiling experiments, where one should not rely solely on database searches to identify compounds in complex plant samples. It is possible that some turmeric accessions or plants do contain (−)-curlone, the diastereomer of (+)-β-turmerone. However, since it seems very unlikely that (−)-β-sesquiphellandrene is converted into (−)-curlone, either another route to (−)-curlone must exist in other turmeric lines, or the original paper describing (−)-curlone was incorrect in assignment of the configuration at C6. If (−)-curlone is present in other plants, its biosynthesis would involve the alternate intermediate, cyclohexene, 3-[(1S)-1,5-dimethyl-4-hexen-1-yl]-6-methylene-, (3S)- (CAS# 251318-35-1) (Figure 8), which has only been reported as a synthetic molecule [44]. (−)-β-Sesquiphellandrene and (−)-α-zingiberene are both produced by the STPS through an (*S*)-bisabolyl cation followed by a C7/C1 1,3-hydride shift (Figure 8). The precursor for (−)-curlone would have to be produced from the (*R*)-bisabolyl cation via a C7/C1 1,3-hydride shift. Thus, (−)-curlone synthesis (should it occur in nature) likely does not involve ST00A/ST00B, which appears to utilize only the (*S*)-bisabolyl cation as an enzyme-bound intermediate.

As discussed above, the cloned α-zingiberene/β-sesquiphellandrene synthases produce (−)-α-zingiberene and (−)-β-sesquiphellandrene with a similar ratio to that observed for the compounds measured in ginger and turmeric rhizome samples. Identification of these genes supports the conclusion that the blue colored peak in the metabolite profiling experiments (Figure 1) is (−)-β-sesquiphellandrene, leading to the conclusion that the red peak is (+)-β-turmerone instead of (−)-curlone.

## Supporting Information

Figure S1 **Analysis of MT06/MT06A functions when proteins were expressed in** ***E. coli*** **strain BL21 CodonPlus (DE3) RILP with FPP as a substrate.** Total ion chromatograms are displayed: pentane blank (A); enzyme assay using *E. coli* crude extract without pEXP5CT-MT06 or pEXP5CT-MT06A plasmid with FPP as a substrate (B); enzyme assay using *E. coli* crude extract expressing either MT06 (C) or MT06A (D) with FPP as a substrate. Mass spectra of peak 1, peak 6, peak 8, 7-epi-sesquithujene, cis-sesquisabinene hydrate, and trans-sesquisabinene hydrate from library, respectively. Retention time alignment of two retention times, one from Adam's library (x-axis) and the other from our sample (y-axis) (E), where orange dot represent unknown (7-epi-sesquithujene-like) (peak 1), red dot represents unknown (cis-sesquisabinene hydrate-like) (peak 6) and green dot represents unknown (trans-sesquisabinene hydrate-like1) (peak 8). Products/compounds identified include: 1, unknown (7-epi-sesquithujene-like); 2, γ-curcumene; 3, β-curcumeme; 4, β-sesquiphellandrene; 5, (*E*)-γ-bisabolene; 6, unknown (cis-sesquisabinene hydrate-like); 7, (*E*)-nerolidol; 8, unknown (trans-sesquisabinene hydrate-like1); 9, epi-β-bisabolol; 10, epi-α-bisabolol; 11, (*E*)-β-farnesene.
(TIF)

Figure S2 **Alignment of ginger and turmeric terpene synthases.** RRX8W and DDXXD motifs are marked in the quality curve.
(TIF)

Figure S3 **Analysis of ST00A and ST00B functions when proteins were expressed in** ***E. coli*** **strain BL21-AI RIL.** Total ion chromatograms are displayed: pentane blank (A, E); enzyme assay using *E. coli* crude extract without pH9GW-ST00A or pH9GW-ST00B plasmid with GPP (B) or FPP (F) as a substrate, respectively; enzyme assay using *E. coli* crude extract expressing ST00A with GPP (C) or FPP (G) as a substrate, respectively; enzyme assay using *E. coli* crude extract expressing ST00B with GPP (D) or FPP (H) as a substrate, respectively. Products/compounds identified include: 1, β-phellandrene; 2, α-pinene; 3, sabinene (4(10)-thujene); 4, β-pinene; 5, α-phellandrene; 6, limonene; 7, (−)-α-zingiberene; 8, (−)-β-sesquiphellandrene; 9, β-bisabolene; 10, unknown (trans-sesquisabinene hydrate-like2); *, unknown, which is unknown (7-epi-sesquithujene-like) from ST00A expression in the yeast strain, EPY219 (Figure 3, peak 5).
(TIF)

Figure S4 **Mass spectra for the peaks in** **Figure 3** **.** Products/compounds identified include: 1, α-zingiberene; 2, β-sesquiphellandrene; 3, β-bisabolene; 4, unknown (trans-sesquisabinene hydrate-like2); 5, unknown (7-epi-sesquithujene-like); 6, trans-α-bergamotene; 7, γ-curcumene; 8, *ar*-curcumene; 9, (*E*)-γ-bisabolene; 10, unknown (α-eudesmol-like); 11, γ-eudesmol; 12, unknown (trans-sesquisabinene hydrate-like3); 13, α-acorenol; 14, (*E*)-β-farnesene; 15, (*E*)-nerolidol.
(TIF)

Figure S5 **Analysis of MT08 function when protein was expressed in** ***E. coli*** **strain BL21 Star (DE3) pMevT pMBI RIL.** Total ion chromatograms are displayed: pentane blank (A, E); enzyme assay using *E. coli* crude extract from BL21 Star (DE3) pMevT pMBI RIL without pH9GW-Zc05I02tt with GPP (B); pentane extract from BL21 Star (DE3) pMevT pMBI RIL expressing MT08 (C, G), which represents in vivo activity of MT08; enzyme assay using *E. coli* crude extract from BL21 Star (DE3) pMevT pMBI RIL expressing MT08 with GPP (D); pentane extract from BL21 Star (DE3) pMevT pMBI RIL without pH9GW-Zc05I02tt (F). Here, Zc05I02 represents MT08 and "tt" in pH9GW-Zc05I02tt represents "truncated, thrombin", which means that the transit peptide was truncated and a thrombin cleavage site was introduced at the N-terminus of the MT08 gene. Products/compounds identified include: 1, β-phellandrene; 2, α-pinene; 3, (*Z*)-β-farnesene.
(TIF)

Figure S6 **Analysis of MT06B function when protein was expressed in** ***E. coli*** **strain BL21 CodonPlus (DE3) RILP.** Total ion chromatograms are displayed: pentane blank (A); enzyme assay using *E. coli* crude extract without pEXP5CT-MT06B plasmid with GPP (B) or FPP (D) as a substrate, respectively; enzyme assay using *E. coli* crude extract expressing MT06B with GPP (C) or FPP (E) as a substrate, respectively. A2, B2 and C2 are boxed regions from A, B and C panels to show very small peaks. D and E are shown to compare with MT06/MT06A. Products/compounds identified include: 1, camphene; 2, α-pinene; 3, limonene; 4, borneol (endo-borneol); 5, tricyclene; 6, β-pinene; 7, cis-sabinene hydrate; 8, *p*-mentha-1,4(8)-diene (terpinolene); 9, (*E*)-pinene hydrate ((*E*)-pinan-2-ol); 10, *p*-menth-1-en-8-ol (α-terpineol).
(TIF)

Figure S7 **Analysis of MT09A2 function when protein was expressed in** ***E. coli*** **strain BL21 CodonPlus (DE3) RILP.** Total ion chromatograms are displayed: pentane blank (A); enzyme assay with GPP using *E. coli* crude extract without pEXP5CT-MT09A2 plasmid (B) or expressing MT09A2 (C). A2, B2 and C2 are boxed regions from A, B and C panels to show very small peaks. Products/compounds identified include: 1, camphene; 2, α-pinene; 3, limonene; 4, borneol (endo-borneol); 5, tricyclene; 6, β-pinene; 7, γ-terpinene; 8, cis-sabinene hydrate; 9, *p*-mentha-1,4(8)-diene (terpinolene); 10, trans-pinene hydrate (trans-pinan-2-ol); 11, β-citronellal; 12, *p*-menth-1-en-8-ol (α-terpineol).
(TIF)

Figure S8 **Analysis of MT12A-M2 function when protein was expressed in** ***E. coli*** **strain BL21 CodonPlus (DE3) RILP.** Total ion chromatograms are displayed: pentane blank (A); enzyme assay with GPP using *E. coli* crude extract without pEXP5CT-MT12A-M2 plasmid (B) or expressing MT12A-M2 (C). A2, B2 and C2 are boxed regions from A, B and C panels to show very small peaks. Products/compounds identified include: 1, camphene; 2, α-pinene; 3, limonene; 4, borneol (endo-borneol); 5, tricyclene; 6, β-pinene; 7, cis-sabinene hydrate; 8, *p*-mentha-1,4(8)-diene (terpinolene); 9, trans-pinene hydrate (trans-pinan-2-ol); 10, *p*-menth-1-en-8-ol (α-terpineol).
(TIF)

Figure S9 **Analysis of MT04 function when protein was expressed in** ***E. coli*** **strain BL21 CodonPlus (DE3) RILP.** Total ion chromatograms are displayed: pentane blank (A); enzyme assay using *E. coli* crude extract without pEXP5CT-MT04 plasmid with GPP (B) or FPP (D) as a substrate, respectively; enzyme assay using *E. coli* crude extract expressing MT04 with GPP (C) or FPP (E) as a substrate, respectively. A2, B2, C2, D2 and E2 are boxed regions from A, B, C, D and E panels to show very small peaks. Larger amounts of MT04 major products (α-pinene and β-pinene) are in 2 month old yellow ginger rhizome (F) than in 7 month old yellow ginger rhizome (G). MT04 expression level of 2 month old yellow ginger root from microarray data is 7 times higher than 7 month old yellow rhizome (6586 versus 928, Table S4). Products/compounds identified include: 1, α-pinene; 2, β-pinene; 3, limonene containing (R)-(+)-m-mentha-6,8-diene (sylvestrene)-like compound; 4, sabinene (4(10)-thujene); 5, 1,8-cineole (eucalyptol); 6, camphene.
(TIF)

Figure S10 **Analysis of MT11 function when protein was expressed in** ***E. coli*** **strain BL21 CodonPlus (DE3) RIL and BL21 Star (DE3) pMevT pMBI RIL.** Total ion chromatograms are displayed: pentane blank (A, E); enzyme assay using *E. coli* (BL21 CodonPlus (DE3) RIL) crude extract expressing MT11 without GPP (B) or with GPP (D); enzyme assay with GPP using *E. coli* crude extract without pCRT7CT-MT11 plasmid (C); pentane extract from BL21 Star (DE3) pMevT pMBI RIL not transformed with pCRT7CT-MT11 (F) or expressing MT11 (G), which represents in vivo activity of MT11. Products/compounds identified include: 1, 1,8-cineole; 2, *p*-menth-1-en-8-ol (α-terpineol); 3, α-pinene; 4, β-pinene.
(TIF)

Figure S11 **Analysis of MT03 function when protein was expressed in** ***E. coli*** **strain BL21 CodonPlus (DE3) RIL.** Total ion chromatograms are displayed: pentane blank (A); enzyme assay using *E. coli* crude extract with MT03 expression without substrate (B) or with GPP as a substrate (D); enzyme assay with GPP using *E. coli* crude extract without pET101/D-MT03 plasmid (C). A2, B2, C2 and D2 are boxed regions from A, B, C and D panels to show very small peaks. Mass spectra of peak 3 (E), β-phellandrene (F) and limonene (G) show β-phellandrene and limonene are co-eluted in peak 3. Products/compounds identified include: 1, α-phellandrene; 2, α-terpinene; 3, β-phellandrene (contains limonene); 4, γ-terpinene; 5, *p*-mentha-1,4(8)-diene (terpinolene); 6, geraniol acetate.
(TIF)

Figure S12 **Analysis of MT07 function when protein was expressed in** ***E. coli*** **strain BL21 CodonPlus (DE3) RILP.** Total ion chromatograms are displayed: pentane blank (A); enzyme assay with GPP using *E. coli* crude extract without pEXP5CT-MT07 plasmid (B) or expressing MT07 (C). Products/compounds identified include: 1, *p*-mentha-1,4(8)-diene (terpinolene); 2, α-phellandrene; 3, 3-carene; 4, α-terpinene; 5, limonene; 6, γ-terpinene.
(TIF)

Figure S13 **Analysis of MT00 function when protein was expressed in** ***E. coli*** **.** Total ion chromatograms are displayed: pentane blank (A, F and I); *E. coli* (Rosetta2 (DE3) pLysS) crude extract without the pH9GW-MT00 plasmid with GPP (B); *E. coli* crude extract expressing MT00 with GPP (C) or FPP (E) as a substrate, respectively; *E. coli* crude extract without the pH9GW-MT00 plasmid with FPP (D); enzyme assay with purified MT00 from Rosetta2 (DE3) pLysS containing pH9GW-MT00 with GPP (G) or FPP (H) as a substrate, respectively; pentane extraction from BL21 Star (DE3) pMevT pMBI RIL cells with the pCRT7CT-MT00 plasmid without induction (J) or with induction (K), which represent in vivo activities of MT00. Products/compounds identified include: 1, linalool; 2, (*E*)-nerolidol; 3, trans-geraniol; 4, geranial (α-citral); 5, neral (β-citral); *, acetated form of linalool and (*E*)-nerolidol.
(TIF)

Figure S14 **Analysis of MT17A2 function when protein was expressed in** ***E. coli*** **strain BL21 CodonPlus (DE3) RILP.** Total ion chromatograms are displayed: pentane blank (A); enzyme assay using *E. coli* crude extract without pEXP5CT-MT17A2 plasmid with GPP (B) or FPP (D) as a substrate, respectively, or expressing MT17A2 with GPP (C) or FPP (E) as a substrate, respectively. A2, D2 and E2 are boxed regions from A, D and E panels to show very small peaks. Products/compounds identified include: 1,linalool; 2, unknown (7-epi-sesquithujene-like); 3, cis-α-bergamotene; 4, trans-α-bergamotene; 5, γ-curcumene; 6, unknown (β-sesquiphellandrene-like); 7, cis-α-bisabolene; 8, β-bisabolene; 9, β-sesquiphellandrene; 10, unknown (cis-α-bisabolene-like), 11, (*E*)-nerolidol; 12, epi-α-bisabolol; 13, α-bisabolol; 14, (*E*)-β-farnesene; 15, (*E*,*E*)-α-farnesene.
(TIF)

Figure S15 **Analysis of MT06/MT06A functions when proteins were expressed in** ***E. coli*** **strain BL21 CodonPlus (DE3) RILP with GPP as a substrate.** Total ion chromatograms are displayed: pentane blank (A); enzyme assay using *E. coli* crude extract without pEXP5CT-MT06 or pEXP5CT-MT06A plasmid with GPP as a substrate (B); enzyme assay using *E. coli* crude extract expressing either MT06 (C) or MT06A (D) with GPP as a substrate. Products/compounds identified include: 1, sabinene (4(10)-thujene); 2, α-terpinene; 3, γ-terpinene; 4, cis-sabinene hydrate; 5, *p*-mentha-1,4(8)-diene (terpinolene); 6, linalool; 7, *p*-menth-1-en-4-ol (terpinen-4-ol); 8, *p*-menth-1-en-8-ol (α-terpineol); 9, α-thujene (3-thujene); 10, myrcene.
(TIF)

Figure S16 **Analysis of MT02A function when protein was expressed in yeast strain EPY224.** Total ion chromatograms are displayed: pentane blank (A); EPY224 with MT02A expression (B); EPY224 without the pESC-URA-MT02A plasmid (C). The boxed regions of A, B and C are enlarged in A2, B2 and C2 panels to show very small peaks. Products/compounds identified include: 1, epi-α-bisabolol; 2, α-bisabolol; 3, (*E*)-nerolidol; 4, farnesol; 5, (*Z*)-α-bisabolene; 6, β-bisabolene; 7, (*E*)-α-bergamotene.
(TIF)

Figure S17 **Analysis of ST01 function when protein was expressed in** ***E. coli*** **strain BL21 CodonPlus (DE3) RIL and BL21 CodonPlus (DE3) RILP.** Total ion chromatograms are displayed: pentane blank (A); enzyme assay using *E. coli* (BL21 CodonPlus (DE3) RIL) crude extract expressing ST01 by pET101/D-ST01 plasmid without FPP (B) or with FPP as a substrate (D); enzyme assay using *E. coli* (BL21 CodonPlus (DE3) RILP) crude extract without either pET101/D-ST01 or pEXP5CT-ST01 plasmid with FPP as a substrate (C); enzyme assay using *E. coli* (BL21 CodonPlus (DE3) RILP) crude extract expressing ST01 by pEXP5CT-ST01 plasmid with FPP as a substrate (E). Products/compounds identified include: 1, β-selinene (eudesma-4(14),11-diene); 2, 7-epi-α-selinene; 3, β-elemene; 4, unknown (eremophila-1(10),11-diene-like); 5, unknown (β-chamigrene-like); 6, unknown (guaia-1(5),7(11)-diene-like); 7, (+)-intermedeol; *, unknown.
(TIF)

Figure S18 **Analysis of ST02A4 function when protein was expressed in yeast strain EPY224.** Total ion chromatograms are displayed: pentane blank (A); EPY224 without pESC-URA-ST02A4 plasmid (B); EPY224 expressing ST02A4 (C). Products/compounds identified include: 1, (−)-neointermedeol; 2, β-elemene; 3, δ-elemene; 4, (*E*)-caryophyllene; 5, γ-elemene; 6, germacrene D; 7, α-muurolene; 8, δ-cadinene (cadina-1(10),4-diene); 9, unknown (selina-6-en-4-ol-like); 10, unknown (cubenol-like); 11, unknown (spathulenol-like); 12, γ-eudesmol; 13, epi-α-muurolol (τ-muurolol); 14, α-muurolol (δ-cadinol); 15, α-cadinol; 16, (+)-intermedeol; 17, (*E*)-β-farnesene; 18, (*E*)-nerolidol; *, unknown.
(TIF)

Figure S19 **Analysis of ST02B function when protein was expressed in** ***E. coli*** **strain BL21 CodonPlus (DE3) RIL with GPP as a substrate.** Total ion chromatograms are displayed: pentane blank (A); enzyme assay using *E. coli* crude extract expressing ST02B without GPP (B) or with GPP as a substrate (D); enzyme assay using *E. coli* crude extract without pET101/D-ST02B plasmid with GPP as a substrate (C). Products/compounds identified include: 1, myrcene; 2, limonene; 3, (*Z*)-β-ocimene; 4, (*E*)-β-ocimene; 5, *p*-mentha-1,4(8)-diene (terpinolene); 6, linalool; 7, *p*-menth-1-en-8-ol (α-terpineol).
(TIF)

Figure S20 **Analysis of ST02B function when protein was expressed in** ***E. coli*** **strain BL21 CodonPlus (DE3) RIL with FPP as a substrate.** Total ion chromatograms are displayed: pentane blank (A); enzyme assay using *E. coli* crude extract expressing ST02B without FPP (B) or with FPP (D, E); enzyme assay using *E. coli* crude extract without pET101/D-ST02B plasmid with FPP (C). Enzyme assay was performed with pentane overlaid. After 3 hours of 30°C incubation, the top pentane layer was removed and directly injected into the GC/MS (D) or the whole enzyme assay including the top pentane was vortexed, centrifuged and the pentane phase collected and injected into the GC/MS (E). Products/compounds identified include: 1, α-elemol; 2, unknown ((+)-cyclosativene-like); 3, α-copaene; 4, β-elemene; 5, γ-elemene; 6, α-muurolene; 7, δ-cadinene (cadina-1(10),4-diene); 8, germacrene B; 9, germacrene D; *, unknown.
(TIF)

Figure S21 **Analysis of ST02C function when protein was expressed in** ***E. coli*** **strain BL21 CodonPlus (DE3) RIL with GPP as a substrate.** Total ion chromatograms are displayed: pentane blank (A); enzyme assay using *E. coli* crude extract expressing ST02C without GPP (B) or with GPP as a substrate (D), enzyme assay using *E. coli* crude extract without pET101/D-ST02C plasmid with GPP as a substrate (C). Products/compounds identified include: 1, myrcene; 2, limonene; 3, (*Z*)-β-ocimene; 4, (*E*)-β-ocimene; 5, p-mentha-1,4(8)-diene (terpinolene); 6, linalool; 7, p-menth-1-en-8-ol (α-terpineol).
(TIF)

Figure S22 **Analysis of ST02C function when protein was expressed in** ***E. coli*** **strain BL21 CodonPlus (DE3) RIL with FPP as a substrate.** Total ion chromatograms are displayed: pentane blank (A); enzyme assay using *E. coli* crude extract without pET101/D-ST02C plasmid with FPP as a substrate (B); enzyme assay using *E. coli* crude extract expressing ST02C with FPP as a substrate (C). Products/compounds identified include: 1, β-elemene; 2, δ-elemene; 3, unknown ((+)-cyclosativene-like); 4, (+)-cyclosativene; 5, α-copaene; 6, cis-β-elemene; 7, (*E*)-caryophyllene; 8, γ-elemene; 9, γ-muurolene; 10, germacrene D; 11, α-muurolene; 12, δ-cadinene (cadina-1(10),4-diene); 13, germacrene B.
(TIF)

Figure S23 **Analysis of ST03 function when protein was expressed in** ***E. coli*** **strain BL21 CodonPlus (DE3) RIL with FPP as a substrate.** Total ion chromatograms are displayed: pentane blank (A); enzyme assay using *E. coli* crude extract expressing ST03 without FPP (B) or with FPP as a substrate (C). Products/compounds identified include: 1, γ-amorphene; 2, allo-aromadendrene; 3, γ-cadinene; 4, germacrene D-4-ol; 5, germacrene D.
(TIF)

Figure S24 **Analysis of ST03 functions with proteins expressed in** ***E. coli*** **strain BL21 CodonPlus (DE3) RIL and GPP as a substrate.** Total ion chromatograms are displayed: pentane blank (A); enzyme assay using *E. coli* crude extract expressing ST03 without GPP (B) or with GPP as a substrate (D); enzyme assay with GPP as a substrate using *E. coli* crude extract containing pET101/D-ST03 plasmid but ST03 expression was not induced (C). pET101/D vector has lac operator and basal expression of ST03 is restricted. Products/compounds identified include: 1,myrcene; 2, (*Z*)-β-ocimene; 3, *p*-mentha-1,4(8)-diene (terpinolene); 4, linalool; 5, cis-*p*-menth-2-en-1-ol; 6, *p*-menth-1-en-8-ol (α-terpineol); 7, limonene; 8, (*E*)-β-ocimene.
(TIF)

Figure S25 **Analysis of ST05/ST05A functions when proteins were expressed in** ***E. coli*** **strain BL21 CodonPlus (DE3) RILP.** Total ion chromatograms are displayed: pentane blank (A); enzyme assay with FPP as a substrate using *E. coli* crude extract without pEXP5CT-ST05 or pEXP5CT-ST05A plasmid (B); enzyme assay with FPP as a substrate using E. coli crude extract expressing ST05 (C); enzyme assay with FPP as a substrate using partially purified ST05A (D). Products/compounds identified include: 1, α-humulene (α-caryophyllene); 2, (*E*)-caryophyllene (β-caryophyllene); 3, β-elemene; 4, 1,5,9-trimethyl-1,5,9-cyclododecatriene; 5, (*E*)-β-farnesene.
(TIF)

Figure S26 **Analysis of ST07A function when protein was expressed in yeast strain EPY224.** Total ion chromatograms are displayed: pentane blank (A); pentane extract of EPY224 without pESC-URA-ST07A plasmid (B); pentane extract of EPY224 expressing ST07A (C). The boxed regions of A, B and C are enlarged in A2, B2 and C2 panels to show very small peaks. Products/compounds identified include: 1, (−)-caryolan-1-ol; 2, (*E*)-nerolidol; 3, farnesol; 4, (*E*)-caryophyllene; 5, α-humulene; 6, (*Z*)-β-farnesene.
(TIF)

Figure S27 **Analysis of ST07 function when protein was expressed in yeast strain EPY224.** Total ion chromatograms are displayed: pentane blank (A); pentane extract of EPY219 without pESC-URA-ST07 plasmid (B); pentane extract of EPY219 expressing ST07 (C); pentane extract of EPY224 expressing ST07A (D). A2, B2, C2 and D2 are single ion chromatograms (m/z 91) of A, B, C and D. The most abundant ion in the (*E*)-caryophyllene mass spectrum is m/z 91. Products/compounds identified include: 1, 2 and 5, unknown; 3 and 4, (−)-caryolan-1-ol; 6 and 7, (*E*)-caryophyllene.
(TIF)

Table S1 Primers for RACE. 5′ GSP, gene specific primers for 5′ RACE; 5′ N-GSP, nested gene specific primers for 5′ RACE; 3′ GSP, gene specific primers for 3′ RACE; 3′ N-GSP, nested gene specific primers for 3′ RACE.
(DOC)

Table S2 Primers for cloning full length genes and sub-cloning for expression. Under the Category column, pCR2.1, pCRT7CT, pEXP5CT, pET101D and pENTRD mean pCR2.1-TOPO, pCRT7CT-TOPO, pEXP5CT-TOPO, pET101/D-TOPO and pENTR/D-TOPO vectors, respectively. Primers with F (forward) and R (reverse) suffix were used to amplify PCR fragments, which were then inserted in these vectors. PCR fragments with primers from category pDONR207 were produced by the Gateway BP reaction with the pDONR207 vector. PCR fragments with primers from category pESC-URA were sub-cloned into the pESC-URA vector.
(DOC)

Table S3 Mono- and sesquiterpene synthases identified in the ginger and turmeric EST database created from cDNA libraries from different tissues: Rh, rhizome; R, root; L, leaf. The numbers in the turmeric and ginger columns represent EST number per cDNA library in the database for the corresponding unitrans. Four unitrans, MT10, MT13, MT14 and MT18 were considered to belong to other unitrans after close investigation. In the RACE column, 5 or 3 means that 5′ or 3′ RACE was required to obtain full-length clones and, **bolded and underlined** means RACE was finished. Each unitrans was cloned for further characterization from the grey boxed sample. MT00 and MT11 were subcloned from the original cDNA clones directly without requiring RT-PCR.
(DOC)

Table S4 Expression levels of ginger and turmeric terpene synthase unitrans based on microarray data. Probes for microarrays were designed from partial sequences of unitrans before RACEs revealed full sequences. Abbreviations are; GY, Yellow Ginger; F, turmeric variety Fat Mild Orange (FMO); T, turmeric variety Thin Yellow Aromatic (TYA); Rh, rhizome; R, root; L, leaf. TYA barely produces sesquiterpenes and is used as a control for microarray experiments. The chemical profiles of the FMO variety have no differences from the turmeric variety, Hawaiian Red Turmeric (HRT) used to clone genes. Both were clonally derived from the same original line.
(DOC)

Table S5 Vectors used to express various ginger or turmeric TPS proteins in either *E. coli* or yeast cells. Solubility of expression in *E. coli* was checked in Coomassie-stained gels. Solubility of expression in yeast was checked by Western blotting. Solubility is the ratio of total and soluble fractions. n/a means that the solubility was not evaluated. The vector and cell combinations marked with "*" were used for further analysis to identify the functions of specific proteins as outlined in the text.
(DOC)

Results S1 **Additional details regarding cloning, expression and characterization of specific TPS genes can be found here.**
(DOCX)
