# Supplementary figures and images for: Suites of Terpene Synthases Explain Differential Terpenoid Production in Ginger and Turmeric Tissues
Source: PLoS One. 2012 Dec 18;7(12):e51481. doi: 10.1371/journal.pone.0051481 (PMC3525583; doi:10.1371/journal.pone.0051481)

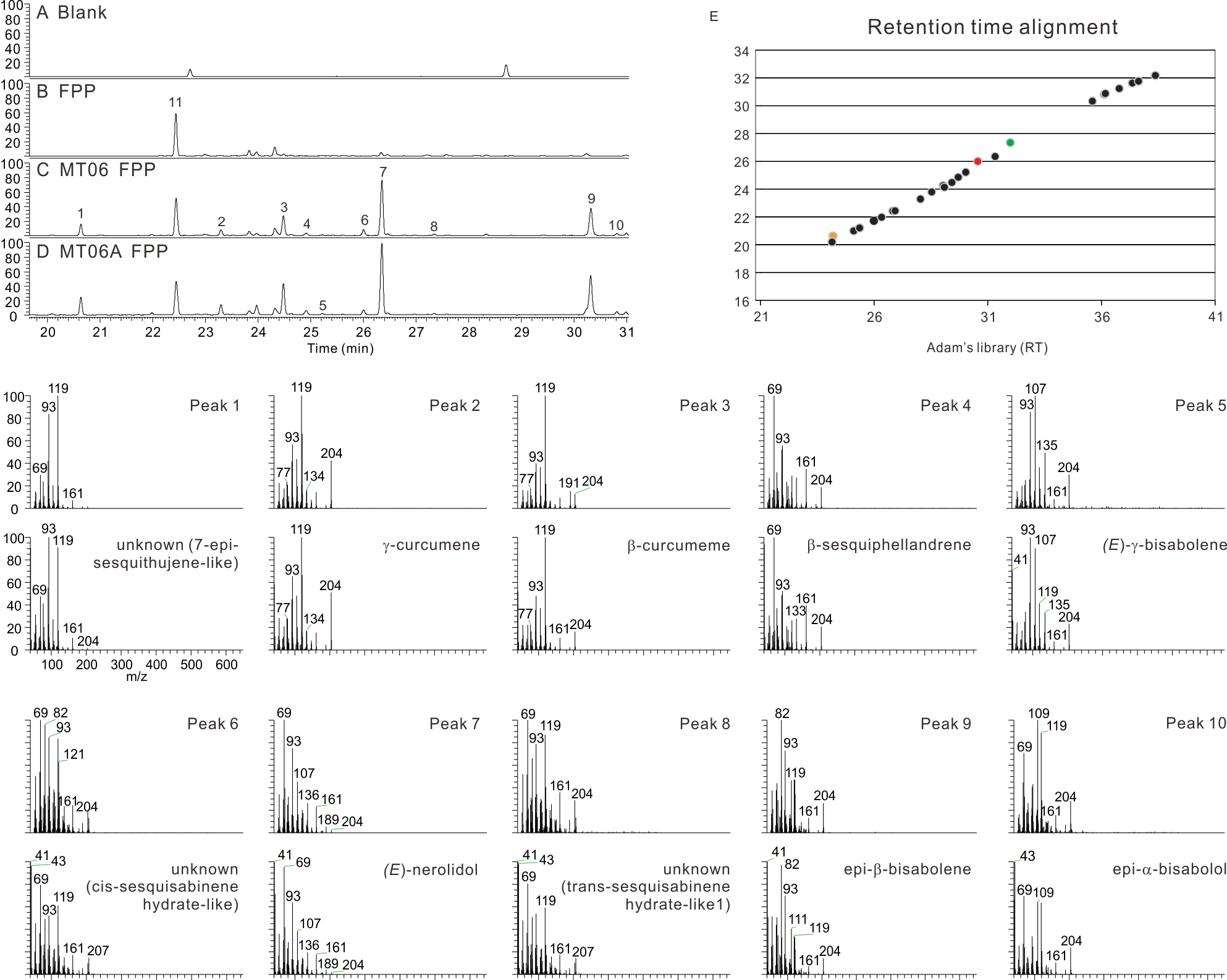

Supplement: Figure S1 — Analysis of MT06/MT06A functions when proteins were expressed in E. coli strain BL21 CodonPlus (DE3) RILP with FPP as a substrate. Total ion chromatograms are displayed: pentane blank (A); enzyme assay using E. coli crude extract without pEXP5CT-MT06 or pEXP5CT-MT06A plasmid with FPP as a substrate (B); enzyme assay using E. coli crude extract expressing either MT06 (C) or MT06A (D) with FPP as a substrate. Mass spectra of peak 1, peak 6, peak 8, 7-epi-sesquithujene, cis-sesquisabinene hydrate, and trans-sesquisabinene hydrate from library, respectively. Retention time alignment of two retention times, one from Adam's library (x-axis) and the other from our sample (y-axis) (E), where orange dot represent unknown (7-epi-sesquithujene-like) (peak 1), red dot represents unknown (cis-sesquisabinene hydrate-like) (peak 6) and green dot represents unknown (trans-sesquisabinene hydrate-like1) (peak 8). Products/compounds identified include: 1, unknown (7-epi-sesquithujene-like); 2, γ-curcumene; 3, β-curcumeme; 4, β-sesquiphellandrene; 5, (E)-γ-bisabolene; 6, unknown (cis-sesquisabinene hydrate-like); 7, (E)-nerolidol; 8, unknown (trans-sesquisabinene hydrate-like1); 9, epi-β-bisabolol; 10, epi-α-bisabolol; 11, (E)-β-farnesene. (TIF) [file pone.0051481.s001.tif]

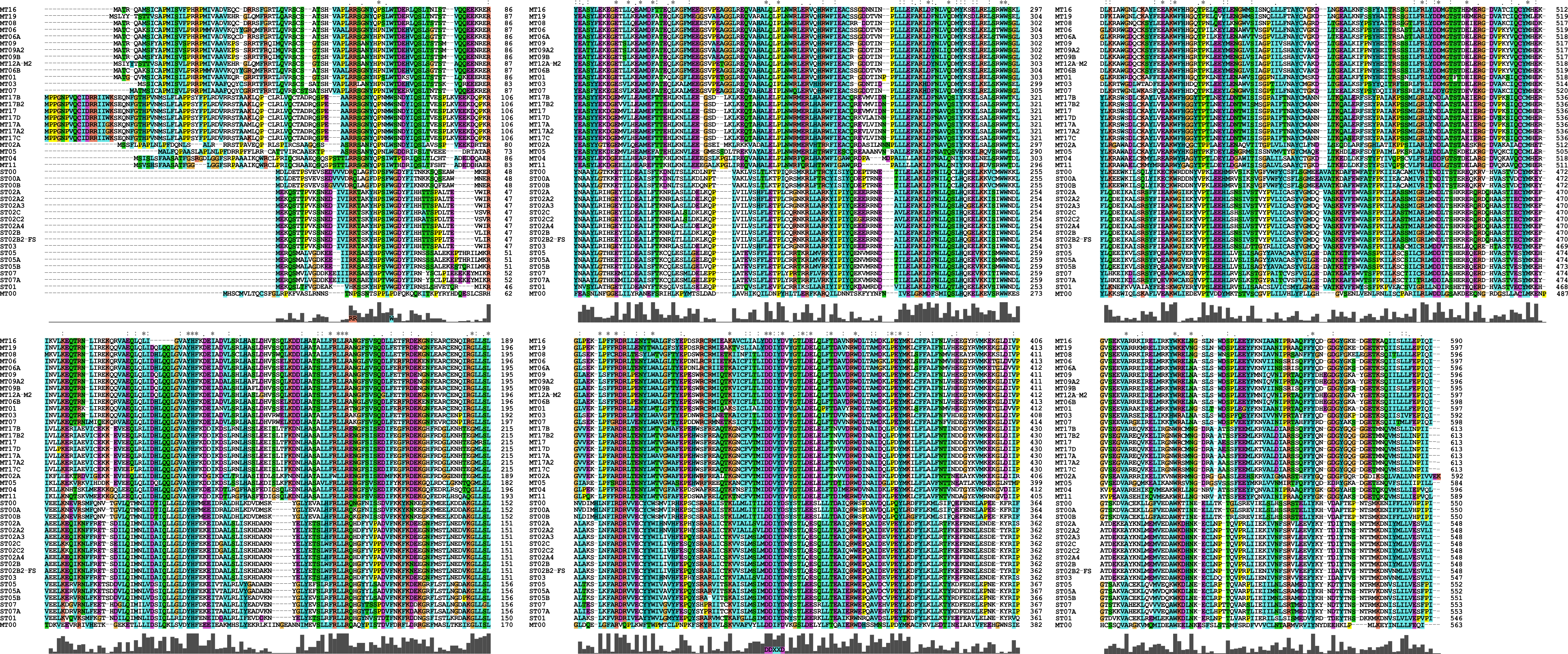

Supplement: Figure S2 — Alignment of ginger and turmeric terpene synthases. RRX8W and DDXXD motifs are marked in the quality curve. (TIF) [file pone.0051481.s002.tif]

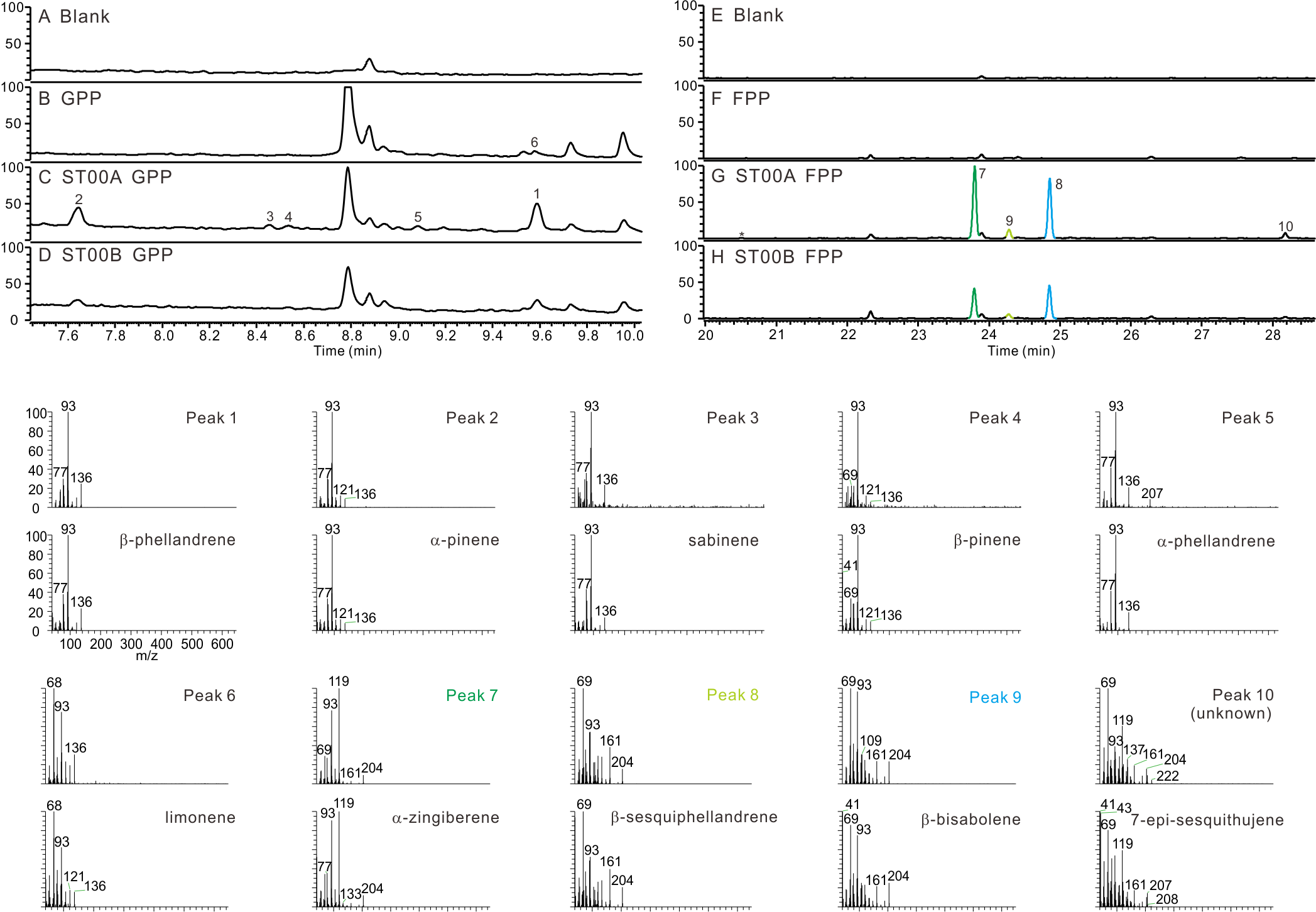

Supplement: Figure S3 — Analysis of ST00A and ST00B functions when proteins were expressed in E. coli strain BL21-AI RIL. Total ion chromatograms are displayed: pentane blank (A, E); enzyme assay using E. coli crude extract without pH9GW-ST00A or pH9GW-ST00B plasmid with GPP (B) or FPP (F) as a substrate, respectively; enzyme assay using E. coli crude extract expressing ST00A with GPP (C) or FPP (G) as a substrate, respectively; enzyme assay using E. coli crude extract expressing ST00B with GPP (D) or FPP (H) as a substrate, respectively. Products/compounds identified include: 1, β-phellandrene; 2, α-pinene; 3, sabinene (4(10)-thujene); 4, β-pinene; 5, α-phellandrene; 6, limonene; 7, (−)-α-zingiberene; 8, (−)-β-sesquiphellandrene; 9, β-bisabolene; 10, unknown (trans-sesquisabinene hydrate-like2); *, unknown, which is unknown (7-epi-sesquithujene-like) from ST00A expression in the yeast strain, EPY219 (Figure 3, peak 5). (TIF) [file pone.0051481.s003.tif]

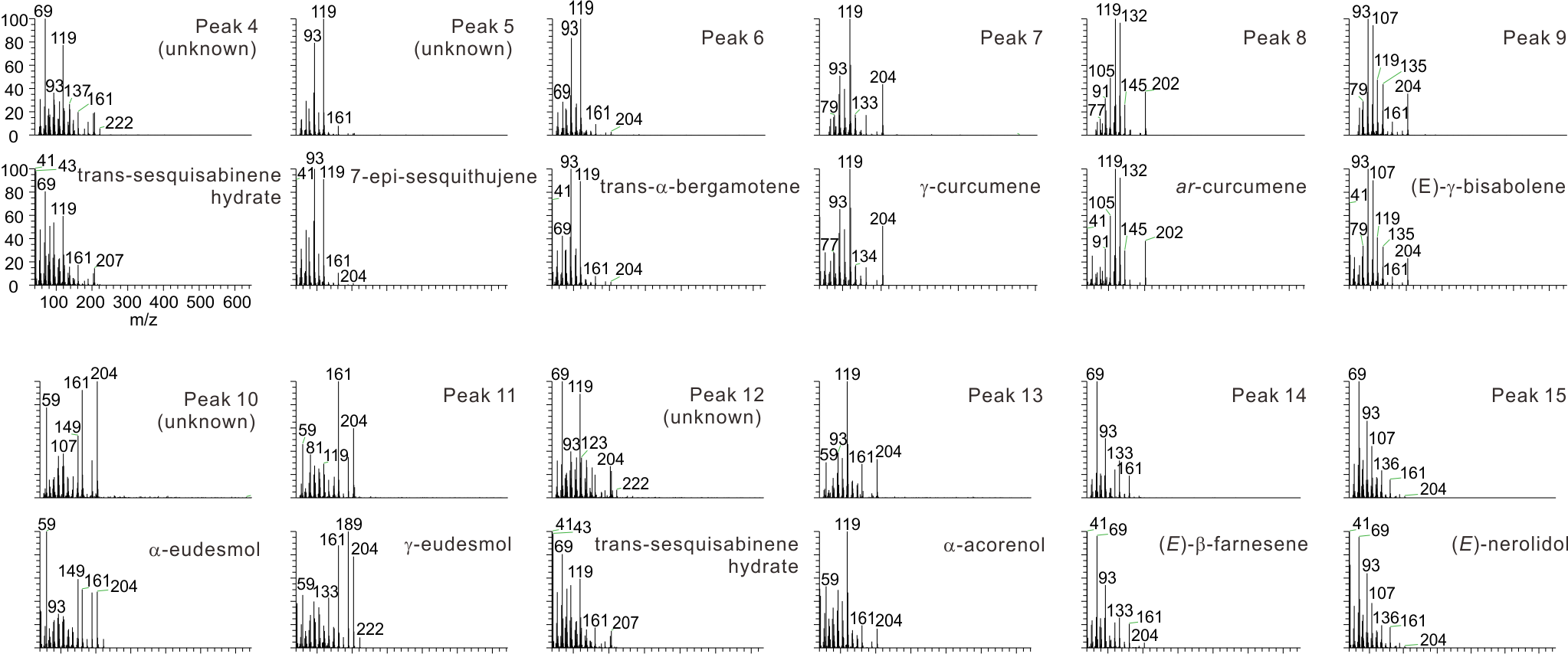

Supplement: Figure S4 — Mass spectra for the peaks in Figure 3 . Products/compounds identified include: 1, α-zingiberene; 2, β-sesquiphellandrene; 3, β-bisabolene; 4, unknown (trans-sesquisabinene hydrate-like2); 5, unknown (7-epi-sesquithujene-like); 6, trans-α-bergamotene; 7, γ-curcumene; 8, ar-curcumene; 9, (E)-γ-bisabolene; 10, unknown (α-eudesmol-like); 11, γ-eudesmol; 12, unknown (trans-sesquisabinene hydrate-like3); 13, α-acorenol; 14, (E)-β-farnesene; 15, (E)-nerolidol. (TIF) [file pone.0051481.s004.tif]

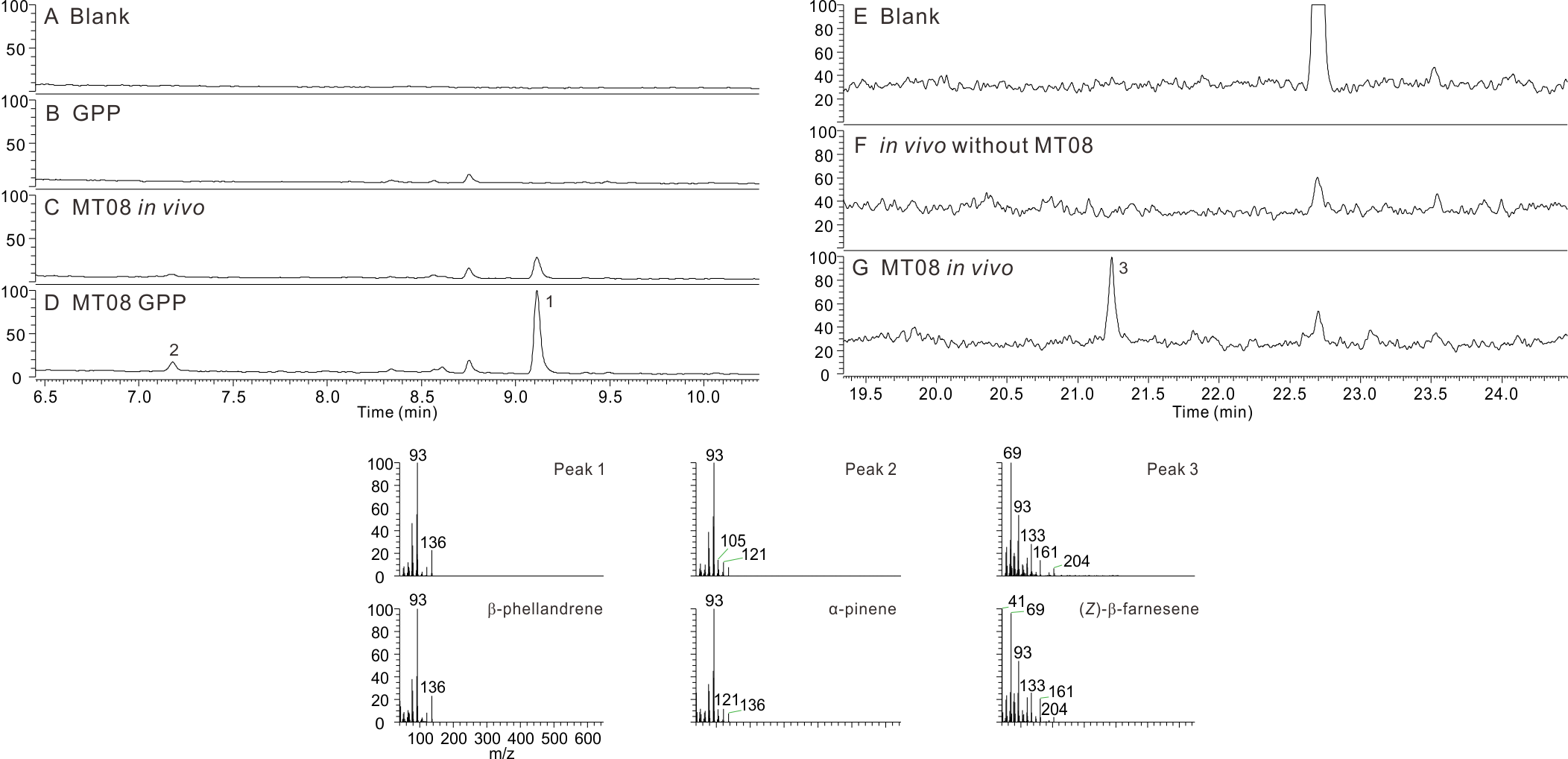

Supplement: Figure S5 — Analysis of MT08 function when protein was expressed in E. coli strain BL21 Star (DE3) pMevT pMBI RIL. Total ion chromatograms are displayed: pentane blank (A, E); enzyme assay using E. coli crude extract from BL21 Star (DE3) pMevT pMBI RIL without pH9GW-Zc05I02tt with GPP (B); pentane extract from BL21 Star (DE3) pMevT pMBI RIL expressing MT08 (C, G), which represents in vivo activity of MT08; enzyme assay using E. coli crude extract from BL21 Star (DE3) pMevT pMBI RIL expressing MT08 with GPP (D); pentane extract from BL21 Star (DE3) pMevT pMBI RIL without pH9GW-Zc05I02tt (F). Here, Zc05I02 represents MT08 and "tt" in pH9GW-Zc05I02tt represents "truncated, thrombin", which means that the transit peptide was truncated and a thrombin cleavage site was introduced at the N-terminus of the MT08 gene. Products/compounds identified include: 1, β-phellandrene; 2, α-pinene; 3, (Z)-β-farnesene. (TIF) [file pone.0051481.s005.tif]

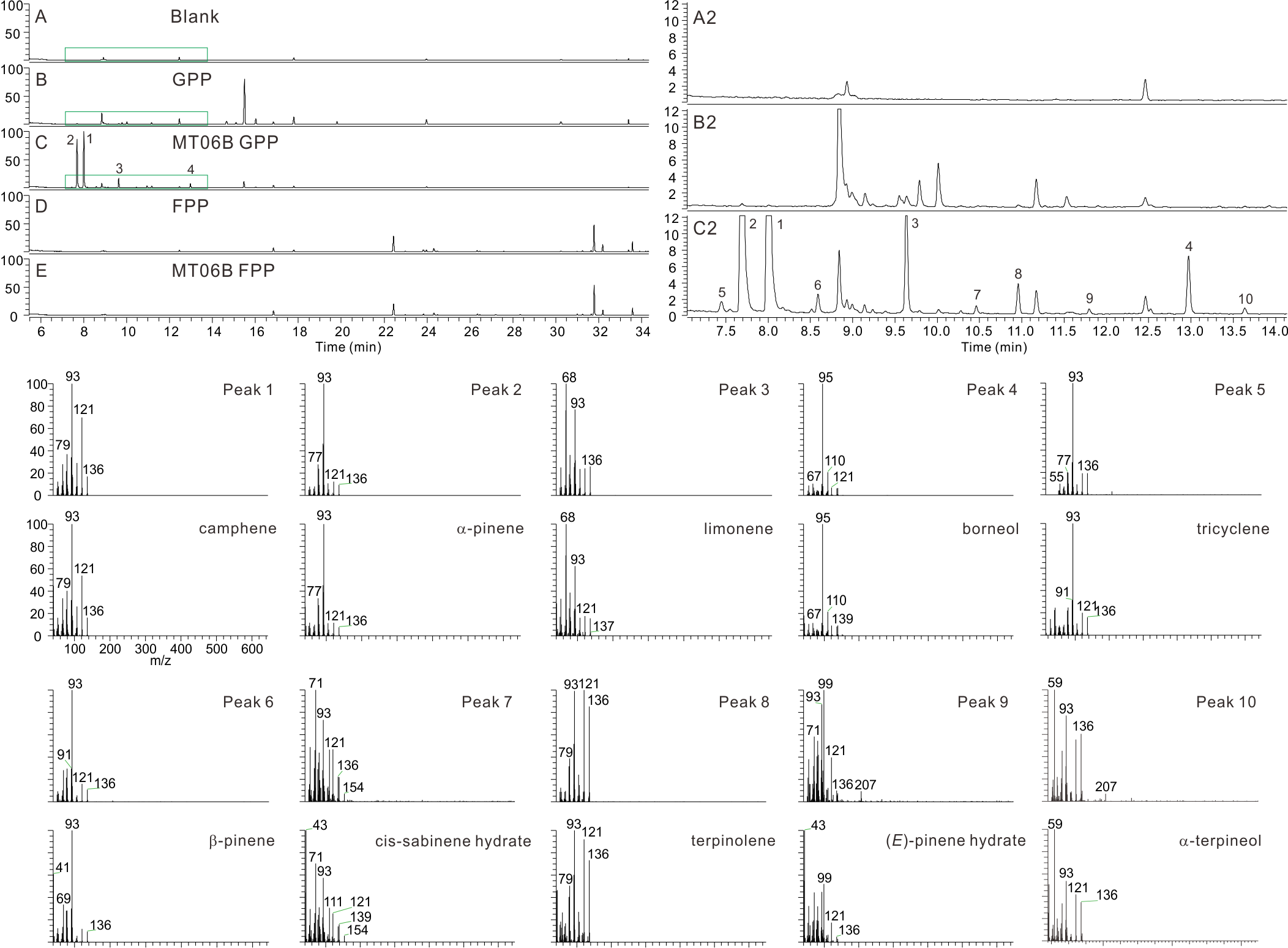

Supplement: Figure S6 — Analysis of MT06B function when protein was expressed in E. coli strain BL21 CodonPlus (DE3) RILP. Total ion chromatograms are displayed: pentane blank (A); enzyme assay using E. coli crude extract without pEXP5CT-MT06B plasmid with GPP (B) or FPP (D) as a substrate, respectively; enzyme assay using E. coli crude extract expressing MT06B with GPP (C) or FPP (E) as a substrate, respectively. A2, B2 and C2 are boxed regions from A, B and C panels to show very small peaks. D and E are shown to compare with MT06/MT06A. Products/compounds identified include: 1, camphene; 2, α-pinene; 3, limonene; 4, borneol (endo-borneol); 5, tricyclene; 6, β-pinene; 7, cis-sabinene hydrate; 8, p-mentha-1,4(8)-diene (terpinolene); 9, (E)-pinene hydrate ((E)-pinan-2-ol); 10, p-menth-1-en-8-ol (α-terpineol). (TIF) [file pone.0051481.s006.tif]

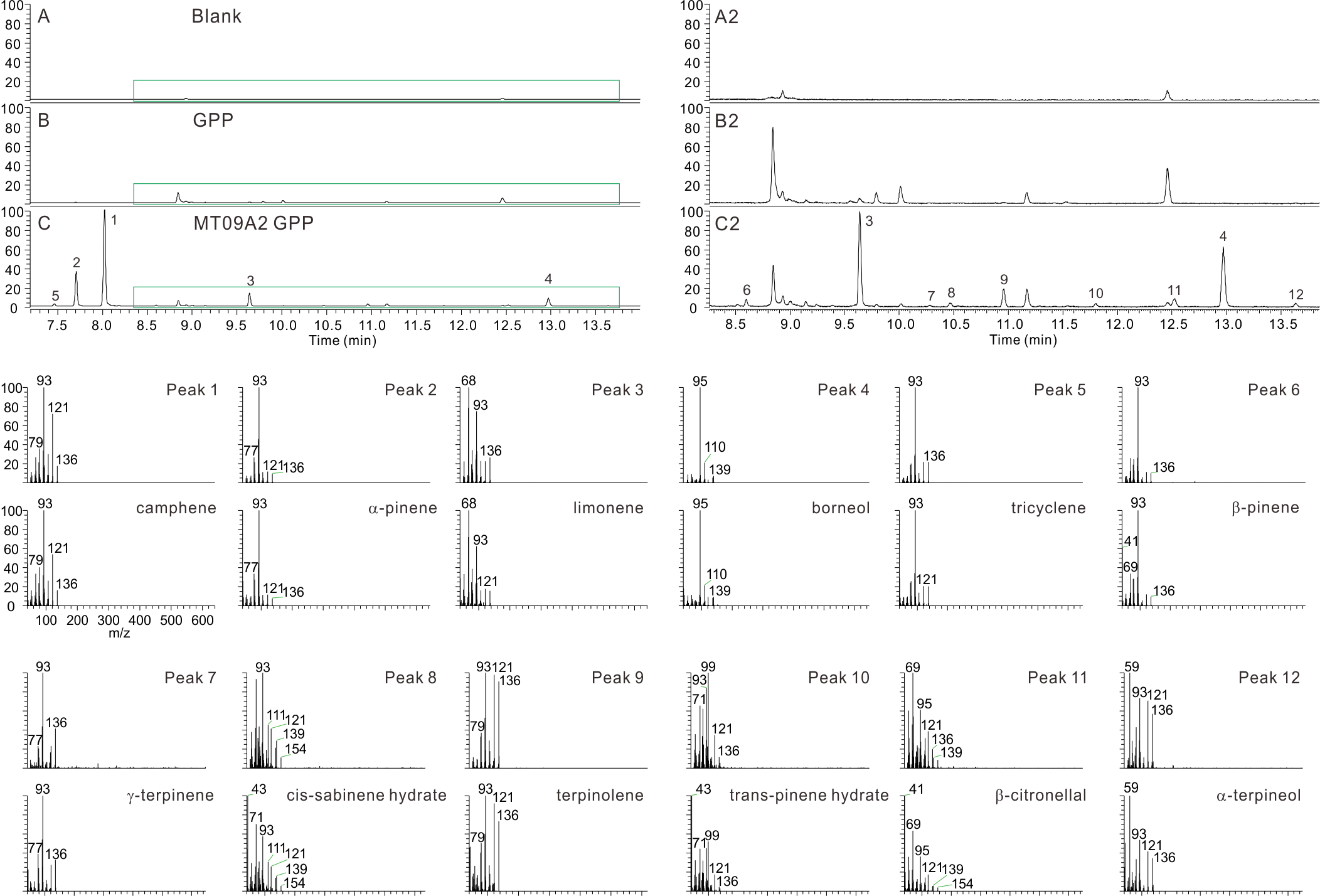

Supplement: Figure S7 — Analysis of MT09A2 function when protein was expressed in E. coli strain BL21 CodonPlus (DE3) RILP. Total ion chromatograms are displayed: pentane blank (A); enzyme assay with GPP using E. coli crude extract without pEXP5CT-MT09A2 plasmid (B) or expressing MT09A2 (C). A2, B2 and C2 are boxed regions from A, B and C panels to show very small peaks. Products/compounds identified include: 1, camphene; 2, α-pinene; 3, limonene; 4, borneol (endo-borneol); 5, tricyclene; 6, β-pinene; 7, γ-terpinene; 8, cis-sabinene hydrate; 9, p-mentha-1,4(8)-diene (terpinolene); 10, trans-pinene hydrate (trans-pinan-2-ol); 11, β-citronellal; 12, p-menth-1-en-8-ol (α-terpineol). (TIF) [file pone.0051481.s007.tif]

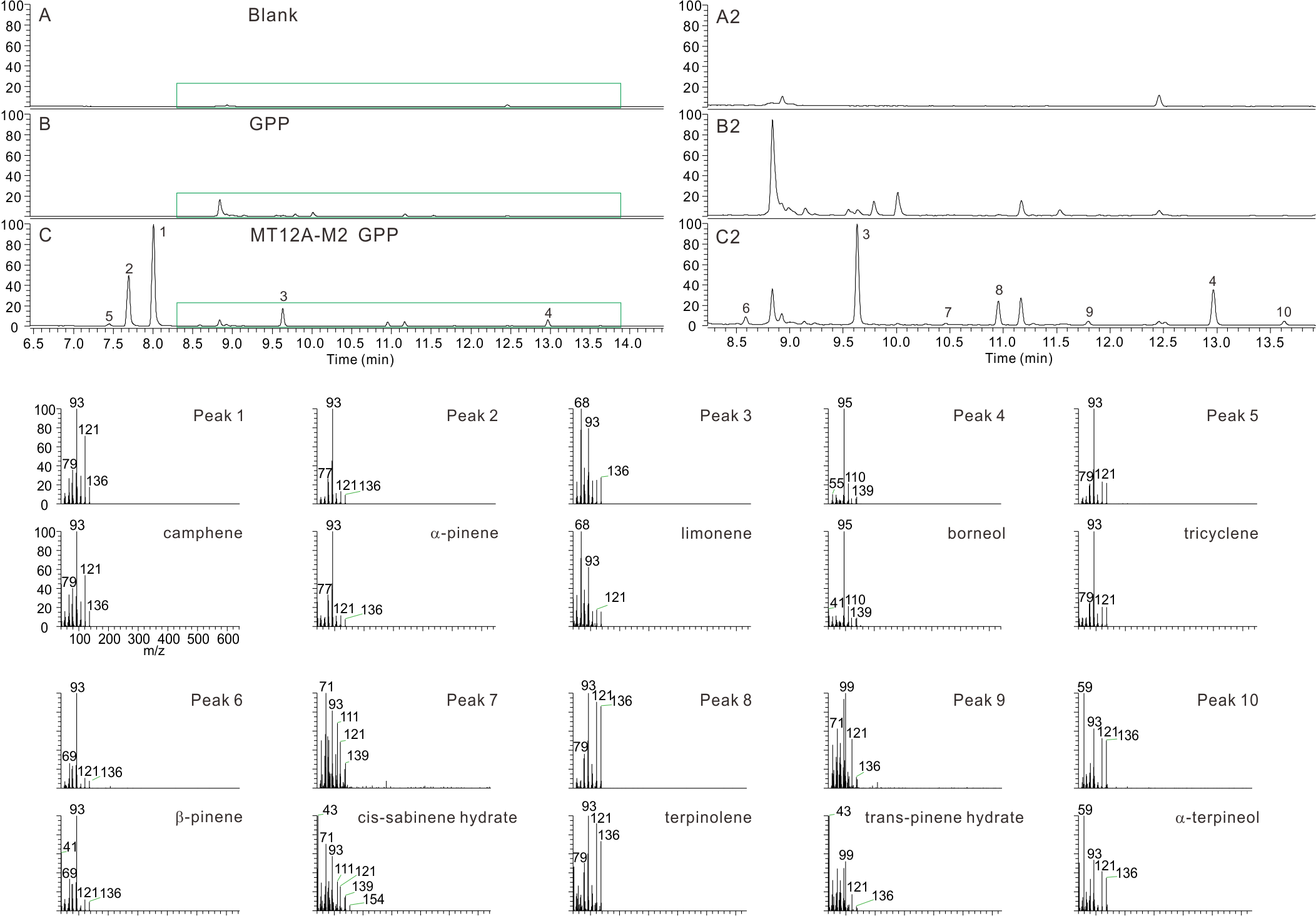

Supplement: Figure S8 — Analysis of MT12A-M2 function when protein was expressed in E. coli strain BL21 CodonPlus (DE3) RILP. Total ion chromatograms are displayed: pentane blank (A); enzyme assay with GPP using E. coli crude extract without pEXP5CT-MT12A-M2 plasmid (B) or expressing MT12A-M2 (C). A2, B2 and C2 are boxed regions from A, B and C panels to show very small peaks. Products/compounds identified include: 1, camphene; 2, α-pinene; 3, limonene; 4, borneol (endo-borneol); 5, tricyclene; 6, β-pinene; 7, cis-sabinene hydrate; 8, p-mentha-1,4(8)-diene (terpinolene); 9, trans-pinene hydrate (trans-pinan-2-ol); 10, p-menth-1-en-8-ol (α-terpineol). (TIF) [file pone.0051481.s008.tif]

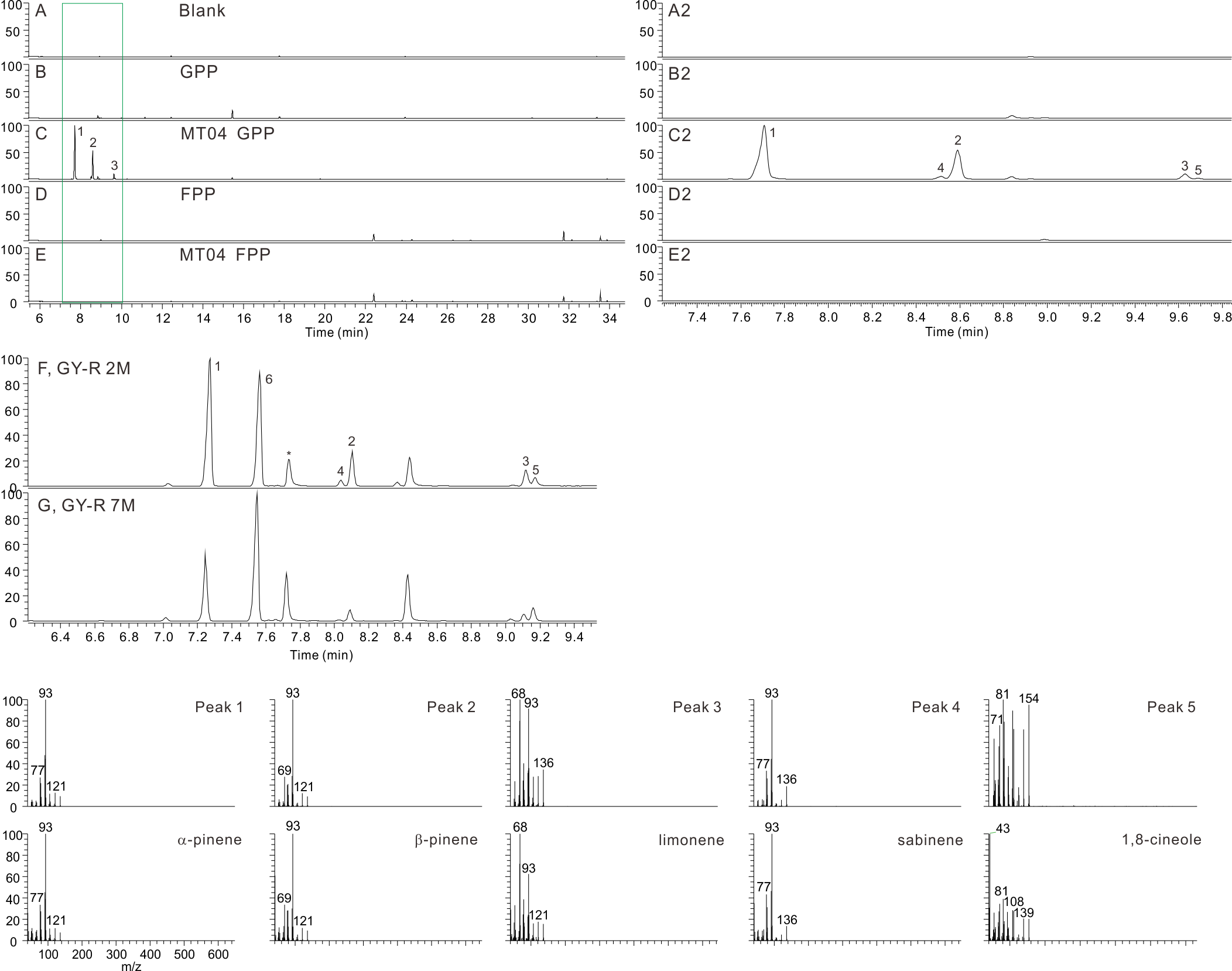

Supplement: Figure S9 — Analysis of MT04 function when protein was expressed in E. coli strain BL21 CodonPlus (DE3) RILP. Total ion chromatograms are displayed: pentane blank (A); enzyme assay using E. coli crude extract without pEXP5CT-MT04 plasmid with GPP (B) or FPP (D) as a substrate, respectively; enzyme assay using E. coli crude extract expressing MT04 with GPP (C) or FPP (E) as a substrate, respectively. A2, B2, C2, D2 and E2 are boxed regions from A, B, C, D and E panels to show very small peaks. Larger amounts of MT04 major products (α-pinene and β-pinene) are in 2 month old yellow ginger rhizome (F) than in 7 month old yellow ginger rhizome (G). MT04 expression level of 2 month old yellow ginger root from microarray data is 7 times higher than 7 month old yellow rhizome (6586 versus 928, Table S4). Products/compounds identified include: 1, α-pinene; 2, β-pinene; 3, limonene containing (R)-(+)-m-mentha-6,8-diene (sylvestrene)-like compound; 4, sabinene (4(10)-thujene); 5, 1,8-cineole (eucalyptol); 6, camphene. (TIF) [file pone.0051481.s009.tif]

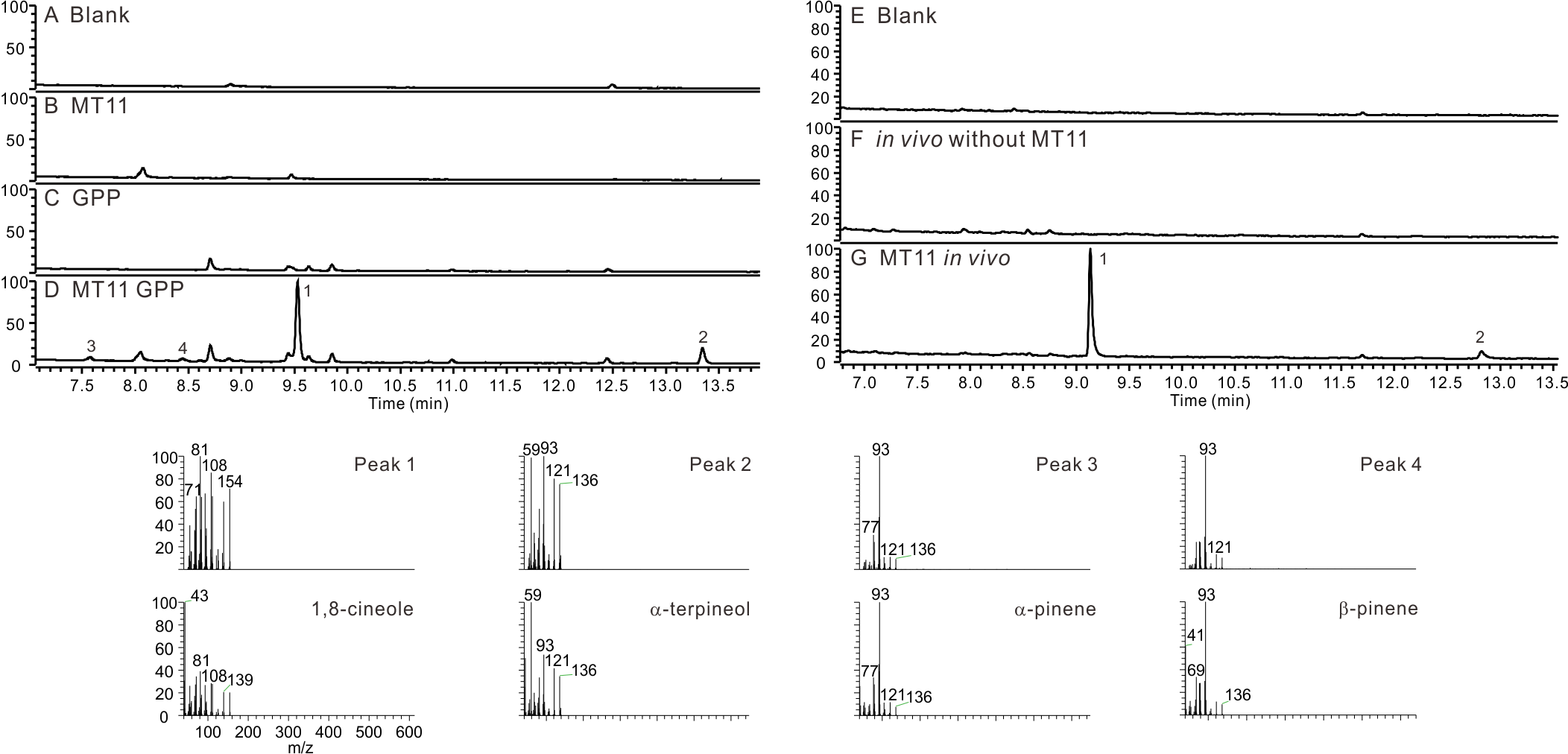

Supplement: Figure S10 — Analysis of MT11 function when protein was expressed in E. coli strain BL21 CodonPlus (DE3) RIL and BL21 Star (DE3) pMevT pMBI RIL. Total ion chromatograms are displayed: pentane blank (A, E); enzyme assay using E. coli (BL21 CodonPlus (DE3) RIL) crude extract expressing MT11 without GPP (B) or with GPP (D); enzyme assay with GPP using E. coli crude extract without pCRT7CT-MT11 plasmid (C); pentane extract from BL21 Star (DE3) pMevT pMBI RIL not transformed with pCRT7CT-MT11 (F) or expressing MT11 (G), which represents in vivo activity of MT11. Products/compounds identified include: 1, 1,8-cineole; 2, p-menth-1-en-8-ol (α-terpineol); 3, α-pinene; 4, β-pinene. (TIF) [file pone.0051481.s010.tif]

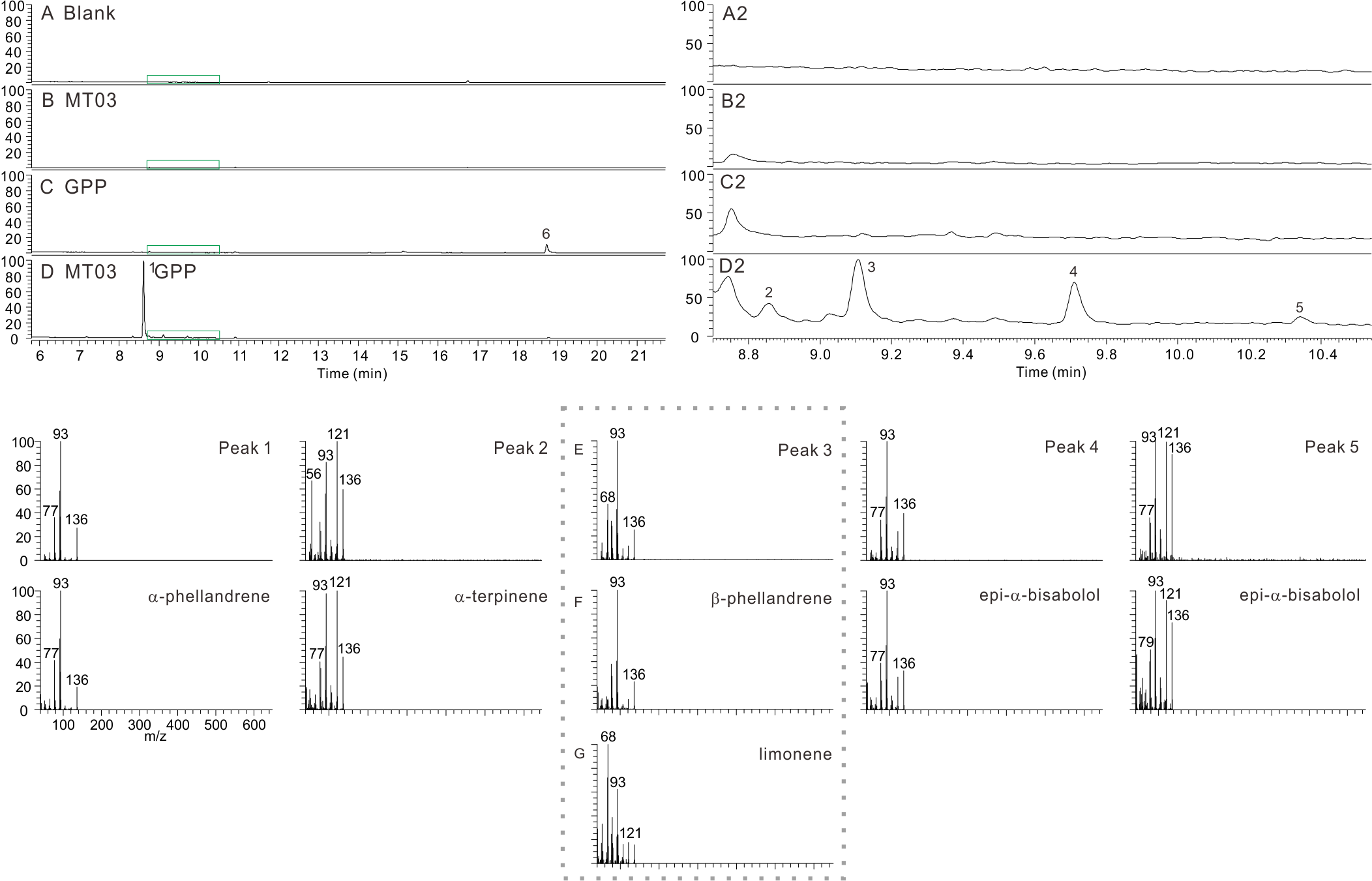

Supplement: Figure S11 — Analysis of MT03 function when protein was expressed in E. coli strain BL21 CodonPlus (DE3) RIL. Total ion chromatograms are displayed: pentane blank (A); enzyme assay using E. coli crude extract with MT03 expression without substrate (B) or with GPP as a substrate (D); enzyme assay with GPP using E. coli crude extract without pET101/D-MT03 plasmid (C). A2, B2, C2 and D2 are boxed regions from A, B, C and D panels to show very small peaks. Mass spectra of peak 3 (E), β-phellandrene (F) and limonene (G) show β-phellandrene and limonene are co-eluted in peak 3. Products/compounds identified include: 1, α-phellandrene; 2, α-terpinene; 3, β-phellandrene (contains limonene); 4, γ-terpinene; 5, p-mentha-1,4(8)-diene (terpinolene); 6, geraniol acetate. (TIF) [file pone.0051481.s011.tif]

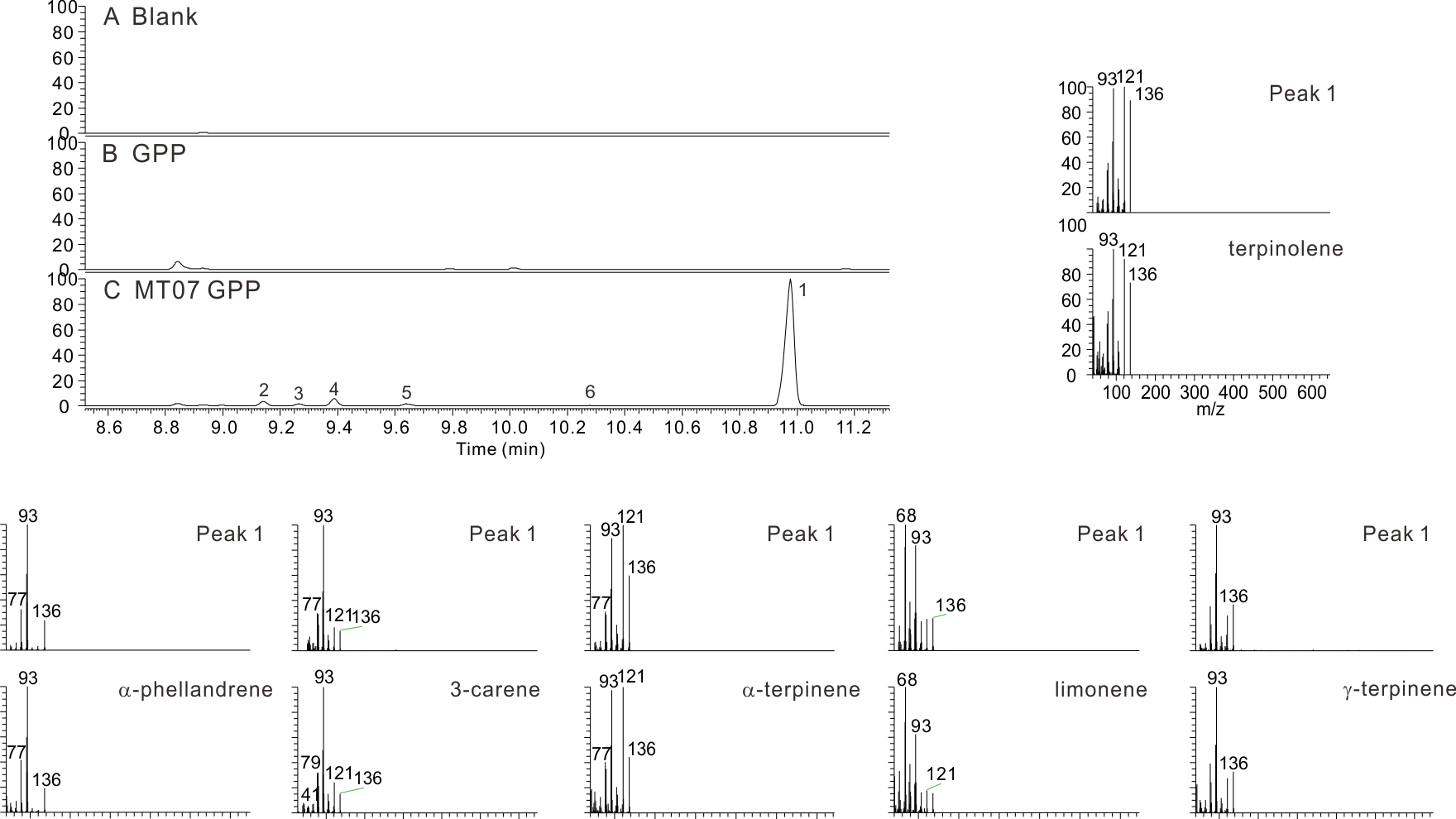

Supplement: Figure S12 — Analysis of MT07 function when protein was expressed in E. coli strain BL21 CodonPlus (DE3) RILP. Total ion chromatograms are displayed: pentane blank (A); enzyme assay with GPP using E. coli crude extract without pEXP5CT-MT07 plasmid (B) or expressing MT07 (C). Products/compounds identified include: 1, p-mentha-1,4(8)-diene (terpinolene); 2, α-phellandrene; 3, 3-carene; 4, α-terpinene; 5, limonene; 6, γ-terpinene. (TIF) [file pone.0051481.s012.tif]

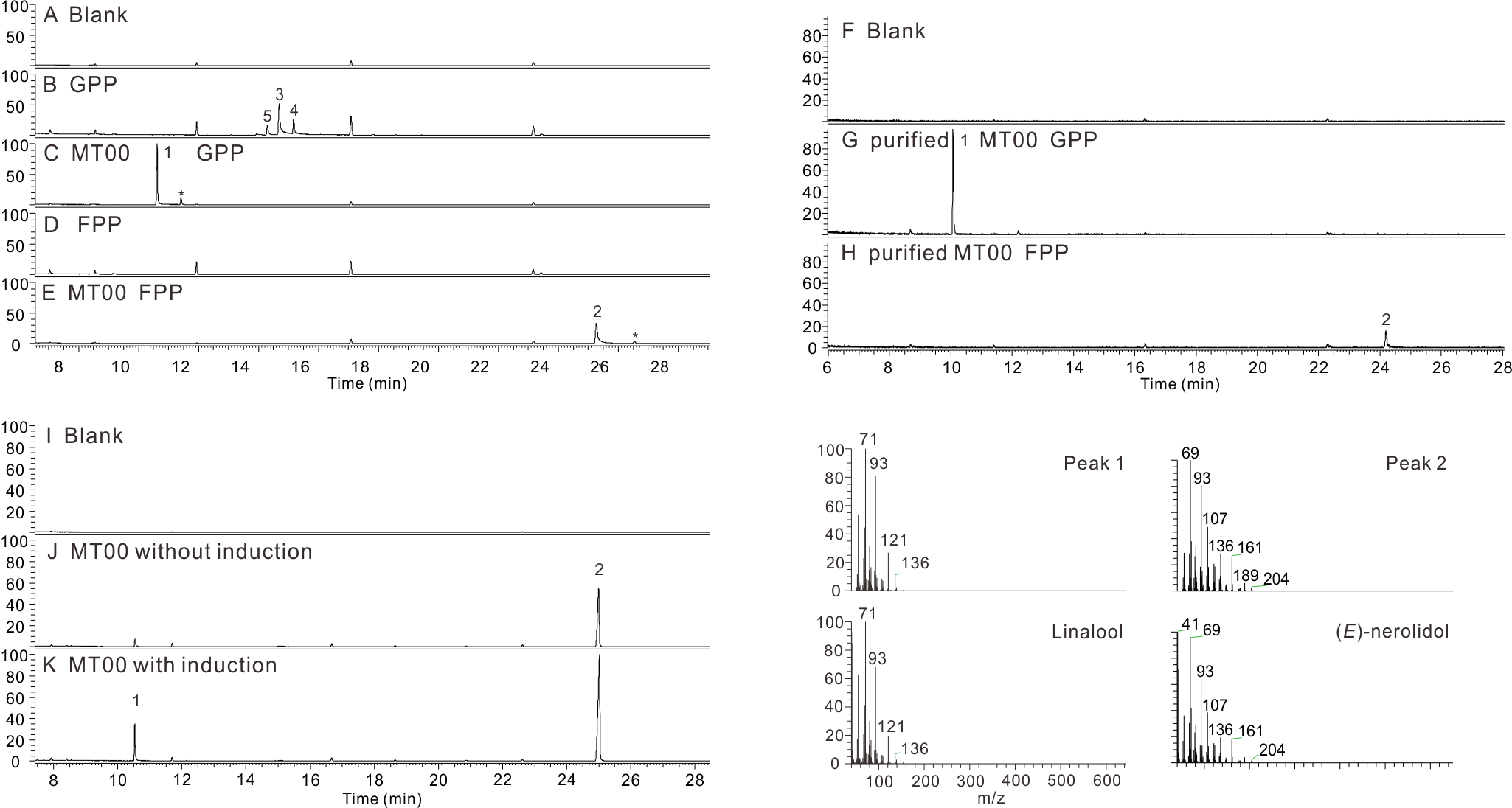

Supplement: Figure S13 — Analysis of MT00 function when protein was expressed in E. coli . Total ion chromatograms are displayed: pentane blank (A, F and I); E. coli (Rosetta2 (DE3) pLysS) crude extract without the pH9GW-MT00 plasmid with GPP (B); E. coli crude extract expressing MT00 with GPP (C) or FPP (E) as a substrate, respectively; E. coli crude extract without the pH9GW-MT00 plasmid with FPP (D); enzyme assay with purified MT00 from Rosetta2 (DE3) pLysS containing pH9GW-MT00 with GPP (G) or FPP (H) as a substrate, respectively; pentane extraction from BL21 Star (DE3) pMevT pMBI RIL cells with the pCRT7CT-MT00 plasmid without induction (J) or with induction (K), which represent in vivo activities of MT00. Products/compounds identified include: 1, linalool; 2, (E)-nerolidol; 3, trans-geraniol; 4, geranial (α-citral); 5, neral (β-citral); *, acetated form of linalool and (E)-nerolidol. (TIF) [file pone.0051481.s013.tif]

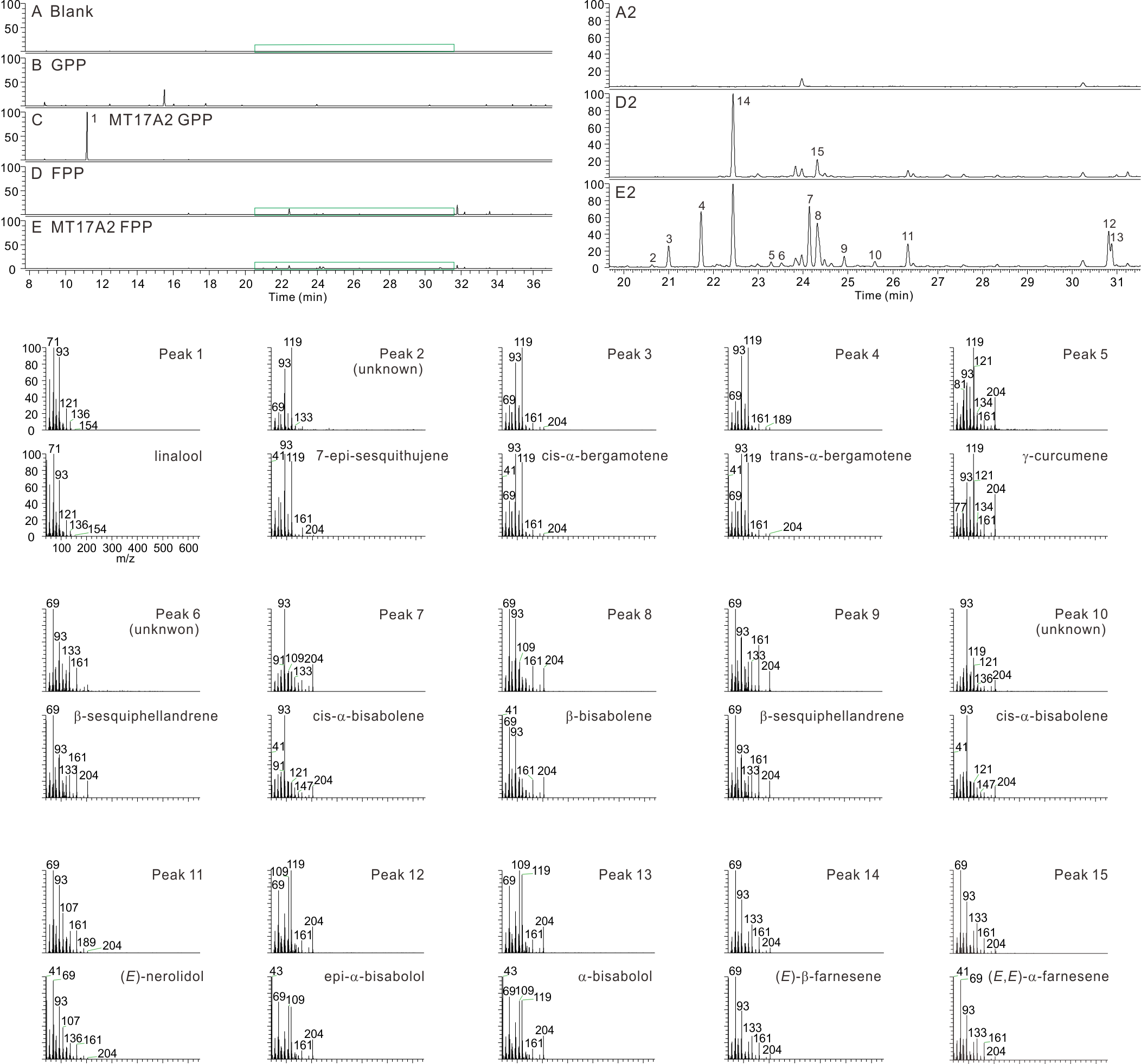

Supplement: Figure S14 — Analysis of MT17A2 function when protein was expressed in E. coli strain BL21 CodonPlus (DE3) RILP. Total ion chromatograms are displayed: pentane blank (A); enzyme assay using E. coli crude extract without pEXP5CT-MT17A2 plasmid with GPP (B) or FPP (D) as a substrate, respectively, or expressing MT17A2 with GPP (C) or FPP (E) as a substrate, respectively. A2, D2 and E2 are boxed regions from A, D and E panels to show very small peaks. Products/compounds identified include: 1,linalool; 2, unknown (7-epi-sesquithujene-like); 3, cis-α-bergamotene; 4, trans-α-bergamotene; 5, γ-curcumene; 6, unknown (β-sesquiphellandrene-like); 7, cis-α-bisabolene; 8, β-bisabolene; 9, β-sesquiphellandrene; 10, unknown (cis-α-bisabolene-like), 11, (E)-nerolidol; 12, epi-α-bisabolol; 13, α-bisabolol; 14, (E)-β-farnesene; 15, (E,E)-α-farnesene. (TIF) [file pone.0051481.s014.tif]

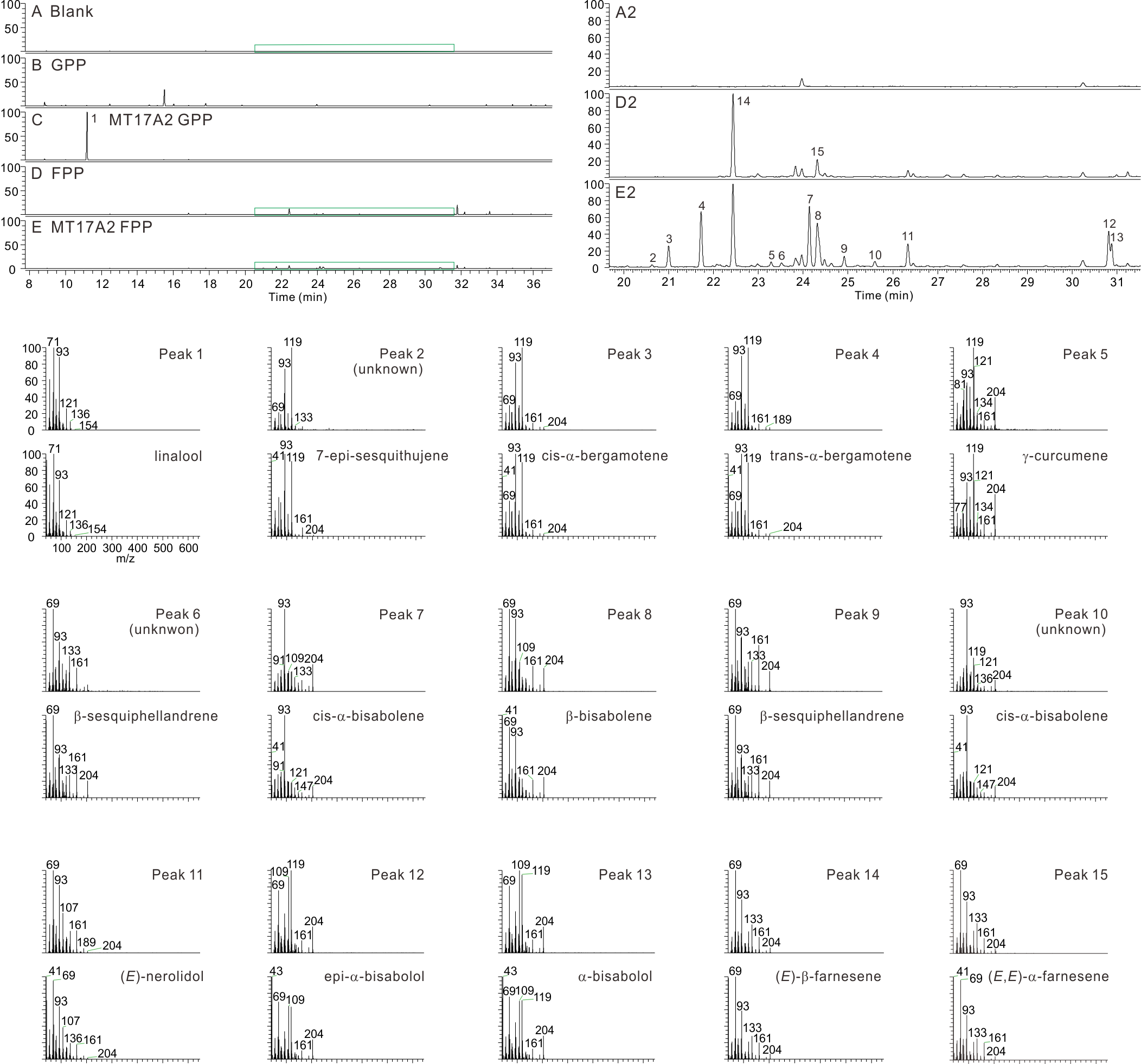

Supplement: Figure S15 — Analysis of MT06/MT06A functions when proteins were expressed in E. coli strain BL21 CodonPlus (DE3) RILP with GPP as a substrate. Total ion chromatograms are displayed: pentane blank (A); enzyme assay using E. coli crude extract without pEXP5CT-MT06 or pEXP5CT-MT06A plasmid with GPP as a substrate (B); enzyme assay using E. coli crude extract expressing either MT06 (C) or MT06A (D) with GPP as a substrate. Products/compounds identified include: 1, sabinene (4(10)-thujene); 2, α-terpinene; 3, γ-terpinene; 4, cis-sabinene hydrate; 5, p-mentha-1,4(8)-diene (terpinolene); 6, linalool; 7, p-menth-1-en-4-ol (terpinen-4-ol); 8, p-menth-1-en-8-ol (α-terpineol); 9, α-thujene (3-thujene); 10, myrcene. (TIF) [file pone.0051481.s015.tif]

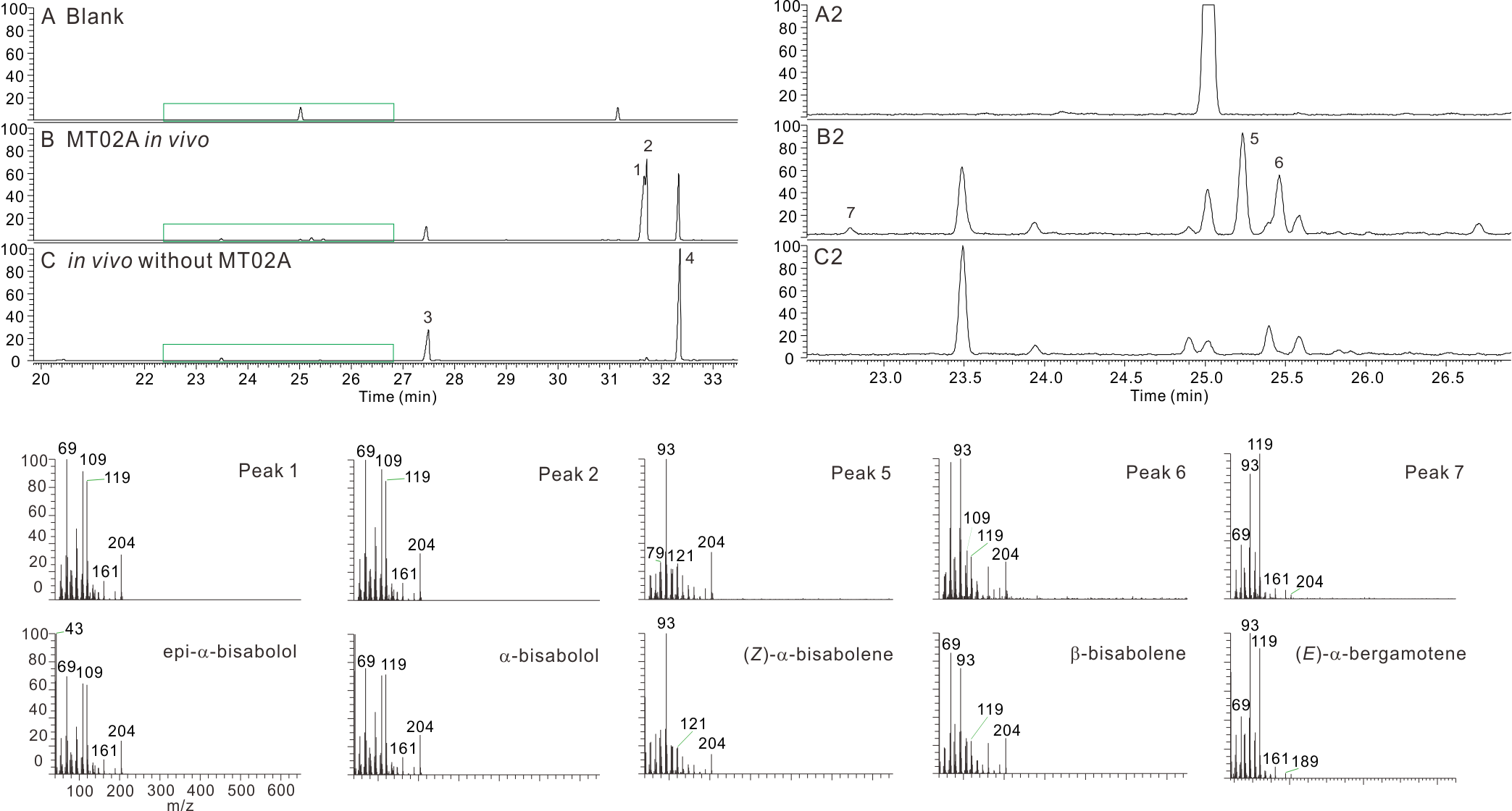

Supplement: Figure S16 — Analysis of MT02A function when protein was expressed in yeast strain EPY224. Total ion chromatograms are displayed: pentane blank (A); EPY224 with MT02A expression (B); EPY224 without the pESC-URA-MT02A plasmid (C). The boxed regions of A, B and C are enlarged in A2, B2 and C2 panels to show very small peaks. Products/compounds identified include: 1, epi-α-bisabolol; 2, α-bisabolol; 3, (E)-nerolidol; 4, farnesol; 5, (Z)-α-bisabolene; 6, β-bisabolene; 7, (E)-α-bergamotene. (TIF) [file pone.0051481.s016.tif]

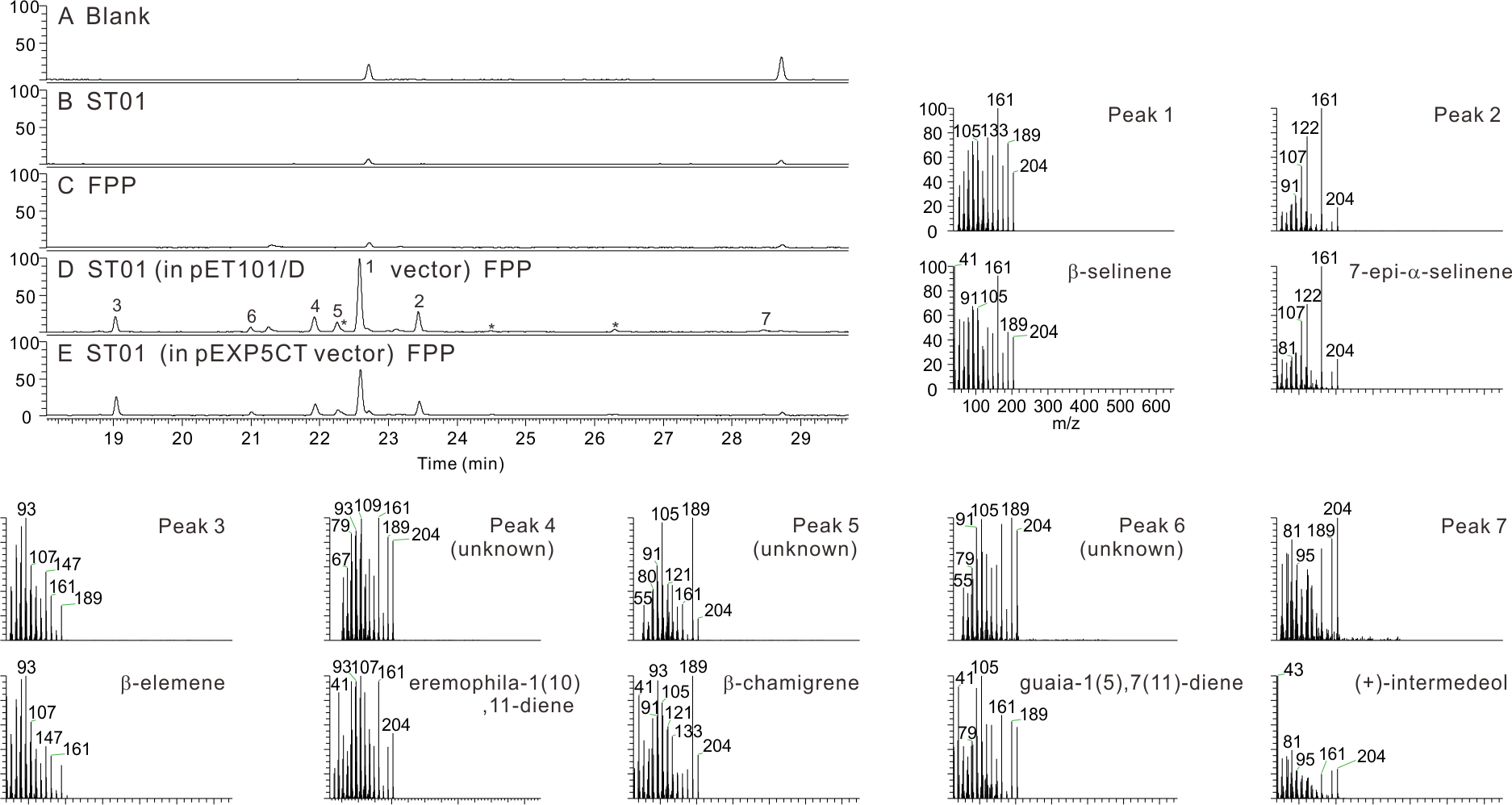

Supplement: Figure S17 — Analysis of ST01 function when protein was expressed in E. coli strain BL21 CodonPlus (DE3) RIL and BL21 CodonPlus (DE3) RILP. Total ion chromatograms are displayed: pentane blank (A); enzyme assay using E. coli (BL21 CodonPlus (DE3) RIL) crude extract expressing ST01 by pET101/D-ST01 plasmid without FPP (B) or with FPP as a substrate (D); enzyme assay using E. coli (BL21 CodonPlus (DE3) RILP) crude extract without either pET101/D-ST01 or pEXP5CT-ST01 plasmid with FPP as a substrate (C); enzyme assay using E. coli (BL21 CodonPlus (DE3) RILP) crude extract expressing ST01 by pEXP5CT-ST01 plasmid with FPP as a substrate (E). Products/compounds identified include: 1, β-selinene (eudesma-4(14),11-diene); 2, 7-epi-α-selinene; 3, β-elemene; 4, unknown (eremophila-1(10),11-diene-like); 5, unknown (β-chamigrene-like); 6, unknown (guaia-1(5),7(11)-diene-like); 7, (+)-intermedeol; *, unknown. (TIF) [file pone.0051481.s017.tif]

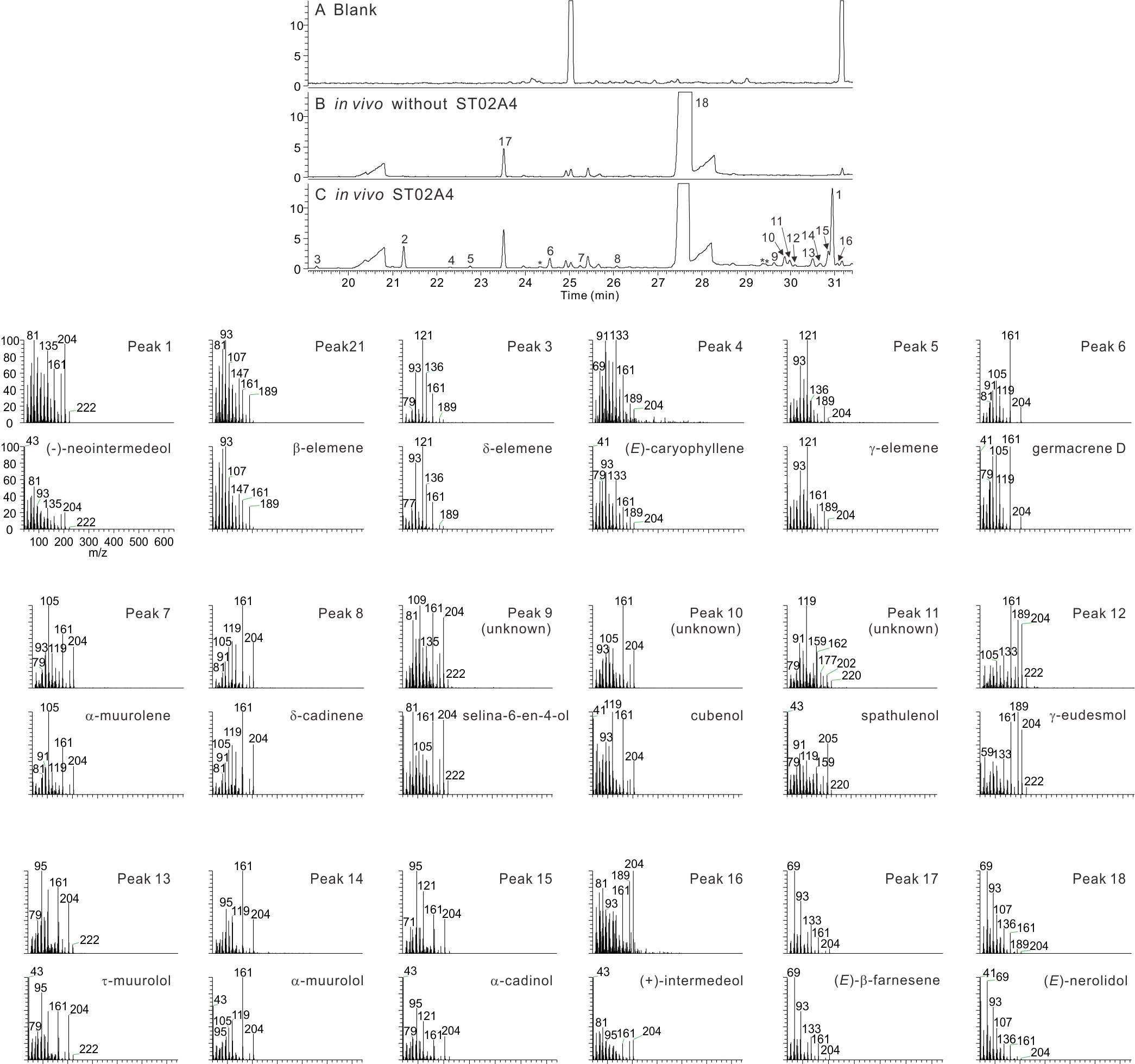

Supplement: Figure S18 — Analysis of ST02A4 function when protein was expressed in yeast strain EPY224. Total ion chromatograms are displayed: pentane blank (A); EPY224 without pESC-URA-ST02A4 plasmid (B); EPY224 expressing ST02A4 (C). Products/compounds identified include: 1, (−)-neointermedeol; 2, β-elemene; 3, δ-elemene; 4, (E)-caryophyllene; 5, γ-elemene; 6, germacrene D; 7, α-muurolene; 8, δ-cadinene (cadina-1(10),4-diene); 9, unknown (selina-6-en-4-ol-like); 10, unknown (cubenol-like); 11, unknown (spathulenol-like); 12, γ-eudesmol; 13, epi-α-muurolol (τ-muurolol); 14, α-muurolol (δ-cadinol); 15, α-cadinol; 16, (+)-intermedeol; 17, (E)-β-farnesene; 18, (E)-nerolidol; *, unknown. (TIF) [file pone.0051481.s018.tif]

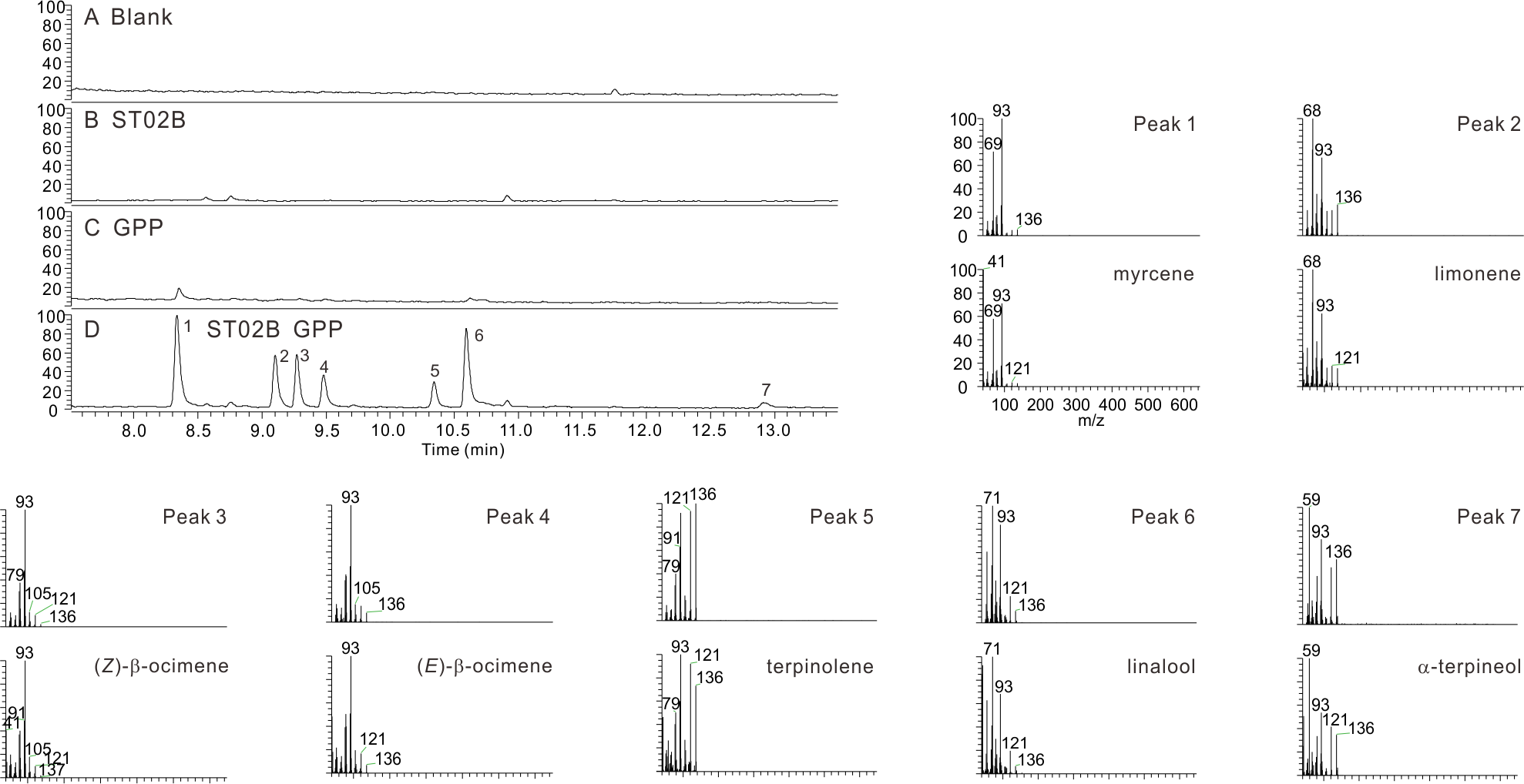

Supplement: Figure S19 — Analysis of ST02B function when protein was expressed in E. coli strain BL21 CodonPlus (DE3) RIL with GPP as a substrate. Total ion chromatograms are displayed: pentane blank (A); enzyme assay using E. coli crude extract expressing ST02B without GPP (B) or with GPP as a substrate (D); enzyme assay using E. coli crude extract without pET101/D-ST02B plasmid with GPP as a substrate (C). Products/compounds identified include: 1, myrcene; 2, limonene; 3, (Z)-β-ocimene; 4, (E)-β-ocimene; 5, p-mentha-1,4(8)-diene (terpinolene); 6, linalool; 7, p-menth-1-en-8-ol (α-terpineol). (TIF) [file pone.0051481.s019.tif]

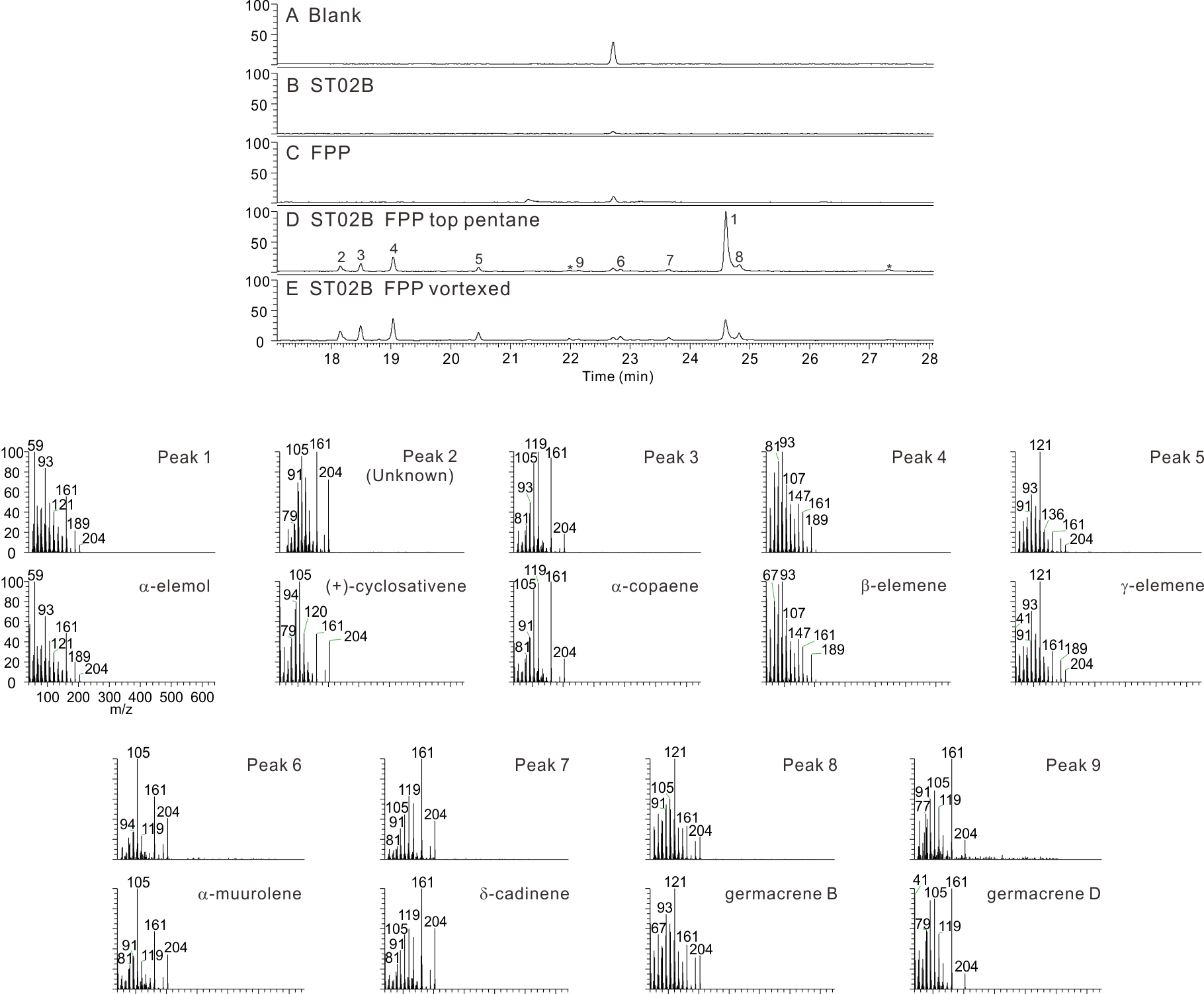

Supplement: Figure S20 — Analysis of ST02B function when protein was expressed in E. coli strain BL21 CodonPlus (DE3) RIL with FPP as a substrate. Total ion chromatograms are displayed: pentane blank (A); enzyme assay using E. coli crude extract expressing ST02B without FPP (B) or with FPP (D, E); enzyme assay using E. coli crude extract without pET101/D-ST02B plasmid with FPP (C). Enzyme assay was performed with pentane overlaid. After 3 hours of 30°C incubation, the top pentane layer was removed and directly injected into the GC/MS (D) or the whole enzyme assay including the top pentane was vortexed, centrifuged and the pentane phase collected and injected into the GC/MS (E). Products/compounds identified include: 1, α-elemol; 2, unknown ((+)-cyclosativene-like); 3, α-copaene; 4, β-elemene; 5, γ-elemene; 6, α-muurolene; 7, δ-cadinene (cadina-1(10),4-diene); 8, germacrene B; 9, germacrene D; *, unknown. (TIF) [file pone.0051481.s020.tif]

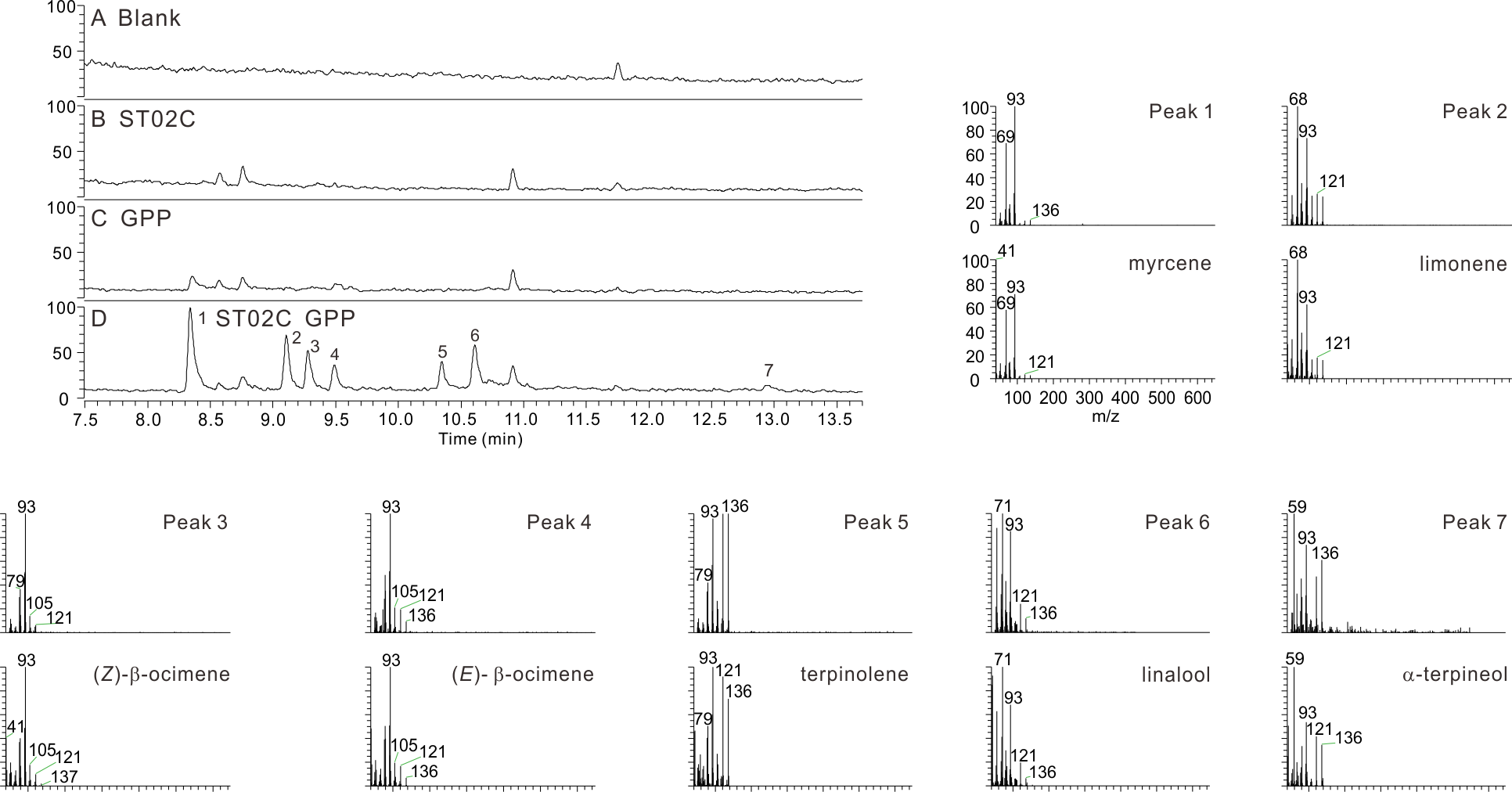

Supplement: Figure S21 — Analysis of ST02C function when protein was expressed in E. coli strain BL21 CodonPlus (DE3) RIL with GPP as a substrate. Total ion chromatograms are displayed: pentane blank (A); enzyme assay using E. coli crude extract expressing ST02C without GPP (B) or with GPP as a substrate (D), enzyme assay using E. coli crude extract without pET101/D-ST02C plasmid with GPP as a substrate (C). Products/compounds identified include: 1, myrcene; 2, limonene; 3, (Z)-β-ocimene; 4, (E)-β-ocimene; 5, p-mentha-1,4(8)-diene (terpinolene); 6, linalool; 7, p-menth-1-en-8-ol (α-terpineol). (TIF) [file pone.0051481.s021.tif]

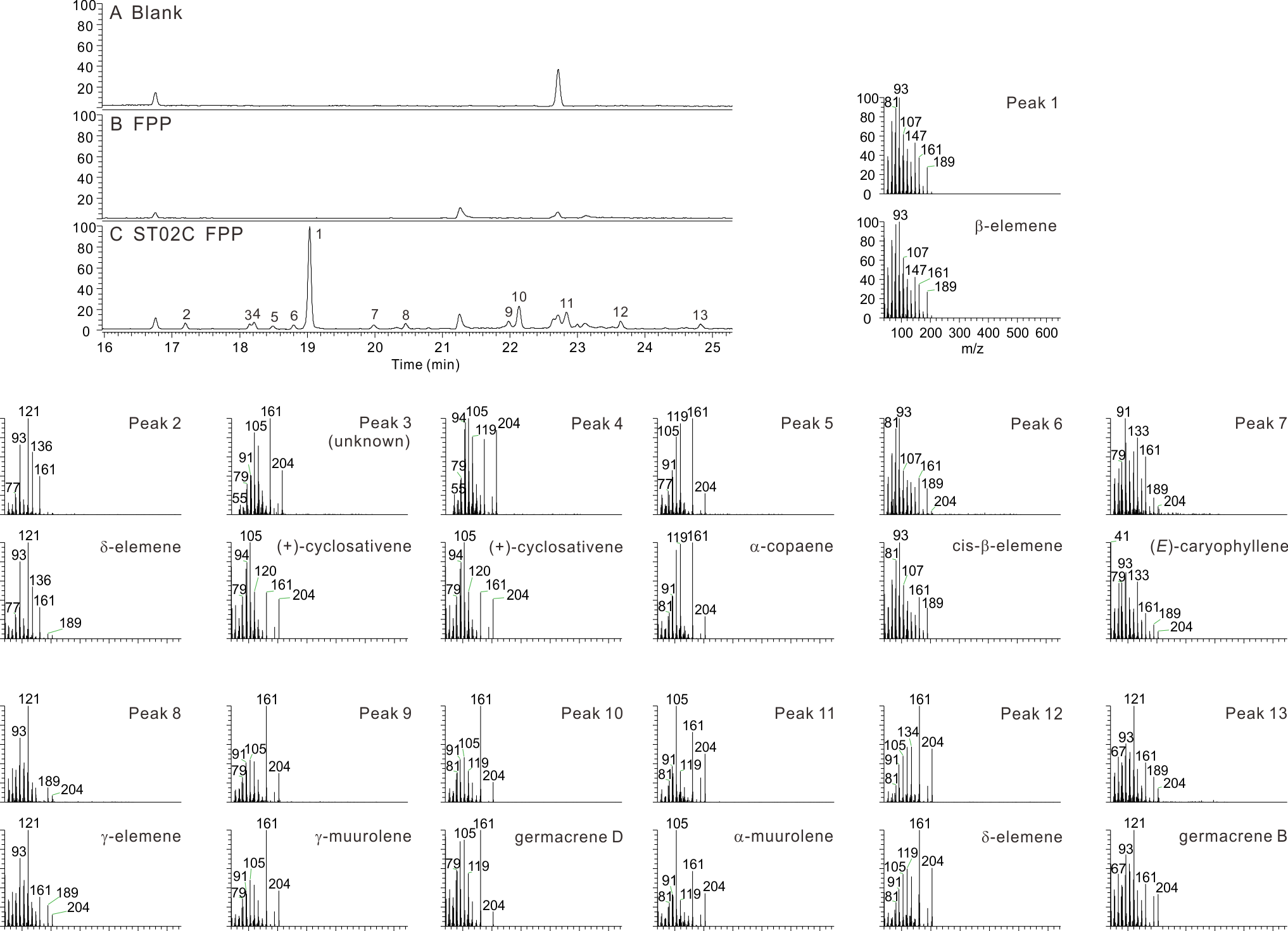

Supplement: Figure S22 — Analysis of ST02C function when protein was expressed in E. coli strain BL21 CodonPlus (DE3) RIL with FPP as a substrate. Total ion chromatograms are displayed: pentane blank (A); enzyme assay using E. coli crude extract without pET101/D-ST02C plasmid with FPP as a substrate (B); enzyme assay using E. coli crude extract expressing ST02C with FPP as a substrate (C). Products/compounds identified include: 1, β-elemene; 2, δ-elemene; 3, unknown ((+)-cyclosativene-like); 4, (+)-cyclosativene; 5, α-copaene; 6, cis-β-elemene; 7, (E)-caryophyllene; 8, γ-elemene; 9, γ-muurolene; 10, germacrene D; 11, α-muurolene; 12, δ-cadinene (cadina-1(10),4-diene); 13, germacrene B. (TIF) [file pone.0051481.s022.tif]

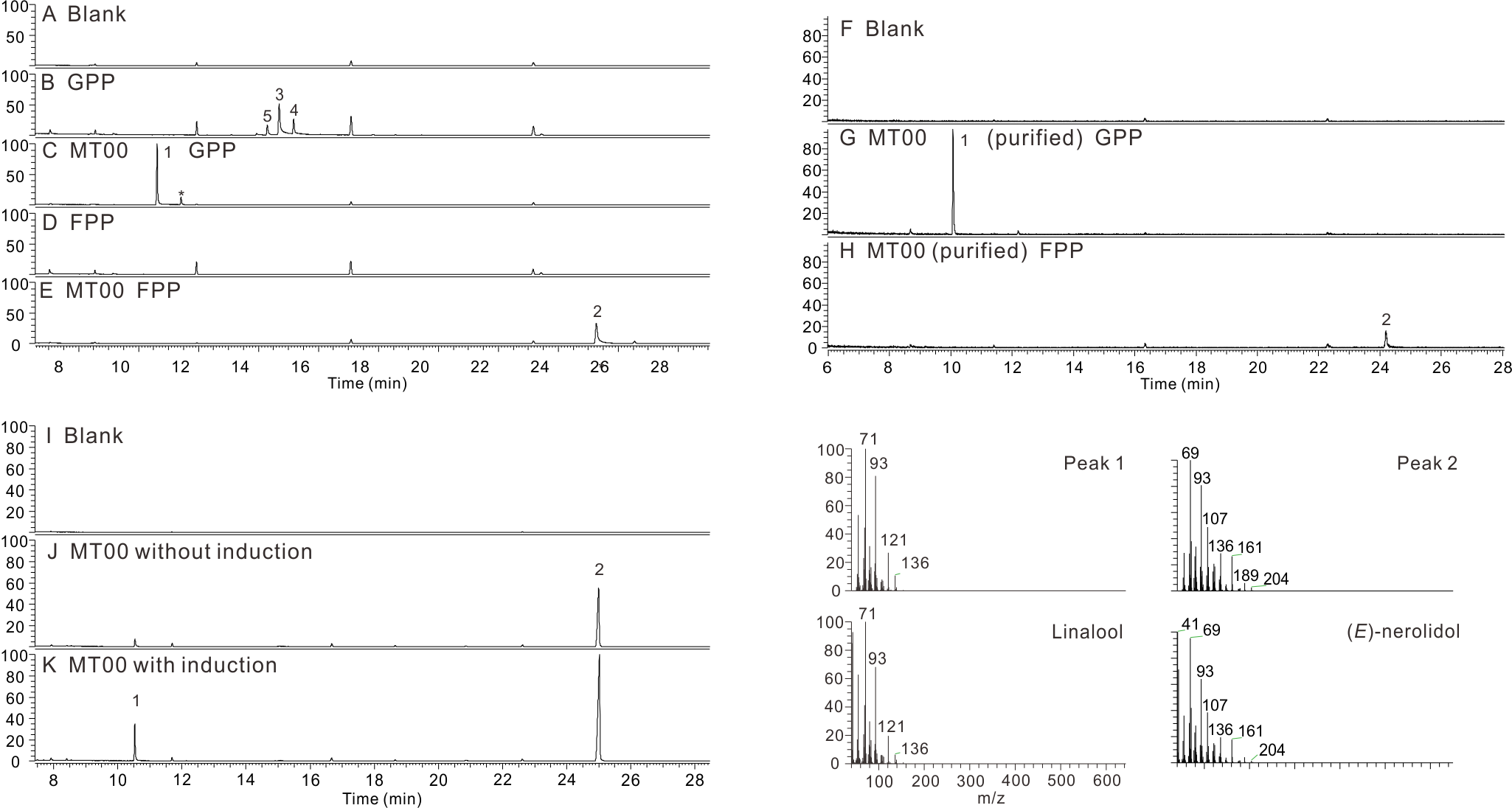

Supplement: Figure S23 — Analysis of ST03 function when protein was expressed in E. coli strain BL21 CodonPlus (DE3) RIL with FPP as a substrate. Total ion chromatograms are displayed: pentane blank (A); enzyme assay using E. coli crude extract expressing ST03 without FPP (B) or with FPP as a substrate (C). Products/compounds identified include: 1, γ-amorphene; 2, allo-aromadendrene; 3, γ-cadinene; 4, germacrene D-4-ol; 5, germacrene D. (TIF) [file pone.0051481.s023.tif]

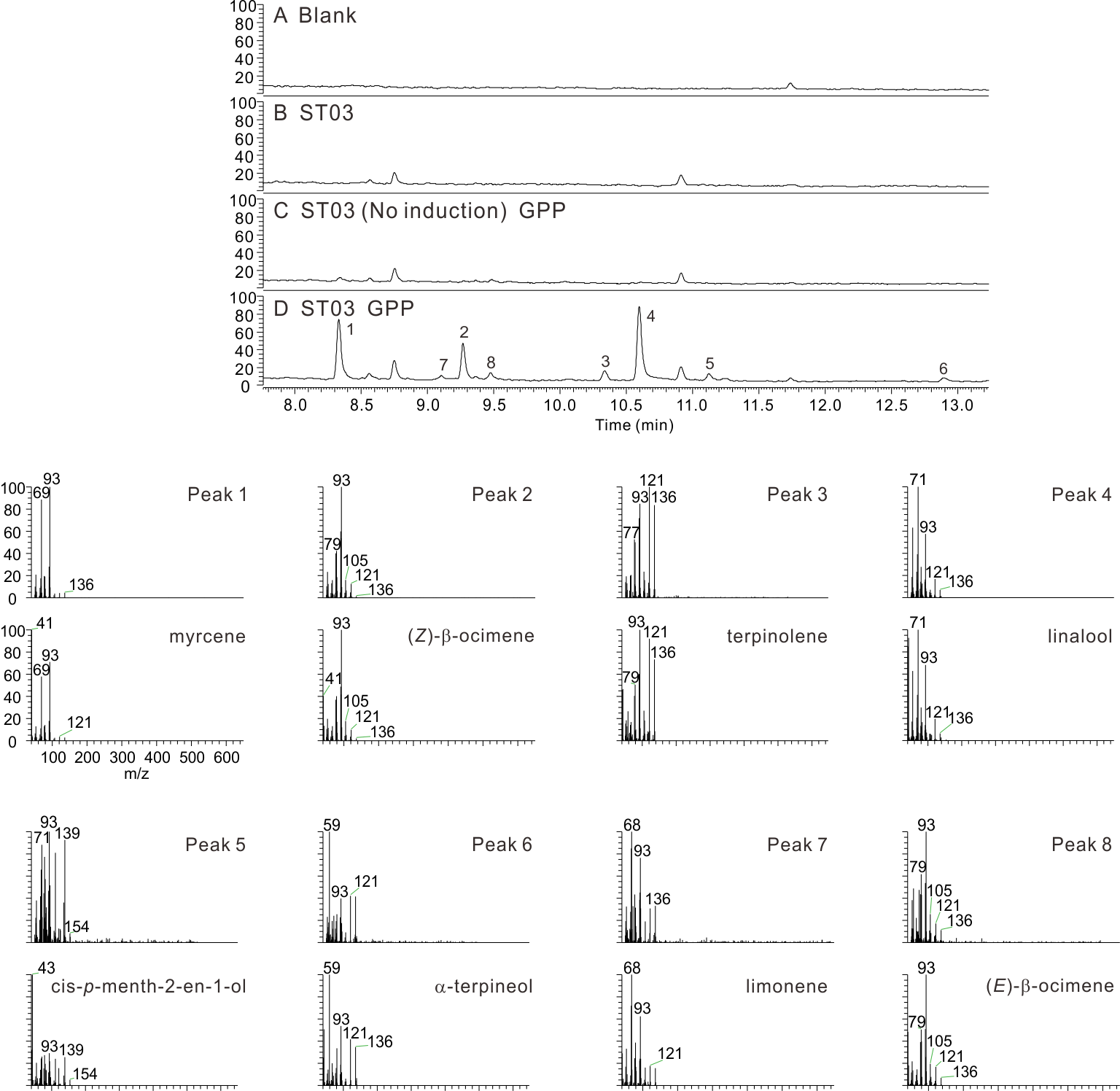

Supplement: Figure S24 — Analysis of ST03 functions with proteins expressed in E. coli strain BL21 CodonPlus (DE3) RIL and GPP as a substrate. Total ion chromatograms are displayed: pentane blank (A); enzyme assay using E. coli crude extract expressing ST03 without GPP (B) or with GPP as a substrate (D); enzyme assay with GPP as a substrate using E. coli crude extract containing pET101/D-ST03 plasmid but ST03 expression was not induced (C). pET101/D vector has lac operator and basal expression of ST03 is restricted. Products/compounds identified include: 1,myrcene; 2, (Z)-β-ocimene; 3, p-mentha-1,4(8)-diene (terpinolene); 4, linalool; 5, cis-p-menth-2-en-1-ol; 6, p-menth-1-en-8-ol (α-terpineol); 7, limonene; 8, (E)-β-ocimene. (TIF) [file pone.0051481.s024.tif]

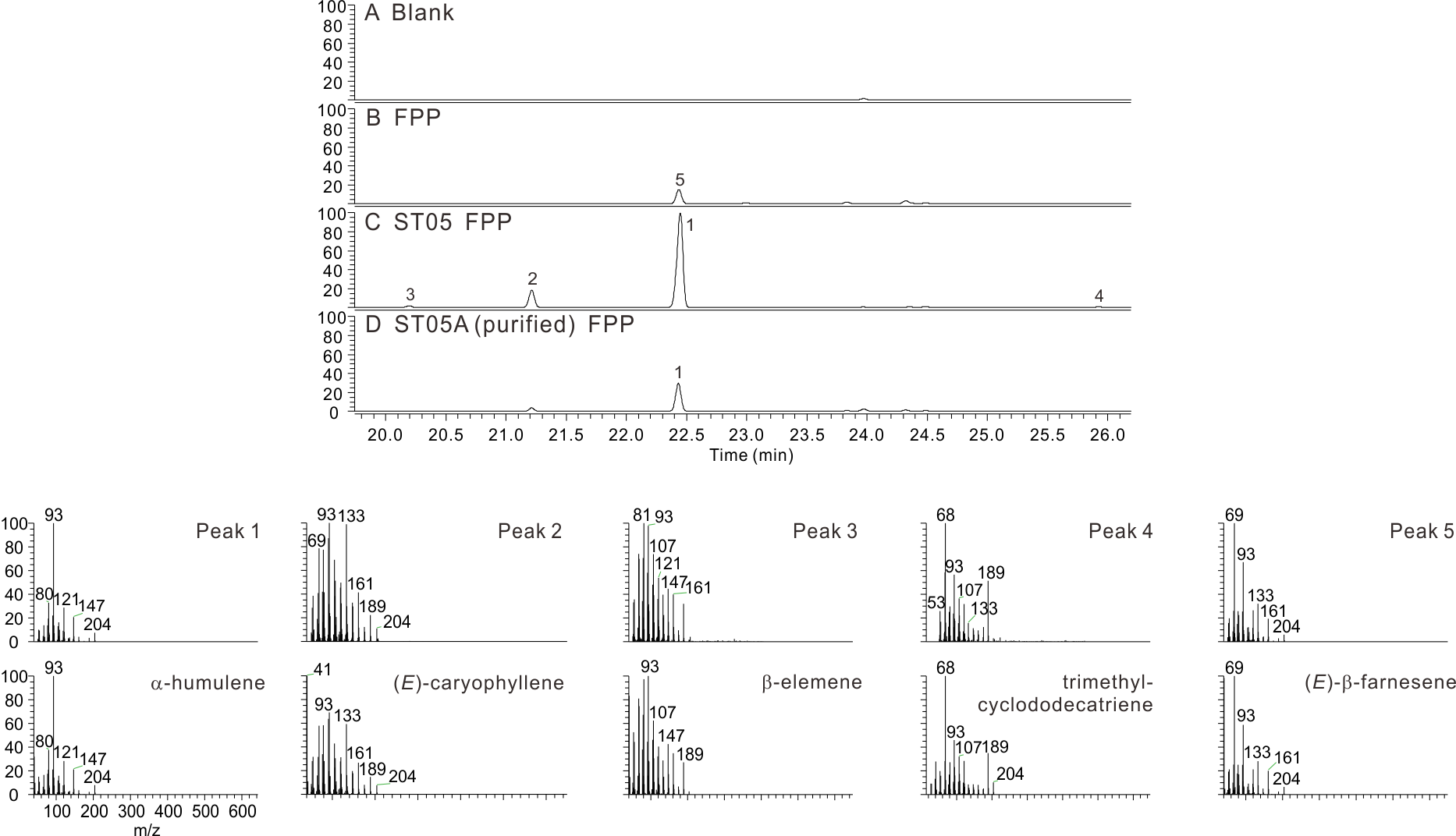

Supplement: Figure S25 — Analysis of ST05/ST05A functions when proteins were expressed in E. coli strain BL21 CodonPlus (DE3) RILP. Total ion chromatograms are displayed: pentane blank (A); enzyme assay with FPP as a substrate using E. coli crude extract without pEXP5CT-ST05 or pEXP5CT-ST05A plasmid (B); enzyme assay with FPP as a substrate using E. coli crude extract expressing ST05 (C); enzyme assay with FPP as a substrate using partially purified ST05A (D). Products/compounds identified include: 1, α-humulene (α-caryophyllene); 2, (E)-caryophyllene (β-caryophyllene); 3, β-elemene; 4, 1,5,9-trimethyl-1,5,9-cyclododecatriene; 5, (E)-β-farnesene. (TIF) [file pone.0051481.s025.tif]

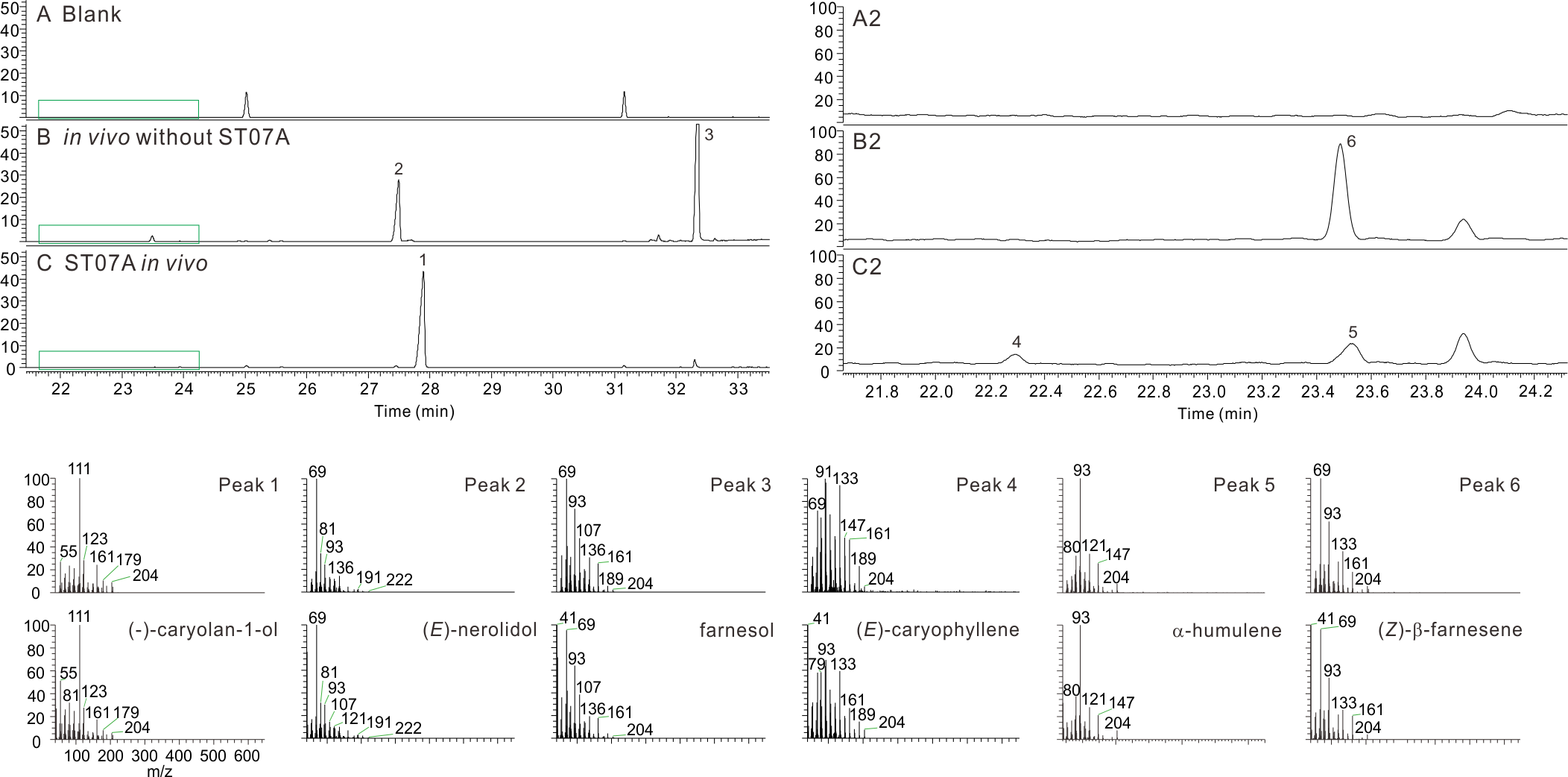

Supplement: Figure S26 — Analysis of ST07A function when protein was expressed in yeast strain EPY224. Total ion chromatograms are displayed: pentane blank (A); pentane extract of EPY224 without pESC-URA-ST07A plasmid (B); pentane extract of EPY224 expressing ST07A (C). The boxed regions of A, B and C are enlarged in A2, B2 and C2 panels to show very small peaks. Products/compounds identified include: 1, (−)-caryolan-1-ol; 2, (E)-nerolidol; 3, farnesol; 4, (E)-caryophyllene; 5, α-humulene; 6, (Z)-β-farnesene. (TIF) [file pone.0051481.s026.tif]

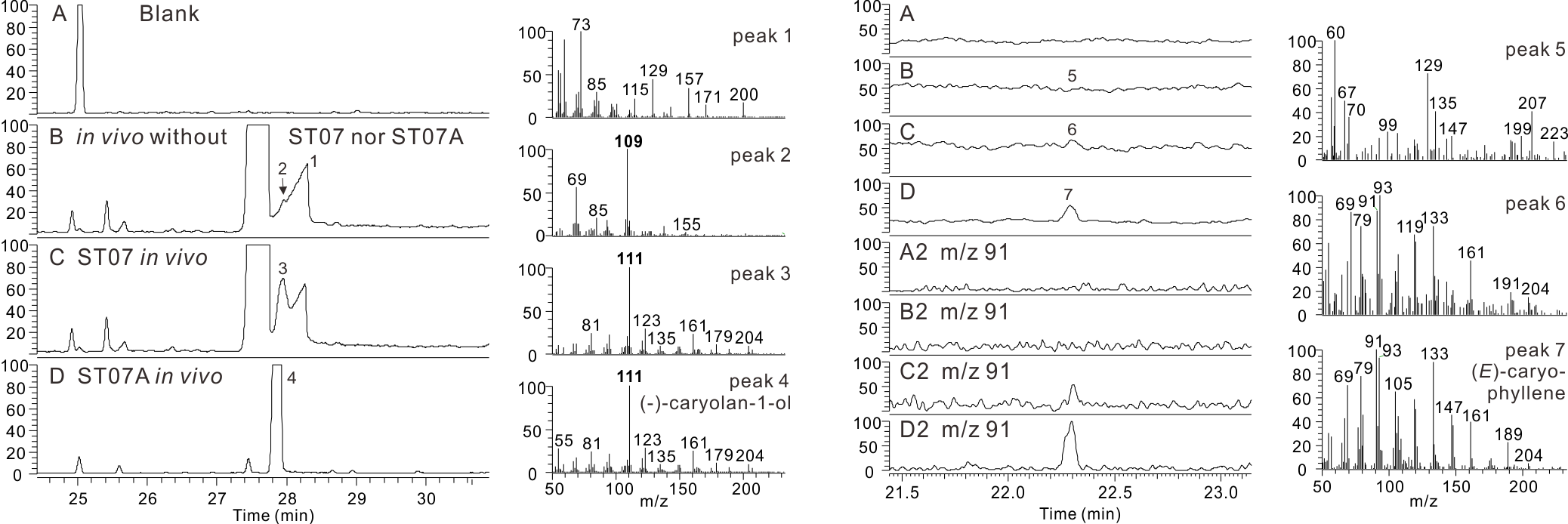

Supplement: Figure S27 — Analysis of ST07 function when protein was expressed in yeast strain EPY224. Total ion chromatograms are displayed: pentane blank (A); pentane extract of EPY219 without pESC-URA-ST07 plasmid (B); pentane extract of EPY219 expressing ST07 (C); pentane extract of EPY224 expressing ST07A (D). A2, B2, C2 and D2 are single ion chromatograms (m/z 91) of A, B, C and D. The most abundant ion in the (E)-caryophyllene mass spectrum is m/z 91. Products/compounds identified include: 1, 2 and 5, unknown; 3 and 4, (−)-caryolan-1-ol; 6 and 7, (E)-caryophyllene. (TIF) [file pone.0051481.s027.tif]
