# Supplementary material for: Suites of Terpene Synthases Explain Differential Terpenoid Production in Ginger and Turmeric Tissues
Source: PLoS One. 2012 Dec 18;7(12):e51481. doi: 10.1371/journal.pone.0051481 (PMC3525583; doi:10.1371/journal.pone.0051481)
Supplement: Table S1 — Primers for RACE. 5′ GSP, gene specific primers for 5′ RACE; 5′ N-GSP, nested gene specific primers for 5′ RACE; 3′ GSP, gene specific primers for 3′ RACE; 3′ N-GSP, nested gene specific primers for 3′ RACE. (DOC) [file pone.0051481.s028.doc]

**Table S1.** Primers for RACE.

5´ GSP, gene specific primers for 5´ RACE; 5´ N-GSP, nested gene specific primers for 5´ RACE; 3´ GSP, gene specific primers for 3´ RACE; 3´ N-GSP, nested gene specific primers for 3´ RACE

| Unitrans | Category | Primer name | Primer sequence (5´ -> 3´) |
| --- | --- | --- | --- |
| MT01 | 5´ GSP | MT12R | CAGATCAGAGGGCTTCTGAGTCTGTACG |
|  | 5´ N-GSP | MT01RR | CAGAGAAGCCATTCGTTCTC |
| MT02 | 5´ GSP | MT02R2 | TGCAACACCATCTCTCCCTCGGTTCCAT |
|  | 5´ N-GSP | MT02RR3 | TACAAACTCAGCATTCCTTC |
| MT04 | 5´ GSP | MT04R | ATCCGTCCACCATCTTGAGAGTTCAT |
|  | 5´ N-GSP | MT04RR | TCTCGCTTGTAGATGTTCTG |
| MT05 | 3´ GSP | MT05-3F | AAGGTGGCTTATGCCCTGGAACTGCCAT |
|  | 3´ N-GSP | MT05-3FF | ACAGAACTTGGAATCGCT |
|  | 5´ GSP | MT05R | CTCGGGAAGAACACCCAAGGAATGCTC |
|  | 5´ N-GSP | MT05RR | CATCTCTAAACTTGTAGAAAAT |
| MT06 | 5´ GSP | MT15R | GCCACGTCGCCTTTTTCTACCTCATCCG |
|  | 5´ N-GSP | MT06RR | ATCGTAAAGACGAGCAAGTG |
| MT07 | 3´ GSP | MT07-3F | GCATGCAGTAGTGGCGACGACACAATCA |
|  | 3´ N-GSP | MT07-3FF | ACTGGAGATGCAGAATGAAT |
|  | 5´ GSP | MT07R3 | GGTGGAACCATTTTGCTTCCTGAAAGCT |
|  | 5´ N-GSP | MT07RR2 | GTGCTGCACTCTCTCTAGCT |
| MT09 | 5´ GSP | MT09R | AGCAGCTTCAACTGATCGACCAACTGC |
|  | 5´ N-GSP | MT09RR2 | AGCGAGCTTCAAAGTTTCCA |
| MT12 | 3´ GSP | MT12-3F | AGCAGAGCAGCTTCAACTGATCGACCAA |
|  | 3´ N-GSP | MT12-3FF | GGCTTCTCTGTTTCACAAGGAAG |
|  | 5´ GSP | MT12R | CAGATCAGAGGGCTTCTGAGTCTGTACG |
|  | 5´ N-GSP | MT12RR | GTCTCTAAATGTCTCGAACCTT |
| MT15 | 5´ GSP | MT15R | GCCACGTCGCCTTTTTCTACCTCATCCG |
|  | 5´ N-GSP | MT15RR | GGAAGTGCCATAATCATCGAG |
| MT16 | 5´ GSP | MT16R | CGCCACTTTCTCTGAGACACCTTTTTCG |
|  | 5´ N-GSP | MT16RR | GAGCGTGTGATCGCATAGA |
| MT17 | 5´ GSP | MT17R | TACACCTCCAGTTGCCTCG |
|  | 5´ N-GSP | MT17RR | CTGCTCACGAGCCACCC |
| MT19 | 5´ GSP | MT19R | GTATGCCTGAGGAGCGTGTGATCGCATG |
|  | 5´ N-GSP | MT19RR | GCGTGTGATCGCATGGT |
| ST00 | 5´ GSP | Zd01L13RR | CGAGAACAACTAGGTTCATGAC |
| ST01 | 5´ GSP | ST01R2 | GATGGCACATATCGCTCCTCACCCCAC |
|  | 5´ N-GSP | ST01RR | TACCAAGAACCCAATAATAAGC |
| ST02 | 5´ GSP | Ca02L14GSP1 | TCTTTCCATGCATCCTCCACCATCTCC |
|  | 5´ N-GSP | Ca02L14RRR | TATTGTTGAAGCTGCATGCT |
| ST03 | 5´ GSP | Cb01O23GSP1 | TCCTTCCATGCGTCCTCCACCATCTCC |
|  | 5´ N-GSP | Cb01O23R4 | CTCTTTCATATAACATTCTACA |
| ST05 | 3´ GSP | ST05-3F | CGGCGCTAAGATTGGTTTATGGGGCTGA |
|  | 3´ N-GSP | ST05F | GAATGTGATGCGAAATGATACT |
|  | 5´ GSP | ST05R | CATTCCACTACTCGGTCACGAGCAAAC |
|  | 5´ N-GSP | ST05RR2 | CCACCACATTGAAATTTTCTTCACT |
| ST07 | 5´ GSP | ST07R | CCATGCTTGTTCCACCACCACTTGCAA |
|  | 5´ N-GSP | ST07RR | TTTCTCATGTGCCACTTTTTTG |
| ST09 | 5´ GSP | ST07R | CCATGCTTGTTCCACCACCACTTGCAA |
|  | 5´ N-GSP | ST09RR | TTTCTCATGTGCCACTTTTGTA |
