# Supplementary material for: Suites of Terpene Synthases Explain Differential Terpenoid Production in Ginger and Turmeric Tissues
Source: PLoS One. 2012 Dec 18;7(12):e51481. doi: 10.1371/journal.pone.0051481 (PMC3525583; doi:10.1371/journal.pone.0051481)
Supplement: Table S2 — Primers for cloning full length genes and sub-cloning for expression. Under the Category column, pCR2.1, pCRT7CT, pEXP5CT, pET101D and pENTRD mean pCR2.1-TOPO, pCRT7CT-TOPO, pEXP5CT-TOPO, pET101/D-TOPO and pENTR/D-TOPO vectors, respectively. Primers with F (forward) and R (reverse) suffix were used to amplify PCR fragments, which were then inserted in these vectors. PCR fragments with primers from category pDONR207 were produced by the Gateway BP reaction with the pDONR207 vector. PCR fragments with primers from category pESC-URA were sub-cloned into the pESC-URA vector. (DOC) [file pone.0051481.s029.doc]

**Table S2.** Primers for cloning full length genes and sub-cloning for expression.

Under the Category column, pCR2.1, pCRT7CT, pEXP5CT, pET101D and pENTRD mean pCR2.1-TOPO, pCRT7CT-TOPO, pEXP5CT-TOPO, pET101/D-TOPO and pENTR/D-TOPO vectors, respectively. Primers with F (forward) and R (reverse) suffix were used to amplify PCR fragments, which were then inserted in these vectors. PCR fragments with primers from category pDONR207 were produced by the Gateway BP reaction with the pDONR207 vector. PCR fragments with primers from category pESC-URA were sub-cloned into the pESC-URA vector.

| Unitrans | Category | Primer name | Primer sequence (5´ -> 3´) |
| --- | --- | --- | --- |
| MT00 | pDONR207-F | 10N21GtwyAttF | GGGGACAAGTTTGTACAAAAAAGCAGGCTTACTGGTTCCGCGTGGATCCATGCATTCATGCATGGTTCTC |
|  | pCRT7CT-F | Cb10N21CT-F | ATGAACCCTAGCAGTAATACCTC |
|  | pDONR207-R | 10N21G-AttR | GGGGACCACTTTGTACAAGAAAGCTGGGTCTGCCTATGATCGAGGCGTTA |
|  | pCRT7CT-R | Cb10N21CT-R | AATTTGTTCAAACAACAACA |
| MT01 | pCR2.1-F | MT01--F | ATGGCAACTGGTCAAGTTAT |
|  | pEXP5CT-F | MT01--Ft | ATGCTGCGTCGCTCGGGGAATTATCACCCAAG |
|  | pET101D-F | MT01--F-CACC | CACCATGCGTCGCTCGGGGAATTATCACCCAAG |
|  | pCR2.1-R, pEXP5CT-R, pET101D-R | MT01MT09--R | AATTTGGATAGGTTCGAATAAT |
| MT02 | pCR2.1-F | MT02--F | ATGTCTAGCTTCCTTCCTGCTCCACTAAATC |
|  | pEXP5CT-F | MT02--Ft | ATGAGCCGTCGCTCTGCCAACTTCCGGCCCAAC |
|  | pET101D-F | MT02--F-CACC | CACCATGCGTCGC TCTGCCAACTTCCGGCCCAACTTA |
|  | pESC-URA-F | MT02--Ft-AAAACA-*BamHI* | CGCGGATCCAAAACAATGCGTCGCTCTGCCAACTTCCGGCCCAACTTA |
|  | pCR2.1-R, pEXP5CT-R, pET101D-R | MT02--R | TTTTTCCACGACAATAGGGTTGACCAATAAT |
|  | pESC-URA-R | MT02--R-*XmaI* | TCCGCCCGGGCCTTTTTCCACGACAATAGGGTTGACCAATAAT |
| MT03 | pCR2.1-F | MT03--F | ATGTCGTCTATTTGCGCTCC |
|  | pEXP5CT-F | MT03--Ft | ATGCTGCGTCGCTTGGCGAATTATCATCCAAA |
|  | pCR2.1-R, pEXP5CT-R | MT03--R2 | GATTTGGATAGGTTCAAATACT |
| MT04 | pCR2.1-F | MT04--F | ATGTCTATCTCCCTTTCTTT |
|  | pEXP5CT-F | MT04--Ft | ATGCTGCGTCGCTCGGGGAATTACCAGCCCA |
|  | pET101D-F | MT04--F-CACC | CACCATGCGTCGCTCGGGGAATTACCAGCCCA |
|  | pCR2.1-R, pEXP5CT-R, pET101D-R | Zc07C01CT-R | GAGCTGGACAGGCTCGATCA |
| Unitrans | Category | Primer name | Primer sequence (5´ -> 3´) |
| MT05 | pCR2.1-F | MT05--F | ATGGCGCTCTTCCAACCTGC |
|  | pEXP5CT-F | MT05--Ft | ATGAGCCGTCGCTCGGCCAATTACCAGCCCA |
|  | pET101D-F | MT05--F-CACC | CACCATGCGTCGCTCGGCCAATTACCAGCCCA |
|  | pCR2.1-R, pEXP5CT-R, pET101D-R | MT05--R | CAATAGGATAGGGATGATCA |
| MT06 | pCR2.1-F | MT06--F | ATGGCTACTTGTCAAGCAAA |
|  | pEXP5CT-F | MT06--Ft | ATGCTGCGTCGCTCGGGGAATTATCATCCAAA |
|  | pET101D-F | MT06--F-CACC | CACCATGCGTCGCTCGGGGAATTATCATCCAAA |
|  | pCR2.1-R, pEXP5CT-R, pET101D-R | MT06--R | GATTTGGATAGGTTCGAATAAC |
| MT07 | pCR2.1-F | MT07--F | ATGGCTACAATGTCTATTTGCGCTCCC |
|  | pEXP5CT-F | MT07--Ft | ATGCTGCGTCGC TCAGGGAATTATCCGCCAAA |
|  | pET101D-F | MT07--F-CACC | CACCATGCGTCGC TCAGGGAATTATCCGCCAAACATATGG |
|  | pCR2.1-R, pEXP5CT-R, pET101D-R | MT15--R | GATTTGGATAGGTTCAAATAAC |
| MT08 | pENTRD-F | Zc05I02tFt | CTGGTTCCGCGTGGATCCATGGCTGACGTCGAGCAGTGTGA |
|  | pCRT7CT-F | MT08MT16--Ft | ATGCTGCGTCGCTCGGGGAATTATCAGCCAAG |
|  | pET101D-F | MT08MT16--F-CACC | CACCATGCGTCGC TCGGGGAATTATCAGCCAAG |
|  | pENTRD-R | Zc05I02tR | GTCATTCTAAATTTGGATAGGTTCG |
|  | pCR2.1-R, pCRT7CT-R, pET101D-R | Zc05I02CT-R | AATTTGGATAGGTTCGAGTA |
| MT09 | pCR2.1-F | MT08MT09MT16--F | ATGGCTACTCGTCAAGCAAT |
|  | pEXP5CT-F | MT09MT12--Ft | ATGCTGCGTCGC TCGGCGAATTATCATCCAAA |
|  | pET101D-F | MT09MT12--F-CACC | CACCATGCGTCGC TCGGCGAATTATCATCCAAA |
|  | pCR2.1-R, pEXP5CT-R, pET101D-R | MT01MT09--R | AATTTGGATAGGTTCGAATAAT |
| MT11 | pCRT7CT-F | Zc07C01CT-F | ATGAGGAGGTCGGGAAATTACCA |
|  | pCRT7CT-R (w/ stop, w/o His-tag) | Zc07C01tR | CGAGCCTGTCCAGCTCTGAT |
|  | pCRT7CT-R (w/o stop, w/ His-tag) | Zc07C01CT-R | GAGCTGGACAGGCTCGATCA |

| Unitrans | Category | Primer name | Primer sequence (5´ -> 3´) |
| --- | --- | --- | --- |
| MT12 | pCR2.1-F | MT12--F2 | ATGTCTATCTACTATACCAGCACTAC |
|  | pEXP5CT-F | MT09MT12--Ft | ATGCTGCGTCGC TCGGCGAATTATCATCCAAA |
|  | pET101D-F | MT09MT12--F-CACC | CACCATGCGTCGC TCGGCGAATTATCATCCAAA |
|  | pCR2.1-R, pEXP5CT-R, pET101D-R | MT01MT09--R | AATTTGGATAGGTTCGAATAAT |
| MT16 | pCR2.1-F | MT08MT09MT16--F | ATGGCTACTCGTCAAGCAAT |
|  | pEXP5CT-F | MT08MT16--Ft | ATGCTGCGTCGCTCGGGGAATTATCAGCCAAG |
|  | pET101D-F | MT08MT16--F-CACC | CACCATGCGTCGC TCGGGGAATTATCAGCCAAG |
|  | pESC-URA-F | MT08MT16--Ft-AAAACA-*BamHI* | CGCGGATCCAAAACAATGCTGCGTCGCTCGGGGAATTATCAGCCAAG |
|  | pCR2.1-R, pEXP5CT-R, pET101D-R | Zc05I02CT-R | AATTTGGATAGGTTCGAGTA |
|  | pESC-URA-R | MT08MT16--R-*XmaI* | TCCGCCCGGGCCAATTTGGATAGGTTCGAGTAAC |
| MT17 | pCR2.1-F | MT17--F | ATGCCCCCAGGCAATCCAGTTC |
|  | pEXP5CT-F | MT17--Ft | ATGGCGCGTCGCTCGGGCAATTACCAGCCCAAC |
|  | pET101D-F | MT17--F-CACC | CACCATGCGTCGCTCGGGCAATTACCAGCCCAAC |
|  | pCR2.1-R, pEXP5CT-R, pET101D-R | MT17--R | AATGATAGGGTTGATCAGCAGT |
| MT19 | pCR2.1-F | MT19--F | ATGTCTCTCTACTATACCAGCACTACTGTC |
|  | pEXP5CT-F | MT19--Ft | ATGCTGCGTCGCTCGGGGAATCATCAGCCAAG |
|  | pET101D-F | MT19--F-CACC | CACCATGCGTCGCTCGGGGAATCATCAGCC |
|  | pCR2.1-R, pEXP5CT-R, pET101D-R | MT19--R | GATTTGGATAGGTTCGAGTAAC |
| ST00 | pCR2.1-F, pEXP5CT-F | Zd01L13F | ATGGATCTTGACGAGACTCC |
|  | pET101D-F | Zd01L13F-CACC | CACCATGGATCTTGACGAGACTCC |
|  | pESC-URA-F | ST00--F-AAAACA-*BamHI* | CGCGGATCCAAAACAATGGATCTTGACGAGACTCC |
|  | pCR2.1-R, pEXP5CT-R, pET101D-R | Zd01L13R | AATAGGGATAGGATGAACAAGTAGA |
|  | pESC-URA-R | ST00--R-*XmaI* | TCCGCCCGGGCCAATAGGGATAGGATGAACAAGTAGA |
| ST01 | pCR2.1-F, pEXP5CT-F, pET101D-F | ST01--F-CACC | CACCATGGAGAAGCAATCACTAAC |
|  | pCR2.1-R, pEXP5CT-R, pET101D-R | ST01--R | TATAGGAACAGGTTCAACTAGCACCA |

| Unitrans | Category | Primer name | Primer sequence (5´ -> 3´) |
| --- | --- | --- | --- |
| ST02, ST03 | pCR2.1-F, pEXP5CT-F | ST02--F | ATGGAGAAGCAATCAACCAC |
|  | pET101D-F | ST02ST03--F-CACC | CACCATGGAGAAGCAATCAACCAC |
|  | pESC-URA-F | ST02--F-AAAACA-*BamHI* | CGCGGATCCAAAACAATGGAGAAGCAATCAACCAC |
|  | pCR2.1-R, pEXP5CT-R, pET101D-R | ST02ST03--R | AATAAGAACAGATTCAACCAACAACATAT |
|  | pESC-URA-R | ST02--R-*XmaI* | TCCGCCCGGGCCAATAAGAACAGATTCAACCAACAACATAT |
|  |  |  |  |
| ST05 | pCR2.1-F, pEXP5CT-F | ST05--F | ATGGAGAGGCAGTCGATGGC |
|  | pET101D-F | ST05--F-CACC | CACCATGGAGAGGCAGTCGATGGC |
|  | pCR2.1-R, pEXP5CT-R, pET101D-R | ST05--R | AATAGGAAAGGATTCAACCAATATAA |
| ST07, ST09 | pCR2.1-F, pEXP5CT-F | ST07--F | ATGGAGAGGCAGTCGATGGT |
|  | pET101D-F | ST07ST09--F-CACC | CACCATGGAGAGGCAGTCGATGGT |
|  | pESC-URA-F | ST07--F-AAAACA-*BamHI* | CGCGGATCCAAAACAATGGAGAGGCAGTCGATGGT |
|  | pCR2.1-R, pEXP5CT-R, pET101D-R | ST07ST09--R | AATAGGGAAGGATTCAACCAATATGA |
|  | pESC-URA-R | ST07--R-*XmaI* | TCCGCCCGGGCCAATAGGGAAGGATTCAACCAATATGA |
