# Supplementary material for: Suites of Terpene Synthases Explain Differential Terpenoid Production in Ginger and Turmeric Tissues
Source: PLoS One. 2012 Dec 18;7(12):e51481. doi: 10.1371/journal.pone.0051481 (PMC3525583; doi:10.1371/journal.pone.0051481)
Supplement: Table S3 — Mono- and sesquiterpene synthases identified in the ginger and turmeric EST database created from cDNA libraries from different tissues: Rh, rhizome; R, root; L, leaf. The numbers in the turmeric and ginger columns represent EST number per cDNA library in the database for the corresponding unitrans. Four unitrans, MT10, MT13, MT14 and MT18 were considered to belong to other unitrans after close investigation. In the RACE column, 5 or 3 means that 5′ or 3′ RACE was required to obtain full-length clones and, bolded and underlined means RACE was finished. Each unitrans was cloned for further characterization from the grey boxed sample. MT00 and MT11 were subcloned from the original cDNA clones directly without requiring RT-PCR. (DOC) [file pone.0051481.s030.doc]

**Table S3.** Mono- and sesquiterpene synthases identified in the ginger and turmeric EST database created from cDNA libraries from different tissues: Rh, rhizome; R, root; L, leaf.

The numbers in the turmeric and ginger columns represent EST number per cDNA library in the database for the corresponding unitrans. Four unitrans, MT10, MT13, MT14 and MT18 were considered to belong to other unitrans after close investigation. In the RACE column, 5 or 3 means that 5´ or 3´ RACE was required to obtain full-length clones and, **bolded and underlined** means RACE was finished. Each unitrans was cloned for further characterization from the grey boxed sample. MT00 and MT11 were subcloned from the original cDNA clones directly without requiring RT-PCR.

|  |  |  |  |  | |  | | Turmeric | |  |  | White ginger | | |  |  | Yellow ginger | | |  |
| --- | --- | --- | --- | --- | --- | --- | --- | --- | --- | --- | --- | --- | --- | --- | --- | --- | --- | --- | --- | --- |
| Unitrans | RACE | | | |  | | Rh | | L |  |  | Rh | R | L |  |  | Rh | R | L |  |
| MT00 |  |  |  |  | |  | |  | 2 |  |  |  |  |  |  |  |  |  |  |  |
| MT01 |  | **5** |  |  | |  | |  |  |  |  | 3 |  |  |  |  |  |  |  |  |
| MT02 |  | **5** |  |  | |  | | 2 |  |  |  |  |  |  |  |  |  |  |  |  |
| MT03 |  |  |  |  | |  | | 3 |  |  |  |  |  |  |  |  |  |  |  |  |
| MT04 |  | **5** |  |  | |  | |  |  |  |  |  | 5 |  |  |  |  | 2 |  |  |
| MT05 |  | **5** | **3** |  | |  | |  |  |  |  |  |  |  |  |  |  | 1 |  |  |
| MT06 |  | **5** |  |  | |  | |  |  |  |  |  |  |  |  |  | 4 |  |  |  |
| MT07 |  | **5** | **3** |  | |  | | 2 |  |  |  |  |  |  |  |  |  |  |  |  |
| MT08 |  |  |  |  | |  | |  |  |  |  | 1 |  |  |  |  | 1 |  |  |  |
| MT09 |  | **5** |  |  | |  | |  |  |  |  |  |  |  |  |  | 2 |  |  |  |
| MT10 (= MT03) |  | 5 | 3 |  | |  | | 2 |  |  |  |  |  |  |  |  |  |  |  |  |
| MT11 |  |  |  |  | |  | |  |  |  |  | 2 |  |  |  |  | 1 |  |  |  |
| MT12 |  | **5** | **3** |  | |  | |  |  |  |  |  | 2 |  |  |  |  |  |  |  |
| MT13 (= MT08) |  | 5 |  |  | |  | |  |  |  |  | 2 |  |  |  |  |  |  |  |  |
| MT14 (= MT02) |  | 5 | 3 |  | |  | | 2 |  |  |  |  |  |  |  |  |  |  |  |  |
| MT15 |  | 5 |  |  | |  | | 4 |  |  |  |  |  |  |  |  |  |  |  |  |
| MT16 |  | **5** |  |  | |  | |  |  |  |  |  |  |  |  |  | 2 |  |  |  |
| MT17 |  | **5** |  |  | |  | | 1 |  |  |  |  |  |  |  |  |  |  |  |  |
| MT18 (= MT09) |  | 5 |  |  | |  | |  |  |  |  |  | 2 |  |  |  |  |  |  |  |
| MT19 |  | **5** |  |  | |  | |  |  |  |  |  | 2 |  |  |  |  |  |  |  |
| ST00 |  |  |  |  | |  | | 5 |  |  |  |  |  |  |  |  |  |  |  |  |
| ST01 |  | **5** |  |  | |  | |  |  |  |  |  |  |  |  |  | 2 |  |  |  |
| ST02 |  | **5** |  |  | |  | |  |  |  |  |  |  |  |  |  | 2 |  |  |  |
| ST03 |  | **5** |  |  | |  | |  | 2 |  |  |  |  |  |  |  |  |  |  |  |
| ST04 |  | 5 |  |  | |  | |  |  |  |  |  |  |  |  |  |  | 2 |  |  |
| ST05 |  | **5** | **3** |  | |  | |  |  |  |  |  |  |  |  |  |  | 4 |  |  |
| ST06 |  | 5 |  |  | |  | |  |  |  |  |  |  |  |  |  |  | 2 |  |  |
| ST07 |  | **5** |  |  | |  | |  |  |  |  | 2 |  |  |  |  |  |  |  |  |
| ST08 |  | 5 |  |  | |  | |  |  |  |  | 2 |  |  |  |  |  |  |  |  |
| ST09 |  | **5** |  |  | |  | |  |  |  |  |  | 1 |  |  |  |  |  |  |  |
