# Supplementary material for: Suites of Terpene Synthases Explain Differential Terpenoid Production in Ginger and Turmeric Tissues
Source: PLoS One. 2012 Dec 18;7(12):e51481. doi: 10.1371/journal.pone.0051481 (PMC3525583; doi:10.1371/journal.pone.0051481)
Supplement: Table S4 — Expression levels of ginger and turmeric terpene synthase unitrans based on microarray data. Probes for microarrays were designed from partial sequences of unitrans before RACEs revealed full sequences. Abbreviations are; GY, Yellow Ginger; F, turmeric variety Fat Mild Orange (FMO); T, turmeric variety Thin Yellow Aromatic (TYA); Rh, rhizome; R, root; L, leaf. TYA barely produces sesquiterpenes and is used as a control for microarray experiments. The chemical profiles of the FMO variety have no differences from the turmeric variety, Hawaiian Red Turmeric (HRT) used to clone genes. Both were clonally derived from the same original line. (DOC) [file pone.0051481.s031.doc]

**Table S4.** Expression levels of ginger and turmeric terpene synthase unitrans based on microarray data.

Probes for microarrays were designed from partial sequences of unitrans before RACEs revealed full sequences. Abbreviations are; GY, Yellow Ginger; F, turmeric variety Fat Mild Orange (FMO); T, turmeric variety Thin Yellow Aromatic (TYA); Rh, rhizome; R, root; L, leaf. TYA barely produces sesquiterpenes and is used as a control for microarray experiments. The chemical profiles of the FMO variety have no differences from the turmeric variety, Hawaiian Red Turmeric (HRT) used to clone genes. Both were clonally derived from the same original line.

|  | GY-Rh | | | | | GY-R | | GY-L | | FMO-Rh | | | F-R | F-L | TYA-Rh | | | T-R | T-L |
| --- | --- | --- | --- | --- | --- | --- | --- | --- | --- | --- | --- | --- | --- | --- | --- | --- | --- | --- | --- |
| Unitrans | 2M | 3M | 4M | 6M | 7M | 2M | 7M | 2M | 7M | 3M | 5M | 7M | 7M | 7M | 3M | 5M | 7M | 7M | 7M |
| MT00 | 28 | 41 | 37 | 37 | 51 | 33 | 37 | 63 | 61 | 487 | 380 | 229 | 21 | 17 | 41 | 16 | 39 | 31 | 25 |
| MT01 | 28670 | 23361 | 23602 | 28327 | 25260 | 5238 | 3592 | 472 | 458 | 14 | 13 | 11 | 8 | 9 | 14 | 7 | 8 | 11 | 7 |
| MT02 | 32 | 37 | 52 | 32 | 76 | 25 | 26 | 27 | 68 | 30 | 104 | 42 | 141 | 96 | 14 | 14 | 12 | 526 | 13 |
| MT04 | 1339 | 1657 | 2457 | 1142 | 871 | 6586 | 928 | 148 | 104 | 8 | 8 | 13 | 13 | 13 | 8 | 13 | 11 | 10 | 9 |
| MT05 | 62 | 67 | 74 | 48 | 101 | 454 | 202 | 46 | 318 | 180 | 499 | 40 | 14 | 15 | 29 | 34 | 60 | 90 | 54 |
| MT06 | 4730 | 5059 | 7973 | 7345 | 10864 | 296 | 1833 | 148 | 143 | 27 | 43 | 23 | 34 | 18 | 15 | 23 | 26 | 16 | 22 |
| MT07 | 34 | 33 | 38 | 27 | 35 | 42 | 18 | 17 | 25 | 925 | 549 | 42 | 8 | 10 | 15 | 4 | 14 | 8 | 9 |
| MT08 | 63 | 53 | 51 | 51 | 75 | 51 | 68 | 69 | 62 | 17 | 28 | 29 | 48 | 27 | 26 | 53 | 31 | 16 | 26 |
| MT09 | 12828 | 13413 | 16413 | 25254 | 23809 | 7267 | 8313 | 589 | 814 | 35 | 25 | 13 | 18 | 32 | 32 | 21 | 19 | 79 | 17 |
| MT10 MT03 | 9610 | 10486 | 14145 | 18409 | 18788 | 13208 | 8830 | 1105 | 884 | 10409 | 5600 | 550 | 158 | 35 | 61 | 29 | 97 | 292 | 185 |
| MT11 | 2390 | 2937 | 3966 | 2036 | 1445 | 70 | 102 | 87 | 72 | 9 | 10 | 6 | 4 | 2 | 6 | 4 | 15 | 6 | 2 |
| MT12 | 2153 | 4200 | 6331 | 4100 | 5352 | 4065 | 1164 | 147 | 103 | 36 | 225 | 17 | 5 | 12 | 25 | 15 | 109 | 58 | 39 |
| MT13 MT08 | 1736 | 1721 | 2759 | 3143 | 3444 | 101 | 66 | 84 | 78 | 6 | 10 | 8 | 10 | 8 | 7 | 10 | 8 | 7 | 8 |
| MT14 MT02 | 58 | 53 | 51 | 55 | 49 | 51 | 60 | 48 | 55 | 6 | 117 | 25 | 28 | 36 | 16 | 35 | 17 | 109 | 18 |
| MT15 | 90 | 92 | 84 | 119 | 99 | 100 | 139 | 96 | 134 | 8780 | 1653 | 232 | 29 | 22 | 24 | 22 | 23 | 14 | 21 |
| MT16 | 4212 | 1809 | 4578 | 4147 | 5375 | 1043 | 1089 | 135 | 81 | 10 | 8 | 8 | 10 | 9 | 7 | 9 | 9 | 11 | 6 |
| MT17 | 105 | 139 | 124 | 135 | 143 | 157 | 469 | 101 | 160 | 361 | 250 | 87 | 710 | 59 | 53 | 53 | 38 | 45 | 48 |
| MT18 MT09 | 10585 | 9390 | 11698 | 11928 | 9781 | 2261 | 903 | 154 | 139 | 16 | 6 | 11 | 12 | 13 | 5 | 7 | 9 | 11 | 13 |
| MT19 | 601 | 188 | 645 | 339 | 411 | 6692 | 548 | 70 | 27 | 16 | 10 | 9 | 6 | 9 | 10 | 5 | 8 | 7 | 8 |
| ST00 | 486 | 557 | 1305 | 343 | 696 | 52 | 43 | 35 | 31 | 30404 | 25043 | 19601 | 3126 | 29 | 71 | 64 | 128 | 65 | 9 |
| ST01 | 461 | 466 | 662 | 514 | 514 | 83 | 61 | 25 | 46 | 9 | 9 | 12 | 12 | 10 | 9 | 10 | 14 | 12 | 15 |
| ST02 | 11615 | 7340 | 11705 | 18083 | 14874 | 622 | 2344 | 115 | 97 | 17 | 16 | 11 | 10 | 349 | 13 | 9 | 10 | 9 | 260 |
| ST03 | 28393 | 26107 | 28205 | 43085 | 32871 | 2684 | 3322 | 180 | 85 | 4 | 12 | 8 | 2 | 5 | 6 | 8 | 5 | 3 | 6 |
| ST04 | 249 | 313 | 362 | 254 | 343 | 516 | 99 | 37 | 36 | 14 | 18 | 18 | 9 | 13 | 12 | 8 | 12 | 13 | 9 |
| ST05 | 126 | 226 | 196 | 187 | 184 | 510 | 268 | 96 | 163 | 140 | 284 | 61 | 35 | 51 | 206 | 647 | 938 | 520 | 534 |
| ST06 | 43326 | 33248 | 38497 | 46103 | 39529 | 2132 | 6064 | 482 | 325 | 7 | 18 | 30 | 9 | 5 | 5 | 5 | 7 | 9 | 6 |
| ST07 | 1062 | 1529 | 832 | 485 | 727 | 4301 | 3498 | 269 | 3778 | 21 | 16 | 15 | 11 | 25 | 87 | 46 | 84 | 111 | 40 |
| ST08 | 42292 | 35770 | 38132 | 44583 | 41407 | 1912 | 8214 | 453 | 257 | 4 | 5 | 8 | 8 | 8 | 6 | 7 | 8 | 5 | 13 |
| ST09 | 193 | 217 | 78 | 140 | 67 | 726 | 196 | 22 | 26 | 12 | 10 | 11 | 7 | 10 | 50 | 38 | 46 | 25 | 44 |
