# Supplementary material for: Suites of Terpene Synthases Explain Differential Terpenoid Production in Ginger and Turmeric Tissues
Source: PLoS One. 2012 Dec 18;7(12):e51481. doi: 10.1371/journal.pone.0051481 (PMC3525583; doi:10.1371/journal.pone.0051481)
Supplement: Table S5 — Vectors used to express various ginger or turmeric TPS proteins in either E. coli or yeast cells. Solubility of expression in E. coli was checked in Coomassie-stained gels. Solubility of expression in yeast was checked by Western blotting. Solubility is the ratio of total and soluble fractions. n/a means that the solubility was not evaluated. The vector and cell combinations marked with "*" were used for further analysis to identify the functions of specific proteins as outlined in the text. (DOC) [file pone.0051481.s032.doc]

**Table S5.** Vectors used to express various ginger or turmeric TPS proteins in either *E. coli* or yeast cells.

Solubility of expression in *E. coli* was checked in Coomassie-stained gels. Solubility of expression in yeast was checked by Western blotting. Solubility is the ratio of total and soluble fractions. n/a means that the solubility was not evaluated. The vector and cell combinations marked with "*" were used for further analysis to identify the functions of specific proteins as outlined in the text.

|  | **Expression in *E. coli*** | | | | **Expression in yeast** | | | |
| --- | --- | --- | --- | --- | --- | --- | --- | --- |
| **Gene name** | **vectors** | **cells** | **Solubility** | **vectors** | | **cells** | **Solubility** |  |
| MT00 | pH9GW | BL21 (DE3) pLysS | +++ |  | |  |  |  |
|  |  | Rosetta (DE3) | ++++ |  | |  |  |  |
|  |  | Rosetta2 (DE3) pLysS* | +++ |  | |  |  |  |
|  | pCRT7CT | BL21 (DE3) pLysS | +++ |  | |  |  |  |
|  |  | BL21 CodonPlus (DE3) RIL | ++++ |  | |  |  |  |
|  |  | BL21 CodonPlus (DE3) RP | +++ |  | |  |  |  |
|  |  | BL21 Star (DE3) pMevT pMBI RIL* | ++++ |  | |  |  |  |
| MT01 | pH9GW | BL21 CodonPlus (DE3) RILP | - |  | |  |  |  |
| MT02A | pEXP5CT | BL21 CodonPlus (DE3) RILP | - | pESC-URA | | EPY219 | n/a |  |
|  |  | Rosetta2 (DE3) pLysS | - |  | | EPY224* | +++++ |  |
| MT03 | pET101D | BL21 CodonPlus (DE3) RIL* | +++++ |  | |  |  |  |
|  | pEXP5CT | BL21 CodonPlus (DE3) RILP | +++++ |  | |  |  |  |
| MT04 | pEXP5CT | BL21 CodonPlus (DE3) RILP* | +++++ |  | |  |  |  |
| MT05 | pEXP5CT | BL21 CodonPlus (DE3) RILP | - |  | |  |  |  |
| MT06 | pEXP5CT | BL21 CodonPlus (DE3) RILP* | ++++ |  | |  |  |  |
| MT06A | pEXP5CT | BL21 CodonPlus (DE3) RILP* | +++ |  | |  |  |  |
| MT06B | pEXP5CT | BL21 CodonPlus (DE3) RILP* | ++ |  | |  |  |  |
| MT07 | pEXP5CT | BL21 CodonPlus (DE3) RILP* | +++++ |  | |  |  |  |
| MT08 | pH9GW | BL21 (DE3) pLysS | - |  | |  |  |  |
|  |  | BL21 CodonPlus (DE3) RIL | - |  | |  |  |  |
|  |  | BL21 Star (DE3) pMevT pMBI RIL* | + |  | |  |  |  |
|  |  | Rosetta (DE3) | - |  | |  |  |  |
|  |  | Rosetta2 (DE3) pLysS | - |  | |  |  |  |
|  | pCRT7CT | BL21 (DE3) pLysS | - |  | |  |  |  |
|  |  | BL21 CodonPlus (DE3) RIL | + |  | |  |  |  |
|  |  | BL21 CodonPlus (DE3) RP | - |  | |  |  |  |
|  |  | BL21 Star (DE3) | - |  | |  |  |  |
|  |  | BL21 Star (DE3) RIL | + |  | |  |  |  |
|  |  | BL21 Star (DE3) pMevT pMBI RIL | - |  | |  |  |  |
|  |  | Rosetta2 (DE3) pLysS | + |  | |  |  |  |
|  |  | BL21-AI | - |  | |  |  |  |
|  |  | BL21-AI RIL | - |  | |  |  |  |
|  |  | ArcticExpress (DE3) RIL | + |  | |  |  |  |
|  | pEXP5CT | BL21 CodonPlus (DE3) RILP | - |  | |  |  |  |
| MT09A | pEXP5CT | BL21 CodonPlus (DE3) RILP | - |  | |  |  |  |
| MT09A2 | pEXP5CT | BL21 CodonPlus (DE3) RILP* | ++ |  | |  |  |  |
| MT09B | pEXP5CT | BL21 CodonPlus (DE3) RILP | ++ |  | |  |  |  |
|  | **Expression in *E. coli*** | | | | **Expression in yeast** | | | |
| **Gene name** | **vectors** | **cells** | **Solubility** | **vectors** | | **cells** | **Solubility** |  |
| MT11 | pH9GW | BL21 (DE3) pLysS | - |  | |  |  |  |
|  |  | BL21 CodonPlus (DE3) RIL | - |  | |  |  |  |
|  |  | BL21 CodonPlus (DE3) RP | - |  | |  |  |  |
|  |  | Rosetta (DE3) | - |  | |  |  |  |
|  |  | Rosetta2 (DE3) pLysS | - |  | |  |  |  |
|  | pCRT7CT | BL21 (DE3) pLysS | - |  | |  |  |  |
|  |  | BL21 CodonPlus (DE3) RIL* | ++ |  | |  |  |  |
|  |  | BL21 CodonPlus (DE3) RP | - |  | |  |  |  |
|  |  | BL21 Star (DE3) pMevT pMBI RIL* | + |  | |  |  |  |
|  |  | Rosetta (DE3) | - |  | |  |  |  |
| MT12A-M2 | pEXP5CT | BL21 CodonPlus (DE3) RILP* | ++++ |  | |  |  |  |
| MT16 | pEXP5CT | BL21 CodonPlus (DE3) RILP | - | pESC-URA | | EPY219 | - |  |
|  |  | Rosetta2 (DE3) pLysS | +++++ |  | |  |  |  |
| MT17A2 | pEXP5CT | BL21 CodonPlus (DE3) RILP* | +++ |  | |  |  |  |
| MT17C | pEXP5CT | BL21 CodonPlus (DE3) RILP | - |  | |  |  |  |
| MT17D | pEXP5CT | BL21 CodonPlus (DE3) RILP | - |  | |  |  |  |
| MT19 | pEXP5CT | BL21 CodonPlus (DE3) RILP | +++++ |  | |  |  |  |
|  |  | Rosetta2 (DE3) pLysS | +++++ |  | |  |  |  |
| ST00A | pH9GW | BL21 CodonPlus (DE3) RILP | +++ | pESC-URA | | EPY219* | +++++ |  |
|  |  | BL21-AI RIL* | +++ |  | |  |  |  |
|  |  | Rosetta2 (DE3) pLysS | +++ |  | |  |  |  |
| ST00B | pH9GW | BL21 CodonPlus (DE3) RILP | +++ | pESC-URA | | EPY219* | n/a |  |
|  |  | BL21-AI RIL* | +++ |  | |  |  |  |
|  |  | Rosetta2 (DE3) pLysS | +++ |  | |  |  |  |
| ST01 | pET101D | BL21 CodonPlus (DE3) RIL* | ++ |  | |  |  |  |
|  | pEXP5CT | BL21 CodonPlus (DE3) RILP* | + |  | |  |  |  |
| ST02A | pEXP5CT | BL21 CodonPlus (DE3) RILP | - | pESC-URA | | EPY219 | - |  |
|  |  | Rosetta2 (DE3) pLysS | - |  | |  |  |  |
|  |  | ArcticExpress (DE3) RIL | - |  | |  |  |  |
| ST02A2 | pEXP5CT | BL21 CodonPlus (DE3) RILP | - | pESC-URA | | EPY219 | - |  |
| ST02A3 | pEXP5CT | BL21 CodonPlus (DE3) RILP | - | pESC-URA | | EPY219 | - |  |
| ST02A4 | pEXP5CT | BL21 CodonPlus (DE3) RILP | - | pESC-URA | | EPY219 | - |  |
|  |  |  |  |  | | EPY224* | n/a |  |
| ST02B | pET101D | BL21 CodonPlus (DE3) RIL* | +++++ |  | |  |  |  |
| ST02C | pET101D | BL21 CodonPlus (DE3) RIL* | +++++ |  | |  |  |  |
| ST02C2 | pET101D | BL21 CodonPlus (DE3) RIL | - |  | |  |  |  |
|  | pEXP5CT | BL21 CodonPlus (DE3) RILP | - |  | |  |  |  |
| ST03 | pET101D | BL21 CodonPlus (DE3) RIL* | +++++ |  | |  |  |  |
|  | pEXP5CT | BL21 CodonPlus (DE3) RILP | +++ |  | |  |  |  |
| ST05 | pEXP5CT | BL21 CodonPlus (DE3) RILP* | +++++ |  | |  |  |  |
| ST05A | pEXP5CT | BL21 CodonPlus (DE3) RILP* | + |  | |  |  |  |
| ST07 | pEXP5CT | BL21 CodonPlus (DE3) RILP | - | pESC-URA | | EPY219 | - |  |
|  |  | Rosetta2 (DE3) pLysS | - |  | | EPY224* | n/a |  |
|  |  | ArcticExpress (DE3) RIL | - |  | |  |  |  |
| ST07A | pEXP5CT | BL21 CodonPlus (DE3) RILP | - | pESC-URA | | EPY224* | +++++ |  |
