# Supplementary material for: Suites of Terpene Synthases Explain Differential Terpenoid Production in Ginger and Turmeric Tissues
Source: PLoS One. 2012 Dec 18;7(12):e51481. doi: 10.1371/journal.pone.0051481 (PMC3525583; doi:10.1371/journal.pone.0051481)
Supplement: Results S1 — Additional details regarding cloning, expression and characterization of specific TPS genes can be found here. (DOCX) [file pone.0051481.s033.docx]

**Cloning of terpene synthases**

From ginger and turmeric cDNA libraries, 20 unitrans of monoterpenes, 10 unitrans of sesquiterpenes, 2 unitrans of diterpenes, 3 unitrans of triterpenes and 10 unitrans of tetraterpenes were found by homology search to known terpene synthases and we focused on mono- and sesquiterpene synthases. Four monoterpene synthase unitrans and 3 sesquiterpene synthase unitrans were removed from the list because they were very similar to other unitrans and had very few polymorphisms or they had some extra sequences not related to terpene synthases. Out of the remaining 16 monoterpene synthases and 7 sesquiterpene synthases, 2 monoterpene synthases (MT00 and MT11) were full length, 2 monoterpenes synthases (MT03 and MT05) were full length but had frameshifts, and the others were partial at 5´ and/or 3´ end. Nine monoterpene synthase unitrans were missing only the 5´end sequences and 3 monoterpene unitrans required both 5´ and 3´ end sequences. For sesquiterpene synthases, 6 unitrans needed 5´ end sequences and 1 unitrans needed both 5´ and 3´ end sequences (Table S3). Using 5´and 3´ RACE (Rapid Amplification of cDNA Ends), we could identify unknown regions of most unitrans except for one monoterpene synthase, MT15, which is missing the 5´ end sequence and had high homology with MT06, as shown in a phylogenetic tree (Figure 2), which was generated with ginger and turmeric terpene synthases (25 cloned MTPSs, 18 cloned STPSs and 15 unitrans for di-, tri- and tetra-terpene synthases) and 181 plant terpene synthases from GenBank. After the phylogenetic tree was generated, 181 plant terpene synthases are removed from the phylogenetic tree and only ginger and turmeric terpene synthases are shown, for clarity sake. Even though most unitrans were partial, similar terpene synthases were grouped together. The sesquiterpene synthase clade was separated from other terpene synthase groups, which was also separated into mono-, di-, tri-, and tetra-terpene synthase clades. Although MT00 is considered to be a monoterpene synthase, it resides in an outgroup from the monoterpene synthase clade.

Two unitrans, MT00 and MT11 have full length ESTs without frameshifts and were sub-cloned from the EST plasmid directly into the cloning vector. For other incomplete unitrans, we cloned full length genes from ginger or turmeric tissues (rhizome, root or leaf). Several unitrans, as shown in Table S3, were cloned from tissues different from those where their EST(s) was/were reported. In the beginning, we tried to clone genes from RACE ready cDNA derived from the tissue that had EST(s) for the corresponding unitrans. Some genes were hard to clone from those tissues and we tried RACE ready cDNAs from other tissues than the tissue from which the EST(s) was/were found. After that, we first checked if the RACE ready cDNA from each tissue had the gene or not using PCR with two gene specific primers before we started RACE. White ginger and yellow ginger are very similar and rhizome and root tissues also show similar metabolite profiles. MT09 has ESTs in the GY-Rh library and MT18, which is also considered the same as MT09, has ESTs in the GW-R library. So it is not surprising that even though ST05 was cloned from GW-R cDNA, ESTs were found in the GY-R sample and ST07 was cloned from the GW-R cDNA when ESTs were found in GW-Rh. MT17 was cloned from the T-L sample, although ESTs were found in T-Rh. Moreover, ST03 was cloned from the GY-Rh sample, although ESTs was found in the T-L sample.

ST06 and ST08 unitrans are highly homologous with ST00 and RACE was performed only for ST00. After RACE of ST09, ST09 was not cloned because ST07 and ST09 are considered to be paralogs. Therefore, ST06, ST08 and ST09 are not described in the expression part of this report.

**Expression of terpene synthases**

All chromatograms in the figures except Figures 1, 4 and 7 have absolute scales. During identification of products using the GC/MS library, there are sometimes unknown peaks where it is hard to determine which compound they represent. They are called “unknown” because the score from the library comparison is not high enough or there is no information about retention time and it could not be compared accurately with other compounds, in which case the most similar compound is marked with the term “-like”. An example for this process is shown in Figure 25E.

**α-Zingiberene/β-sesquiphellandrene synthase (ST00A and ST00B)**

ST00A and ST00B genes are very similar, with 98.8% homology at the DNA sequence level.

Enzyme assays using *E. coli* crude extracts from cells expressing either ST00A or ST00B with GPP as a substrate showed β-phellandrene (49.2%) and α-pinene (33.7%) as major products and sabinene (4(10)-thujene) (6.2%), β-pinene (5.9%) and α-phellandrene (5.0%) as minor products although their amounts are very small (Table 1, Figure S3). With FPP as a substrate, ST00A and ST00B produced α-zingiberene (49.3%), β-sesquiphellandrene (40.7%), β-bisabolene (6.3%) and an unknown (trans-sesquisabinene hydrate-like2) compound (3.7%) (Figure S3).

Expression of ST00A and ST00B in the yeast strain EPY219 at lower temperature, 18 °C, yielded more products than expression at 30 °C, and longer induction times (8 days versus 2 days) normally yielded more products as well. After 4 days of induction at 18 °C, pentane extracts of EPY219 expressing ST00A were analyzed; α-zingiberene (67%), β-sesquiphellandrene (22.7%), β-bisabolene (6.2%), *ar*-curcumene (0.9%), unknown (trans-sesquisabinene hydrate-like2) (0.6%), (*E*)-γ-bisabolene (0.4%), unknown (trans-sesquisabinene hydrate-like3) (0.4%), γ-eudesmol (0.4%), γ-curcumene (0.3%), unknown (7-epi-sesquithujene-like) (0.3%), unknown (α-eudesmol-like) (0.3%), trans-α-bergamotene (0.2%) and α-acorenol (0.2%) (Table 2, Figure 3, Figure S4). When ST00A was expressed in INVSc1 yeast cells, we also could see α-zingiberene and β-sesquiphellandrene peaks, however, the amounts are 2.56% and 2.34%, respectively, when compared to the amount from EPY224 expressing ST00A. When considering the peak area of (*E*)-nerolidol that is produced by yeast itself in both INVSc1 and EPY224 as a standard, the produced α-zingiberene/β-sesquiphellandrene in the INVSc1 expressing ST00A is only 3.33% of that from EPY224 expressing ST00A and the yield difference is mainly due to overall ability of IPP production in these yeast strains.

There was a difference in the ratio of α-zingiberene and β-sesquiphellandrene in *E. coli* and yeast expressions. The amount of α-zingiberene in yeast is about 3 times more than the amount of β-sesquiphellandrene, however, only 1.2 times more in *E. coli*. When expressed in *E. coli*, we did not include the stop codon when we insert ST00A or ST00B to pH9GW vector, which caused extra amino acids to be added at the 3´ end. Also, there is a His-tag at the 5´ end of the protein expressed from the pH9GW vector. For yeast, translation starts at ATG of ST00A or ST00B and there is a short c-Myc tag at the 3´ end. EPY219 produces the products of ST00A or ST00B *in vivo*, whereas we used *in vitro* enzyme assays to measure activities of these enzymes expressed in *E. coli*. Therefore, the product ratio from yeast is more reliable than *E. coli* for ST00A or ST00B and used for Table 1 and Table 2.

**β-Phellandrene synthase (MT08)**

From EST data, the sequence of MT08 is complete but has one frameshift, so MT08 with the frameshift eliminated was cloned from white ginger rhizome. However, MT08 proteins expressed in *E. coli* with/without the transit peptide were insoluble when the pCR7CT-TOPO vector was used for expression and did not show any terpene product in enzyme assays with GPP or FPP. MT08 without transit peptide plus thrombin cleavage site at the N terminus was cloned into pH9GW vector and expressed in BL21 Star (DE3) pMevT pMBI RIL. MT08 expressed in BL21 Star (DE3) pMevT pMBI RIL produced β-phellandrene and trace amounts of α-pinene and (*Z*)-β-farnesene. However, the peaks were small because of low solubility. Enzyme assays of *E. coli* crude extracts with GPP yielded more terpene products and the α-pinene peak was clearly visible at this point; β-phellandrene (88.3%), α-pinene (11.7%) (Table 2, Figure S5).

The microarray probe designed for MT08 did not show any expression pattern (Table S4). However, MT13 represents the same gene as MT08 (Table S3). Therefore, microarray data from MT13 (=MT08) can be used for MT08.

**Camphene/α-pinene synthases (MT06B, MT09A2 and MT12A-M2)**

Camphene is the most abundant monoterpene in ginger, and three genes, MT06B, MT09A2 and MT12A-M2 produce camphene as a major product. MT12A-M2 has one point mutation, D140G, when compared to MT12A.

With GPP as a substrate, MT06B synthesized camphene (45.4%) and α-pinene (40.4%) as major products and limonene (6.6%), borneol (endo-borneol) (3.3%), *p*-mentha-1,4(8)-diene (terpinolene) (1.5%), β-pinene (1.0%), tricyclene (0.7%), cis-sabinene hydrate (0.4%), *p*-menth-1-en-8-ol (α-terpineol) (0.3%) and (*E*)-pinene hydrate ((*E*)-pinan-2-ol) (0.3%) as minor products (Table 1, Figure S6). MT09A2 utilizes GPP and produced camphene (60.1%) and α-pinene (20.4%) as major products and limonene (7.7%), borneol (endo-borneol) (6.1%), *p*-mentha-1,4(8)-diene (terpinolene) (1.6%), tricyclene (1.5%), β-citronellal (0.9%), β-pinene (0.6%), 8, cis-sabinene hydrate (0.4%), *p*-menth-1-en-8-ol (α-terpineol) (0.4%), (*E*)-pinene hydrate ((*E*)-pinan-2-ol) (0.3%) and γ-terpinene (0.1%) as minor products (Table 1, Figure S7). With GPP as substrate, MT12A-M2 synthesized camphene (55.8%) and α-pinene (28.4%) as major products and limonene (7.9%), borneol (endo-borneol) (3.3%), *p*-mentha-1,4(8)-diene (terpinolene) (1.9%), tricyclene (1.2%), β-pinene (0.9%), *p*-menth-1-en-8-ol (α-terpineol) (0.4%), (*E*)-pinene hydrate ((*E*)-pinan-2-ol) (0.3%) and cis-sabinene hydrate (0.1%) as minor products (Table 1, Figure S8). All three proteins did not produce sesquiterpenes with FPP as substrate.

The ratio of camphene/α-pinene is different in these proteins; 1.12 for MT06B, 2.95 for MT09A2 and 1.96 for MT12A-M2 (Table 1). In 7 month old ginger rhizome samples, the ratio of camphene/α-pinene is 2.99, which suggests that MT09A2 or related genes (MT09, MT09B, etc.) are expressed at higher levels than the other TPS genes, although it is also possible that there are other genes that make camphene more specifically. Microarray data support this hypothesis: the expression levels of MT06, MT09 and MT12 unitrans in 7 month old yellow ginger rhizome are 10864, 23809 and 5352 respectively (Table S4).

**α-Pinene/β-pinene synthase (MT04)**

MT06, MT09A2 and MT06B produce more camphene than α-pinene, however, MT04 mainly produces α-pinene. Enzyme assays yielded α-pinene (60.1%) and β-pinene (30.7%) as major products and limonene (5.6%), sabinene (4(10)-thujene) (2.7%) and 1,8-cineole (eucalyptol) (0.9%) as minor products with GPP as substrate and no product with FPP as substrate (Table 1, Figure S9). In GC/MS chromatograms, there are actually two co-eluting peaks in the MT04 limonene peak. The one that is smaller and comes earlier is limonene and the other one, which is bigger and comes later, looks like (*R*)-(+)-m-mentha-6,8-diene (sylvestrene) when considering both retention time and mass spectra. However, we are not sure if it is really (*R*)-(+)-m-mentha-6,8-diene (sylvestrene) or not because GPP is not easily converted to (*R*)-(+)-m-mentha-6,8-diene (sylvestrene) when considering the structure. Maybe the mass spectrum is not clear due to peak overlap.

**1,8-Cineole synthase (MT11)**

Expression of MT11 in BL21 Star (DE3) pMevT pMBI RIL also produced 1,8-cineole and *p*-menth-1-en-8-ol (α-terpineol), however, the peaks of α-pinene and β-pinene were not shown due to overall low concentrations of extracted terpenes (Figure S10 E-G). No visible peaks for sesquiterpenes were observed from pentane extracts of BL21 Star (DE3) pMevT pMBI expressing MT11.

**α-Phellandrene synthase (MT03)**

With GPP as a substrate MT03 synthesizes α-phellandrene (92.2%) as a major product and β-phellandrene (contains limonene) (3.8%), γ-terpinene (2.5%), α-terpinene (1.0%) and *p*-mentha-1,4(8)-diene (terpinolene) (0.5%) as minor products (Tbale 2, Figure 20). The β-phellandrene peak contains also small amounts of limonene. Limonene and β-phellandrene have only a 0.01 min retention time difference in our GC/MS analysis and they come out together. The mass spectrum of the β-phellandrene peak shows overall similarity with the β-phellandrene mass spectra from the library, however, *m/z* ions 67 and 68 are from limonene (Figure 20).

Compared to T-Rh 3M, the amounts of α-phellandrene of T-Rh 5M and T-Rh 7M are 93.9% and 67.6% respectively.

Although ginger contains α-phellandrene, it is not a major monoterpene in ginger. However the expression level of MT10 (=MT03, Table S4) from microarray data is higher than turmeric. Because the microarray probe design was done before terpene synthase RACE and cloning, it is possible that the probe for MT10 is not a unique probe. Before RACE, MT03 unitrans was full length with one frameshift, whereas many other terpene synthase unitrans including MT10 were partial. So the MT10 microarray probe was not able to be checked against missing parts of other partial terpene synthases.

***p*-Mentha-1,4(8)-diene (terpinolene) synthase (MT07)**

With GPP as a substrate, MT07 synthesized *p*-mentha-1,4(8)-diene (terpinolene) (89.9%) as the major product and α-terpinene (4.3%), α-phellandrene (2.7%), limonene (1.5%), 3-carene; 4 (1.2%) and γ-terpinene (0.4%) as minor products (Table 1, Figure 21).

**(S)-(+)-Linalool/nerolidol synthase (MT00) and**

**(R)-(-)-linalool/(Z)-α-bisabolene/trans-α-bergamotene synthase (MT17A2)**

Acetate forms of linalool and (*E*)-nerolidol were also produced during MT00 enzyme assays using crude protein extracts and some proteins from *E. coli* may be involved in adding the acetate (Figure 22). No acetylated form of linalool and (*E*)-nerolidol was observed in the enzyme assay using purified MT00, where most *E. coli* proteins are eliminated. Expression of MT00 in BL21 Star (DE3) pMevT pMBI RIL also produced linalool and (*E*)-nerolidol. Even without IPTG induction, linalool and (*E*)-nerolidol were synthesized and IPTG induction elevated terpene production. When MT00 was expressed in BL21 Star (DE3) pMevT pMBI RIL cells, the MT00 gene was cloned into the pCRT7CT-TOPO vector, and the T7 promoter in this vector is not tightly regulated due to lack of *lac* operator. We could see leaky expression in protein gels and resulting terpene production.

MT17A2 produced small amounts of different sesquiterpenes such as cis-α-bisabolene (22.7%), trans-α-bergamotene (20.6%), β-bisabolene (13.6%), epi-α-bisabolol (12.6%), cis-α-bergamotene (7.6%), α-bisabolol (7.3%), (*E*)-nerolidol (5.8%), β-sesquiphellandrene (3.7%), unknown (cis-α-bisabolene-like) (2.1%), γ-curcumene (1.6%), unknown (β-sesquiphellandrene-like) (1.5%) and unknown (7-epi-sesquithujene-like) (0.8%) with FPP as substrate (Figure 23). The mass spectra of three unknown compounds are very similar to either cis-α-bisabolene, β-sesquiphellandrene or 7-epi-sesquithujene. However, the retention time of these compounds do not match the retention times of those compounds in the library. Nevertheless, the mass spectrum of the unknown peak (7-epi-sesquithujene-like) is very similar to that of 7-epi-sesquithujene and the retention time is also close to that of 7-epi-sesquithujene.

The β-bisabolene peak from *E. coli* protein assays also contained (*E*,*E*)-α-farnesene. The (*E*,*E*)-α-farnesene peak comes earlier and β-bisabolene peak comes slightly later in this overlapping peak. The peak area of β-bisabolene was adjusted after subtracting (*E*,*E*)-α-farnesene peak area in the chromatogram without MT17A2 (Figure 23 D2) from the β-bisabolene peak containing (*E*,*E*)-α-farnesene in the chromatogram with MT17A2 (Figure 23 E2). Control enzyme assay without pEXP5CT-MT17A2 also produced (*E*)-nerolidol, and the (*E*)-nerolidol peak in the chromatogram with MT17A2 (Figure 23 E2) is also calculated in the same way subtracting (*E*)-nerolidol in the chromatogram without MT17A2 (Figure 23 D2).

**Sabinene/nerolidol synthase (MT06 and MT06A)**

In ginger and turmeric, there are many small terpene peaks and many of them are considered to be by-products of terpene synthases that produce the more abundant terpenes. The evolution of terpene synthases sometimes leads to different or diverse products and/or incomplete enzyme activity. MT06 and MT06A may belong to this category. Although their solubility was small or medium (Table S5), the amount of products produced by enzyme assays was low; the peak height is similar to the myrcene or (*E*)-β-farnesene peaks synthesized by *E. coli* (Figures S15 and S1). This low activity was normally seen when the solubility was very low. The observation that the activity of MT06/MT06A seemed low and they produced many different compounds suggests that MT06/MT06A may be evolving.

When these cDNAs were cloned into the pEXP5CT-TOPO expression vector, transit peptides were removed, with truncated MT06 and MT06A being identical. MT06 and MT06A produce a variety of monoterpenes: sabinene (4(10)-thujene) (29.0%), linalool (24.8%), γ-terpinene (9.0%), α-terpinene (8.2%), *p*-menth-1-en-4-ol (terpinen-4-ol) (8.0%), cis-sabinene hydrate (6.2%), *p*-menth-1-en-8-ol (α-terpineol) (6.2%), *p*-mentha-1,4(8)-diene (terpinolene) (5.2%) and α-thujene (3-thujene) (3.4%) (Table 1, Figure 24), and variety of sesquiterpenes; (*E*)-nerolidol (41.0%), epi-β-bisabolol (22.3%), β-curcumeme (14.5%), unknown (7-epi-sesquithujene-like) (8.1%), unknown (cis-sesquisabinene hydrate-like) (4.5%), γ-curcumene (4.4%), β-sesquiphellandrene (1.9%), epi-α-bisabolol (1.6%), unknown (trans-sesquisabinene hydrate-like1) (1.2%), (*E*)-γ-bisabolene (0.6%) (Table 2, Figure 25). Although control *E. coli* without MT06/MT06A also produces linalool and (*E*)-nerolidol, MT06 and MT06A the levels of linalool and (*E*)-nerolidol were elevated compared to corresponding peaks of the control. Peaks referred to as unknown (7-epi-sesquithujene-like), unknown (cis-sesquisabinene hydrate-like) and unknown (trans-sesquisabinene hydrate-like1) had mass spectra that were very similar to 7-epi-sesquithujene, cis-sesquisabinene hydrate and trans-sesquisabinene hydrate, however, the retention time for these samples deviated slightly from the retention time listed in the library database (Figure 26E).

**epi-α-Bisabolol/α-bisabolol synthase (MT02A)**

After RACE, we tried to clone the MT02 cDNA, however, we could clone only MT02A. MT02A was 95.1% identical in DNA sequence with MT02.

**β-Selinene (eudesma-4(14),11-diene) synthase (ST01)**

The β-selinene (eudesma-4(14),11-diene) peak has a similar retention time to α-zingiberene. Unlike α-humulene that comes 0.1 minute later than the huge α-zingiberene peak, the β-selinene peak comes 0.15 minutes earlier, so it is not covered by the α-zingiberene peak.

Enzyme assay with ST01 synthesized β-selinene (eudesma-4(14),11-diene) (51.9%) as the major product and 7-epi-α-selinene (14.2%), unknown (eremophila-1(10),11-diene-like) (11.3%), β-elemene (9.7%), 5, unknown (β-chamigrene-like) (7.4%), unknown (guaia-1(5),7(11)-diene-like) (3.4%) and (+)-intermedeol (2.1%) as minor products (Table 2, Figure 27).

ST01 unitrans has ESTs in the yellow ginger rhizome cDNA library and was cloned from yellow ginger rhizome (Table S3).

**(-)-Neointermedeol synthase (ST02A4), α-elemol ((+)-hedycaryol) synthase (ST02B) and β-elemene (germacrene A) synthase (ST02C)**

ST02B and ST02C were soluble in *E. coli*. ST02A4 was insoluble in both *E. coli* and yeast when checked by stained protein gels (for *E. coli*) and Western blots (for yeast), however, yeast expressing ST02A4 produced very small amount of products.

Pentane extracts of yeast strain EPY224 expressing ST02A4 produced (-)-neointermedeol (48.7%) as a major product and β-elemene (12.6%), α-cadinol (7.5%), unknown (cubenol-like) (6.5%), germacrene D (6.1%), epi-α-muurolol (τ-muurolol) (6.0%), α-muurolol (δ-cadinol) (2.5%), unknown (selina-6-en-4-ol-like) (2.4%), α-muurolene (1.6%), δ-cadinene (cadina-1(10),4-diene) (1.2%), δ-elemene (1.2%), γ-elemene (1.1%), γ-eudesmol (0.8%), (+)-intermedeol (0.8%), (*E*)-caryophyllene (0.5%) and unknown (spathulenol-like) (0.4%) as minor products (Table 2, Figure 28).

Enzyme assays using *E. coli* crude extract expressing ST02B with GPP as substrate produced several monoterpenes: linalool (25.1%), myrcene (25.0%), limonene (15.5%), (*Z*)-β-ocimene (14.3%), (E)-β-ocimene (9.8%), *p*-mentha-1,4(8)-diene (terpinolene) (7.7%) and *p*-menth-1-en-8-ol (α-terpineol) (2.7%) (Table 1, Figure 29). Because *E. coli* crude extracts without the pET101/D-ST02B plasmid also produced myrcene with GPP as substrate, the area of the myrcene peak in the control was subtracted from the area of myrcene peak in the ST02B experiment. With FPP as substrate, enzyme assays using *E. coli* crude extracts expressing ST02B also produced several sesquiterpenes. Enzyme assays were performed with overlaid pentane. After 3 hours of 30 ℃ incubation, the top pentane was directly injected into the GC/MS or whole enzyme assays including the remaining top pentane were vortexed and centrifuged and the collected pentane was injected into the GC/MS. Normally both total ion chromatograms using the top pentane or the vortexed pentane showed very similar profiles, however, ST02B showed different ratios of products. The major difference was the amount of α-elemol, which represented 60.5% of synthesized sesquiterpenes in the top overlaid pentane but only 25.3% in vortexed samples. When the peak areas from both the top and vortexed pentane were summed, α-elemol was 44.3% of all products. During enzyme assays, synthesized terpenes are captured in top pentane layer and synthesized terpenes in the enzyme assay buffer are collected by vortexing with the pentane. So the product ratio is calculated by the sum of both peak areas from the top pentane and vortexed pentane, which suggested that ST02B utilizes FPP and synthesizes α-elemol (44.3%), β-elemene (18.3%), α-copaene (11.2%), unknown (cyclosativene-like) (7.8%), γ-elemene (6.1%), germacrene B (4.9%), δ-cadinene (cadina-1(10),4-diene) (2.8%), α-muurolene (3.1%) and germacrene D (1.6%) (Table 2, Figure S20). The retention time of the peak, unknown ((+)-cyclosativene-like), was 18.14 and there was a trace amount of (+)-cyclosativene at RT 18.21 although it was not seen clearly in the figure because the peak was small.

Enzyme assays using *E. coli* crude extracts expressing ST02C with GPP as a substrate produced several monoterpenes: myrcene (30.4%), limonene (17.9%), linalool (15.6%), (*Z*)-β-ocimene (13.5%), *p*-mentha-1,4(8)-diene (terpinolene) (9.9%), (E)-β-ocimene (9.3%) and *p*-menth-1-en-8-ol (α-terpineol) (3.3%) (Table 1, Figure S21). Because *E. coli* crude extracts without the pET101/D-ST02C plasmid also produced myrcene with GPP as substrate, the area of the myrcene peak in the control was subtracted from the area of myrcene peak in the ST02C experiment. Enzyme assays using *E. coli* crude extracts expressing ST02C with FPP as substrate produced β-elemene (49.3%) as the major product and germacrene D (12.4%), α-muurolene (9.5%), γ-muurolene (4.7%), δ-cadinene (cadina-1(10),4-diene) (4.0%), (+)-cyclosativene (3.2%), germacrene B (2.9%), δ-elemene (2.9%), γ-elemene (2.7%), (*E*)-caryophyllene (2.4%), cis-β-elemene (2.1%), unknown ((+)-cyclosativene-ike) (2.1%) and α-copaene (1.7%) as minor products (Table 2, Figure S22). Unlike ST02B, both the overlaid top pentane and vortexed pentane from the ST02C enzyme assay had β-elemene as a major product.

Monoterpene products were very similar between ST02B and ST02C, however, sesquiterpenes product profiles were different in these two proteins. ST02C made β-elemene as the major product and ST02B synthesized α-elemol as the major product although ST02B also produced β-elemene. The homology percent between ST02B and ST02C is 96.0% in DNA sequence and 95.7% in amino acids sequence. ST02A4 is 95.3% identical with ST02B and 96.0% with ST02C in amino acid sequence.

**γ-Amorphene synthase (ST03)**

Enzyme assays using crude *E. coli* extracts with GPP yielded linalool (36.8%), myrcene (27.3%), (*Z*)-β-ocimene (17.6%), *p*-mentha-1,4(8)-diene (terpinolene) (5.2%), cis-*p*-menth-2en-1-ol (3.8%), (*E*)-β-ocimene (3.5%) and *p*-menth-1-en-8-ol (α-terpineol) (3.3%) (Table 1, Figure S23).

**α-Humulene (α-caryophyllene) synthase (ST05 and ST05A)**

ST05A showed a similar profile to ST05: α-humulene (α-caryophyllene) (88.4%) as the major product with (*E*)-caryophyllene (β-caryophyllene) (10.5%) and β-elemene (1.0%) as minor products (Table 2, Figure S25 D).

**(-)-Caryolan-1-ol synthase (ST07 and ST07A)**

EPY224 with ST07A expression mainly produced (-)-caryolan-1-ol (99.5%) and trace amounts of α-humulene (α-caryophyllene) (0.4%) and (*E*)-caryophyllene (0.2%) (Table 2, Figure S26). Yeast strain EPY224 is designed to produce more FPP. The yeast itself produces farnesenes, (*E*)-nerolidol and farnesol. With exogenous terpene synthase expression, competition over the FPP pool occurs and yeast derived farnesenes, (*E*)-nerolidol and farnesol production is limited when exogenous terpene synthases utilize most of the FPP, which explains why (*Z*)-β-farnesene, (*E*)-nerolidol and farnesol peaks are not seen in the sample with ST07A expression in Figure S26. After closer comparison to extractions from yeast expressing ST07 and ST07A, we could find very small product peaks in yeast extractions expressing ST07. Like ST07A, ST07 also produced (-)-caryolan-1-ol (98.7%) and (*E*)-caryophyllene (1.3%) (Table 2, Figure S27). There was a small peak at the same retention time as the (-)-caryolan-1-ol peak in the EPY219 control, however, this small peak in the EPY219 control did not represent (-)-caryolan-1-ol. The major ion in the peak from the EPY219 control was *m/z* 109, but the major ion in the (-)-caryolan-1-ol peak is *m/z* 111. We also checked the big background peak (peak 1 in Figure S27) to see if *m/z* 109 was a contaminant from the background and found it was not a dominant *m/z* in the big background peak of the EPY219 control, which suggested that EPY219 itself does not produce (-)-caryolan-1-ol and the (-)-caryolan-1-ol in EPY219 expressing ST07 came from ST07. Because ST07A also produces (*E*)-caryophyllene, we checked if ST07 also produces (*E*)-caryophyllene. There was a very small peak at the same retention time with the (*E*)-caryophyllene peak in the ST07A sample and its mass spectrum and single ion chromatogram with *m/z* 91, which is most abundant ion in (*E*)-caryophyllene, suggest that this very small peak in ST07 is also (*E*)-caryophyllene. However, α-humulene (α-caryophyllene) peak is imbedded in the large (*Z*)-β-farnesene peak because soluble ST07 was not even detected in yeast by Western blotting (Table S5) and *E. coli* proteins used most of the FPP to make farnesenes, (*E*)-nerolidol and farnesol.

Although the ST07 unitrans has ESTs from white ginger rhizome cDNA library, ST07 and ST07A were cloned from white ginger root cDNA. ST07 is not expressed in turmeric but high in ginger, especially root and leaf tissues according to the microarray data (Table S4). These expression patterns were similar to (*E*)-caryophyllene production patterns in ginger and turmeric tissues, which suggests that paralogs of ST07 that synthesize (*E*)-caryophyllene exist and are expressed highly in ginger root and leaf tissues.
